# Magnetically recoverable WFe_2_O_4_/rGO catalyzed green synthesis of thiadiazolo[2,3-*b*] quinazolin-6(7*H*)-one derivatives: structural, DFT, and molecular docking studies

**DOI:** 10.1039/d6ra06264d

**Published:** 2026-09-10

**Authors:** Varsharani S. More, Vaibhav B. Sankpal, Asha D. Patil, Santosh S. Chobe, Kuldeep T. Padhyar, Navanand B. Wadwale, Vijay N. Bhosale, Shrikant S. Nilewar, Sandeep G. Sontakke, Lalit D. Bhosale, Sambhaji R. Mane, Gopinath S. Khansole

**Affiliations:** a Department of Chemistry, K. R. P. Kanya Mahavidyalaya Urun-Ishwarpur Sangli-415409 M.S. India; b Department of Chemistry, Deshbhakt Anandrao Balwantrao Naik Arts and Science College Chikhali Sangli-415408 M.S. India gopinathskhansole@gmail.com; c Department of Physics, Deshbhakt Anandrao Balwantrao Naik Arts and Science College Chikhali Sangli-415408 M.S. India; d Department of Chemistry, Research Centre, M. G. V.'s Loknete Vyankatrao Hiray Arts, Science and Commerce College Panchavati Nashik-03 M.S. 422003 India; e Department of Chemistry, P. G. Research Centre, M. S. G. College Malegaon Camp, Malegaon Nashik M.S. 423105 India; f Department of Chemistry, P. G. Research Centre, Yeshwant Mahavidyalaya Nanded M.S. 431602 India; g Department of Pharmaceutical Chemistry, Maliba Pharmacy College, Uka Tarsadia University Gujrat 394350 India; h Department of Chemistry, Lonavala Education Trust's Dr B. N. Purandare Arts, Smt. S. G. Gupta Commerce and Smt. Shardaben Amrutlal Mithaiwala Science College Lonavala 410403 Pune M.S. India; i Department of Chemistry, Yashwantrao Chavan Warana Mahavidyalaya, Warananagar University Warana Nagar M.S. 416 114 India

## Abstract

A facile, one-pot multicomponent protocol is reported for the synthesis of biologically active thiadiazolo[2,3-*b*] quinazolin-6(7*H*)-one derivatives from 2-amino-5-phenyl-1,3,4-thiadiazole, dimedone, and substituted aromatic aldehydes, catalyzed by magnetic WFe_2_O_4_/rGO NPs synthesized *via* a hydrothermal route. The catalyst, characterized by FT-IR, XRD, SEM, EDX, BET, VSM, TGA, XPS, Raman spectroscopy and zeta potential, afforded the target heterocycles in short reaction times and high yields at room temperature, with easy magnetic recovery and excellent reusability. Molecular docking of compounds V1–V6 revealed favorable computational binding affinities against DNA gyrase as a putative target, while DFT calculations on V1, V2, V5, and V7 (B3LYP/6-311++G(d,p)) provided optimized geometries, thermodynamic parameters, global reactivity descriptors, and HOMO–LUMO energy gaps. Together, these experimental and computational results establish WFe_2_O_4_/rGO NPs as an efficient, sustainable catalyst for green heterocyclic synthesis and highlight the preliminary antibacterial properties of the synthesized thiadiazolo quinazoline derivatives.

## Abstract 2

A magnetically recoverable WFe_2_O_4_/rGO nanocatalyst enables green, one-pot synthesis of thiadiazolo[2,3-*b*]quinazolin-6(7*H*)-one derivatives, which show promising antibacterial activity rationalized through molecular docking and DFT studies. Image created with the assistance of Google Gemini.

## Graphical abstract

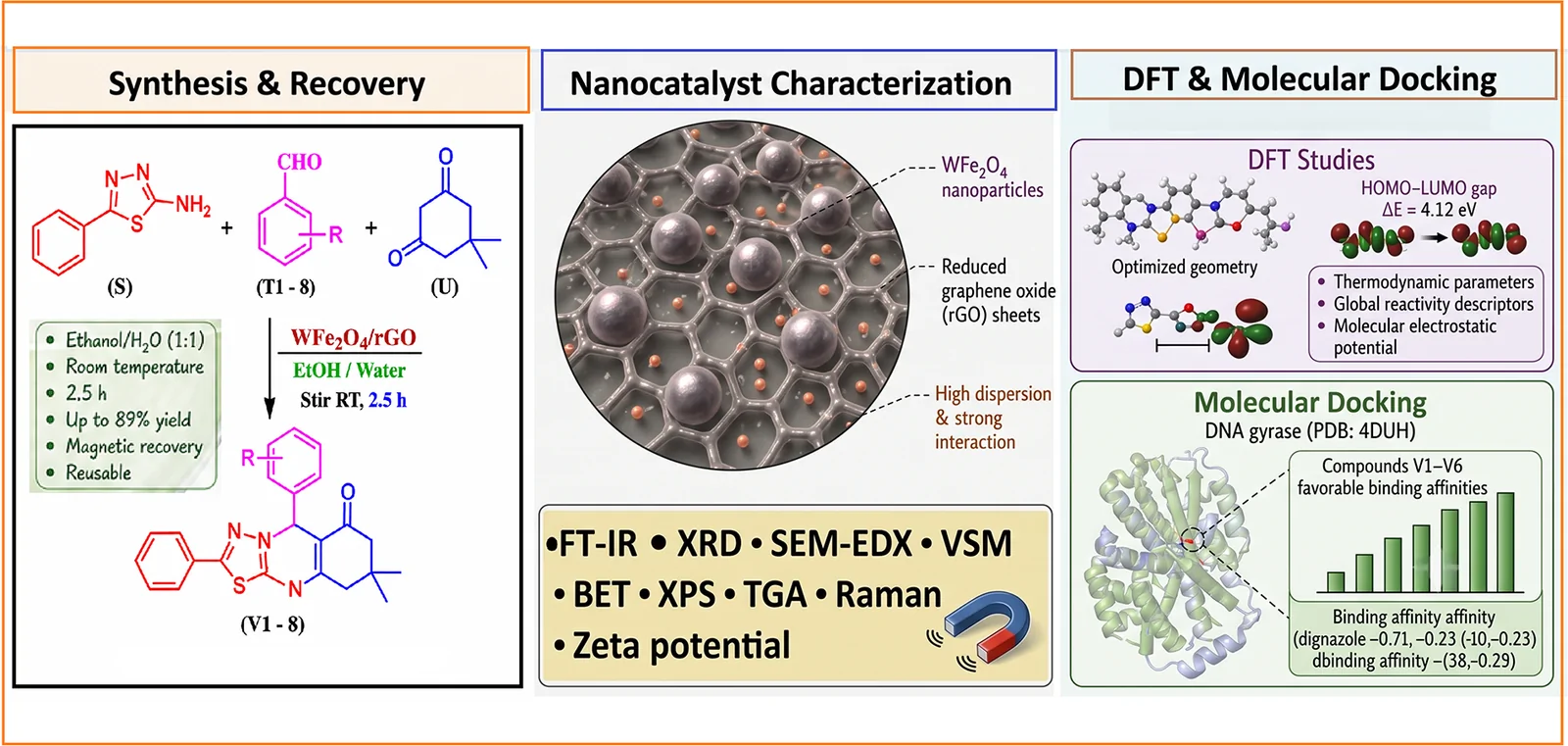

## 1 Introduction

The development of efficient, sustainable, and reusable catalytic systems remains a central goal in modern synthetic chemistry, particularly for the construction of biologically relevant heterocycles. Heterogeneous nanocatalysts have therefore attracted growing interest as alternatives, offering high surface-to-volume ratios, tunable active sites, and the possibility of straightforward separation and reuse.^1,2^ Among these, magnetic nanocomposites have emerged as particularly attractive platforms, since the incorporation of a ferromagnetic or ferrimagnetic component allows the catalyst to be recovered from the reaction mixture using a simple external magnet, eliminating the need for filtration or centrifugation and minimizing catalyst loss across cycles.^3,4^ Reduced Graphene Oxide (rGO) has been an extremely successful material for improving the catalytic performance of metal oxide nanocatalysts. In addition to providing a large surface area upon which nanoparticles can be anchored to prevent aggregation, rGO's 2D sheet structure also allows for fast electron transport throughout the conjugated π-system.^5^ When coupled with transition metal ferrites, these provide redox activity and magnetic recoverability while rGO offers structural stability, conductivity and high dispersion capacity for hybrid composites that are ideal for multicomponent organic transformations under mild conditions with minimal environmental impact.^6–12^ While there is significant potential for the application of such hybrid composite materials, including those containing tungsten-based ferrites, they remain relatively uninvestigated as heterocycle synthesis catalysts.^13^ Therefore, we aim to investigate both their physical/chemical properties and their catalytic behavior in this field.

The multicomponent reaction has become an increasingly popular approach within contemporary chemical synthesis due to its efficiency and ability to produce highly functionalized molecular structures in one step using commercially accessible starting materials. As such, it fits into the tenets of Green Chemistry.^14–16^ The fused nitrogen–sulfur heterocycles continue to be of substantial interest in medicinal chemistry because of the broad pharmacological profile associated with the thiadiazole and quinazoline pharmacophores individually,^17,18^ and the enhanced bioactivity often observed when these rings are fused into a single scaffold. Thiadiazoloquinazoline derivatives, formed through annulation of a 1,3,4-thiadiazole ring with a quinazolinone core, present a rigid, electron-rich framework capable of engaging multiple types of non-covalent interactions with biological targets. Compounds bearing this scaffold have been reported to display antibacterial, antifungal, anticancer, anti-inflammatory, antiviral, and anti-HIV activities,^19–25^ and their multicomponent synthesis from simple, readily available precursors makes them attractive targets for catalytic methodology development. Given the persistent clinical need for new antibacterial agents in the face of rising resistance,^26,27^ scaffolds with this breadth of reported activity merit continued synthetic and mechanistic investigation. A recurring limitation in structure–activity studies of such heterocyclic libraries, however, is the frequent reliance on a single computational metric, typically a docking score, to rationalize observed biological activity. Some bioactive fused thiadiazoloquinazoline derivatives are displayed in Fig. 1(a–c).

**Fig. 1:**
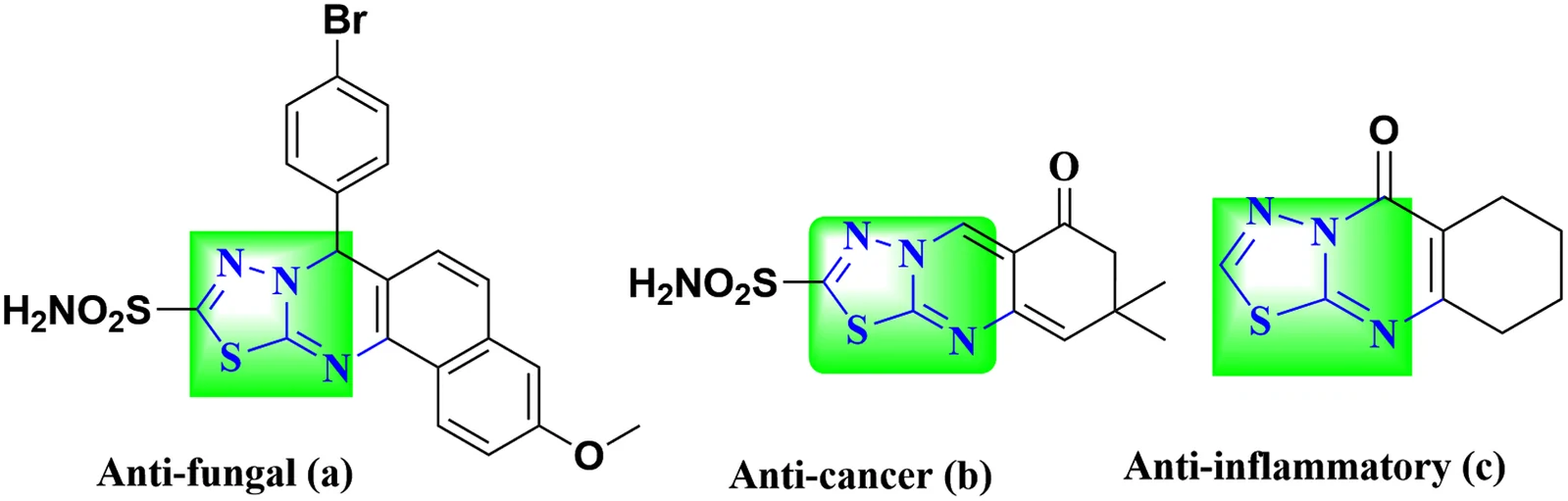
(a–c) Examples of bioactive thiadiazoloquinazoline derivatives documented in the literature.

To date, the common synthetic methods to access fused thiadiazoloquinazoline scaffolds involve reactions between 2-amino-1,3,4-thiadiazole-5-sulfonamide with benzylidenetetralones in propylene glycol at 200–220 °C for 12 h,^28^ the reaction of 5-(2-aminophenyl)-1,3,4-oxadiazole-2-thione with tetramethylthiuram disulfide at 205–210 °C; the reaction of 3-amino-2-mercapto-3*H*-quinazoline-4-one with dimethyl acetylenedicarboxylate in DMF at reflux for 18 h,^29^ the reaction of 2-amino-5-phenyl-1,3,4-thiadiazole with malonitrile for 12 h,^30^ the reaction of diazotized anthranilic acid and its methyl ester with substituted α-thiocyanatoacetoacetanilides,^30^ and condensation of 2-amino-5-phenyl-1,3,4-thiadiazole, dimedone and tetrabutylammonium hydrogen sulfate in water–ethanol with aromatic aldehydes refluxed at 60 °C.^31^ The above methods suffer from multistep synthesis, poor yield, expensive starting materials, corrosive reagents, prolonged reaction times, and harsh reaction conditions.^30–32^ Therefore, given the biological activities and diverse applications of thiadiazoloquinazolinones, a diversity-oriented multicomponent synthetic strategy for this scaffold is well worth pursuing. In this work, we report the design and hydrothermal synthesis of magnetically recoverable WFe_2_O_4_/rGO NPs and their evaluation as a heterogeneous catalyst for the one-pot, three-component synthesis of thiadiazolo[2,3-*b*] quinazolin-6(7*H*)-one derivatives from 2-amino-5-phenyl-1,3,4-thiadiazole, dimedone, and substituted aromatic aldehydes in an ethanol–water medium at room temperature. The catalyst was characterized by FT-IR, XRD, SEM, EDX, VSM, BET, TGA, Raman spectroscopy, zeta potential and XPS analysis to establish its structural, morphological, and magnetic properties, and its reusability and true heterogeneous character were confirmed through recyclability and hot filtration studies.^33,34^ The synthesized derivatives were evaluated for antibacterial activity, and molecular docking against a representative putative computational target (DNA gyrase, PDB: 4DUH) was performed to probe the structural basis of this activity.^26,27,35^ To reconcile cases where docking scores and experimental antibacterial phenotypes diverged, selected compounds were further examined by molecular dynamics simulation to capture the role of binding-site dynamics and ligand flexibility in determining biological outcome.^36–38^ Finally, DFT calculations at the B3LYP/6-311++G(d,p) level were carried out on representative derivatives to obtain optimized geometries, frontier molecular orbitals, global reactivity descriptors, and electrostatic potential maps, providing electronic-structure context for the observed reactivity and structure–activity trends.^39–43^ These experiments and computations, together, demonstrate that WFe_2_O_4_/rGO NPs are effective and can be used repeatedly in a magnetic separator as a catalyst for green heterocycle formation, and provide a mechanistic basis for how structural characteristics of molecules affect their bactericidal properties within the thiadiazoloquinazolines.

## 2 Results and discussion

### 2.1. Characterization of the prepared heterogeneous WFe_2_O_4_/rGO NPs catalyst

The WFe_2_O_4_/rGO catalyst was investigated using infrared spectroscopy. Fig. 2 shows the FT-IR spectra of WFe_2_O_4_ (a), WFe_2_O_4_/rGO (b), and reused WFe_2_O_4_/rGO after the fifth catalytic run (c), in the scanning range of 4000–500 cm^−1^. In Fig. 2a, characteristic bands at 756 cm^−1^ and 820 cm^−1^ are attributed to W–O and Fe–O stretching vibrations in WFe_2_O_4_, respectively.^5,48^

**Fig. 2:**
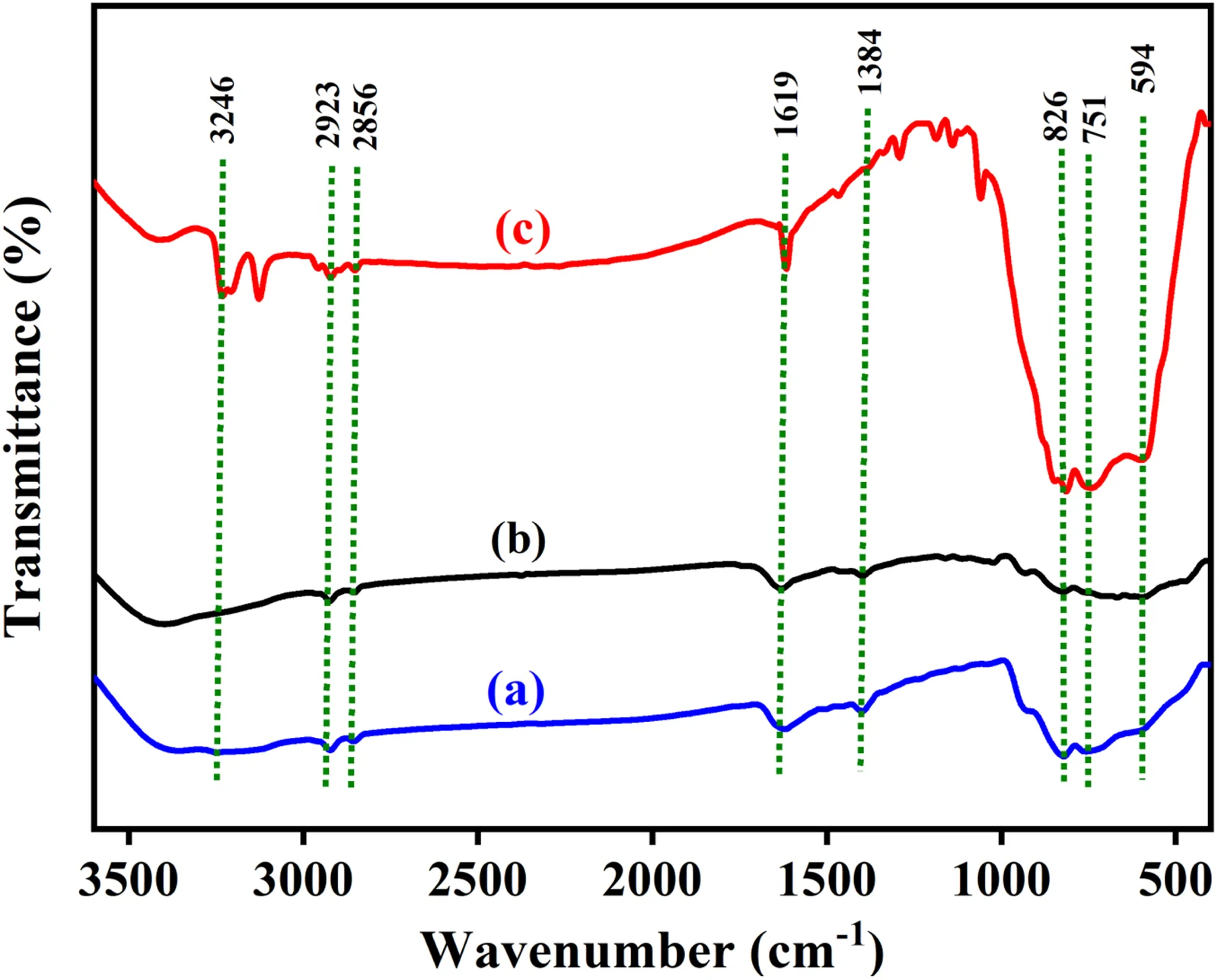
FT-IR spectra of (a) WFe_2_O_4_, (b) WFe_2_O_4_/rGO, and (c) reused WFe_2_O_4_/rGO after the fifth catalytic run.

Fig. 2b shows that the WFe_2_O_4_/rGO composite retains the combined spectral features of both precursors. The bands at 3246 cm^−1^ and 2923 cm^−1^ are attributed to the free –OH stretching frequency of hydroxyl groups in reduced graphene oxide. The strong band at 1619 cm^−1^ corresponds to C=C stretching vibration, while the bands at 1240 cm^−1^ and 1119 cm^−1^ are due to C–O–C and C–O stretching vibrations. The bands at 820 cm^−1^ (Fe–O stretching) and 756 cm^−1^ (W–O stretching) are also retained in the composite, confirming the interaction between WFe_2_O_4_ and the reduced graphene oxide sheets.^5–7^Fig. 2c shows the FT-IR spectrum of the catalyst reused after the fifth successive catalytic run; no substantial loss or shift of the characteristic W–O, Fe–O, and carbon-framework bands is observed, indicating that the chemical bonding environment of the WFe_2_O_4_/rGO nanocomposite is retained after repeated catalytic use.

X-ray diffraction (XRD) analysis was employed to assess the crystal structure, phase purity, and crystallographic orientation of the synthesized rGO, pristine WFe_2_O_4_, and the WFe_2_O_4_/rGO nanocomposite (both fresh and reused), recorded over the 2*θ* range of 20° to 65°, as shown in Fig. 3(a–d). The diffraction pattern of rGO shows a prominent peak centered at 2*θ* = 26.5°, corresponding to the (002) crystallographic plane of graphitic carbon, confirming the successful reduction of graphene oxide and restoration of the graphitic structure.^49–51,98^

**Fig. 3:**
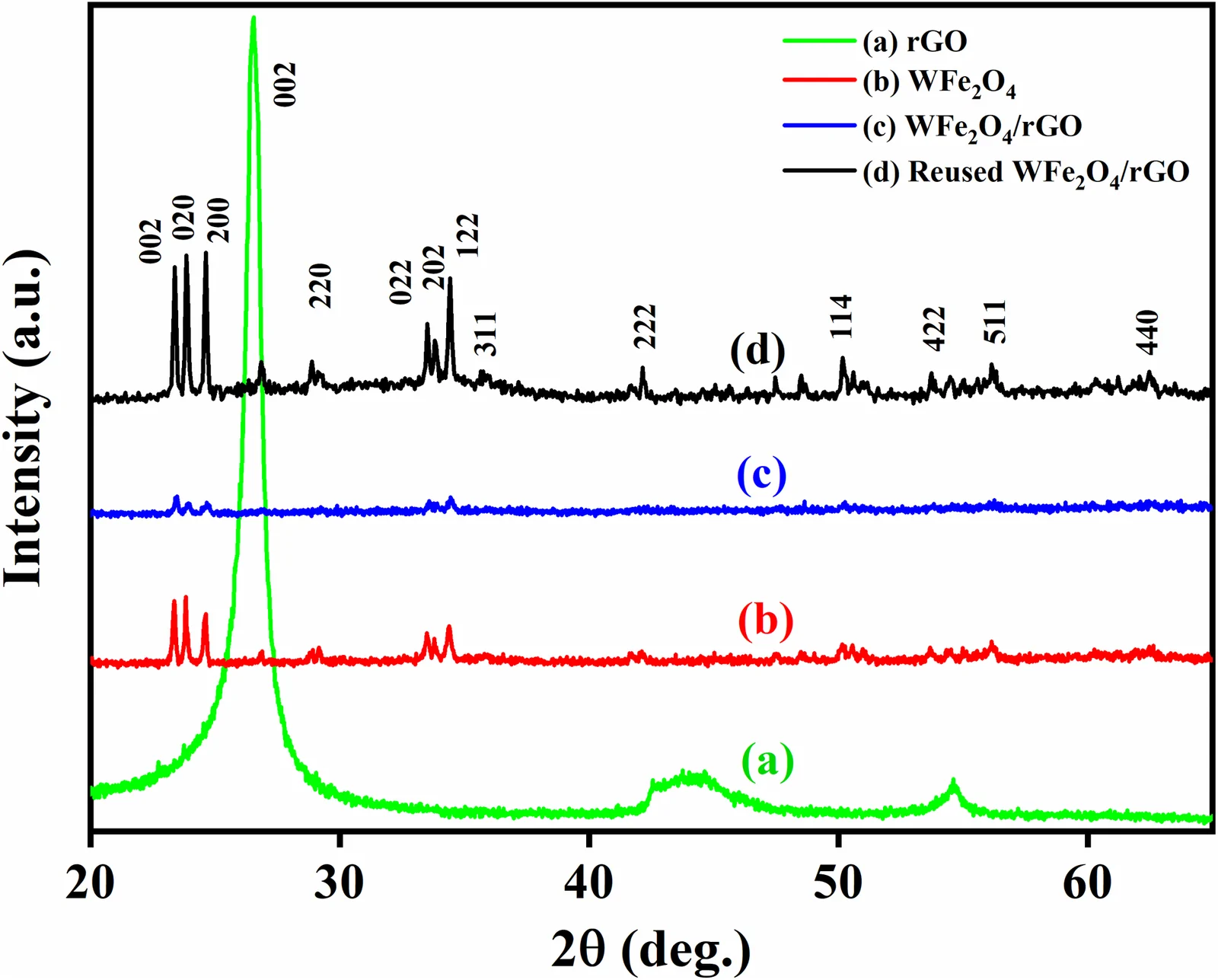
X-ray diffraction (XRD) patterns of (a) rGO, (b) WFe_2_O_4_, (c) WFe_2_O_4_/rGO, and (d) reused WFe_2_O_4_/rGO after the fifth catalytic run.

For pristine WFe_2_O_4_, well-resolved and sharp diffraction peaks confirm a high degree of crystallinity. Two triplet-like reflection sets are observed at lower diffraction angles: the first, at 2*θ* = 23.36°, 23.87°, and 24.63°, is indexed to the (002), (020), and (200) lattice planes, while the second, at 33.51°, 33.87°, and 34.46°, corresponds to the (022), (202), and (122) planes, respectively. Additional reflections are observed at 29.20° (220), 35.71° (311), 42.14° (222), 50.17° (114), 54.50° (422), 56.53° (511), and 62.48° (440), in good agreement with the standard JCPDS/PDF card no. 46-1446, confirming the formation of a crystalline spinel ferrite phase.^52,54,55,99^ The absence of extra diffraction peaks indicates that no secondary phases or impurity compounds were formed, and the sharp, intense reflections indicate a high degree of crystallinity of the ferrite nanoparticles.

Upon incorporation of rGO into the ferrite, the diffraction pattern of the WFe_2_O_4_/rGO nanocomposite retains all primary reflections of the parent metal oxide, alongside the characteristic carbon (002) reflection overlapping near 2*θ* = 26.5°, indicating that the fundamental crystal structure of the ferrite remains unchanged after composite formation.^7,9,56,63,97^ To evaluate structural stability and recyclability, the XRD pattern of the reused WFe_2_O_4_/rGO catalyst (recovered after the fifth catalytic run) was compared against the fresh material. The diffraction pattern of the reused catalyst exhibits identical 2*θ* peak positions, preserving all characteristic reflections (002), (020), (200), (022), (202), (122), and (114) with no noticeable peak shifting or emergence of secondary crystalline phases. This confirms the exceptional structural integrity and phase stability of the catalyst, and its resistance to lattice degradation during repeated catalytic use.

The scanning electron microscopy was used to examine the surface structure and size distribution of the WFe_2_O_4_/rGO nanocatalyst (Fig. 4a–d). All the magnifications confirm the effective deposition of tungsten ferrite nanoparticles onto reduced graphene oxide sheets, indicating successful synthesis of WFe_2_O_4_/rGO nanocomposite. The SEM image (Fig. 4a and c) at low magnification shows relatively uniform distribution of WFe_2_O_4_ nanoparticles across the rGO sheet. The SEM image (Fig. 4b and d) at high magnification shows bright white spots and near-spherical magnetic WFe_2_O_4_ nanoparticles anchored across the rGO sheets. The rGO sheets provide a large surface area and prevent particle aggregation with a relatively core-shell-like surface structure.^5–7^

**Fig. 4:**
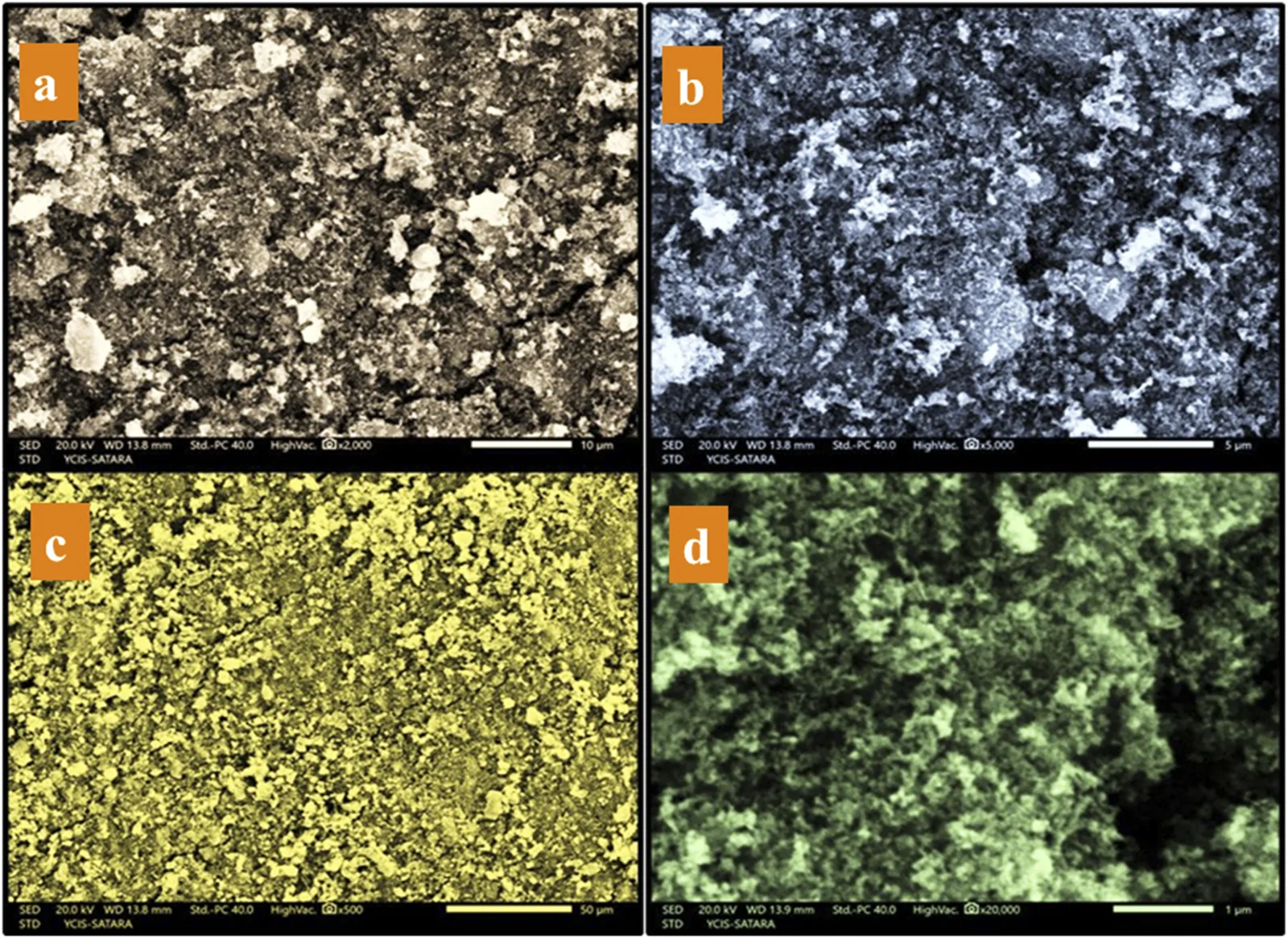
(a–d) SEM images of WFe_2_O_4_/rGO catalyst.

The EDX spectroscopy data were collected in the selected region of micrographs shown in Fig. 5. The EDS spectra of WFe_2_O_4_/rGO show the main peaks corresponding to the elements iron, tungsten (W), carbon (C), and oxygen (O) present on the surface of the magnetic support at percentage concentration Fe = 37.12%, W = 27.79%, C = 8.65%, and O = 26.44%, respectively. It is possible to verify that elements W and Fe are relatively well dispersed on the surface of the reduced graphene oxide, which indicates catalytic support, and this uniform distribution of W and Fe is essential for the catalytic activity of the magnetic catalyst development.

**Fig. 5:**
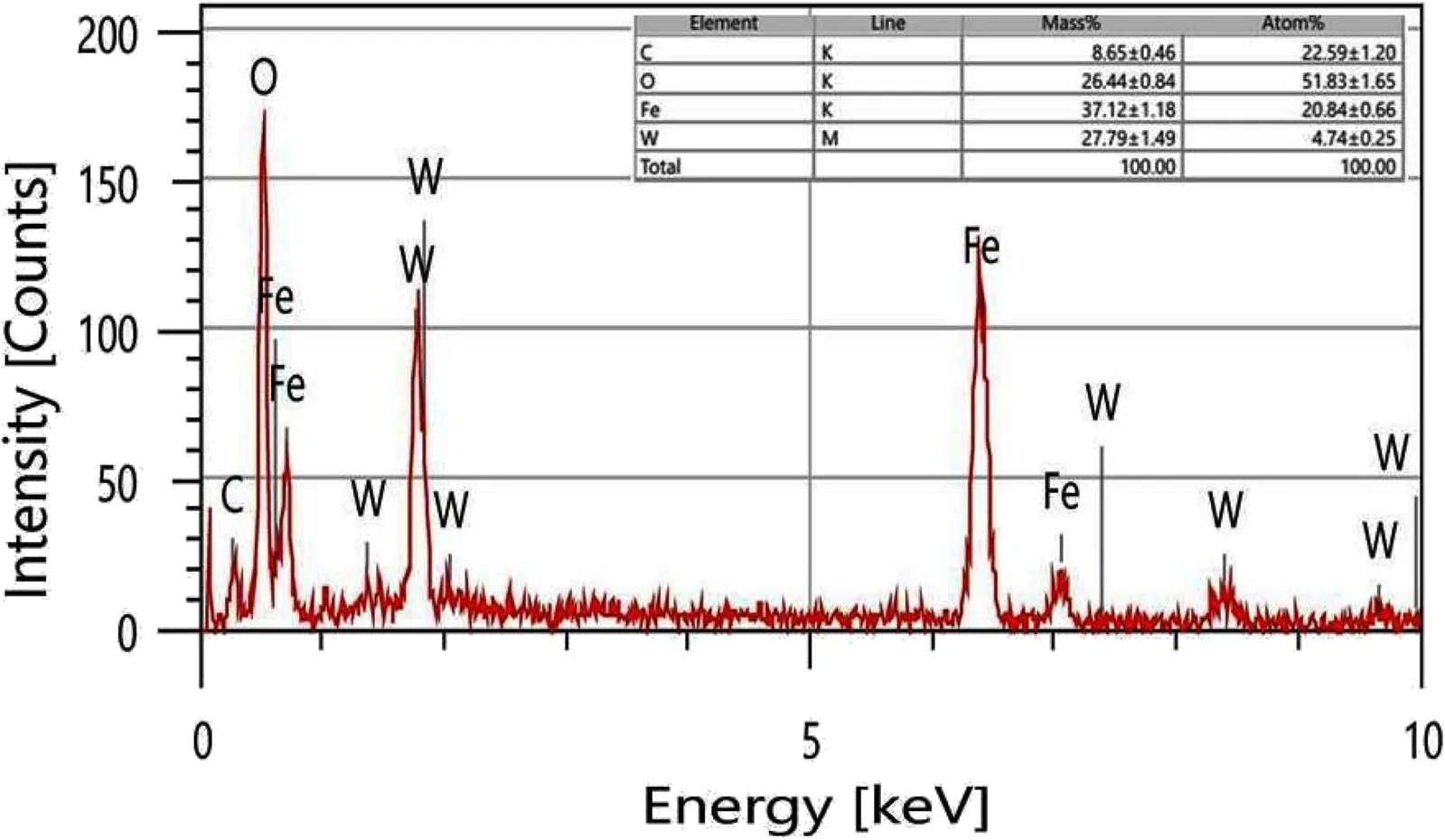
EDX analysis of WFe_2_O_4_/rGO nanocomposite.

The magnetic behavior of the synthesized samples (WFe_2_O_4_ and WFe_2_O_4_/rGO) was investigated using vibrating sample magnetometry.^44–46,64^ The magnetic hysteresis loop for WFe_2_O_4_ and WFe_2_O_4_/rGO at room temperature was displayed in Fig. 6(a and b), respectively. The pristine sample (WFe_2_O_4_) shows a narrow and symmetric hysteresis loop which confirms soft ferrimagnetic nature with low coercivity and remanence. The observed values for WFe_2_O_4_ sample exhibits a saturation magnetization of 1.43 emu g^−1^, remanent magnetization of 0.30 emu g^−1^, and coercivity of 210 Oe.^46,65^ Further, investigated WFe_2_O_4_/rGO sample reveals broader hysteresis loop. The incorporation of reduced graphene oxide (rGO) leads to a noticeable enhancement in the magnetic properties, a well-known effect reported in rGO–ferrite composites.^56,66^ The WFe_2_O_4_/rGO sample exhibits an increased *M*_s_ of 3.12 emu g^−1^, along with higher *M*_r_ (0.52 emu g^−1^). This improvement in *M*_s_ and *M*_r_ values can be attributed to the good dispersion of tungsten ferrite nanoparticles on the reduced graphene oxide sheets, which reduces agglomeration and promotes stronger magnetic interactions. However, the coercivity decreases to 161 Oe in the composite which suggests that the material becomes magnetically softer after rGO incorporation. In general, both samples exhibit soft magnetic behavior; however, the incorporation of rGO significantly improves the saturation magnetization which makes the composite more suitable for advanced magnetic and catalysis-related applications.

**Fig. 6:**
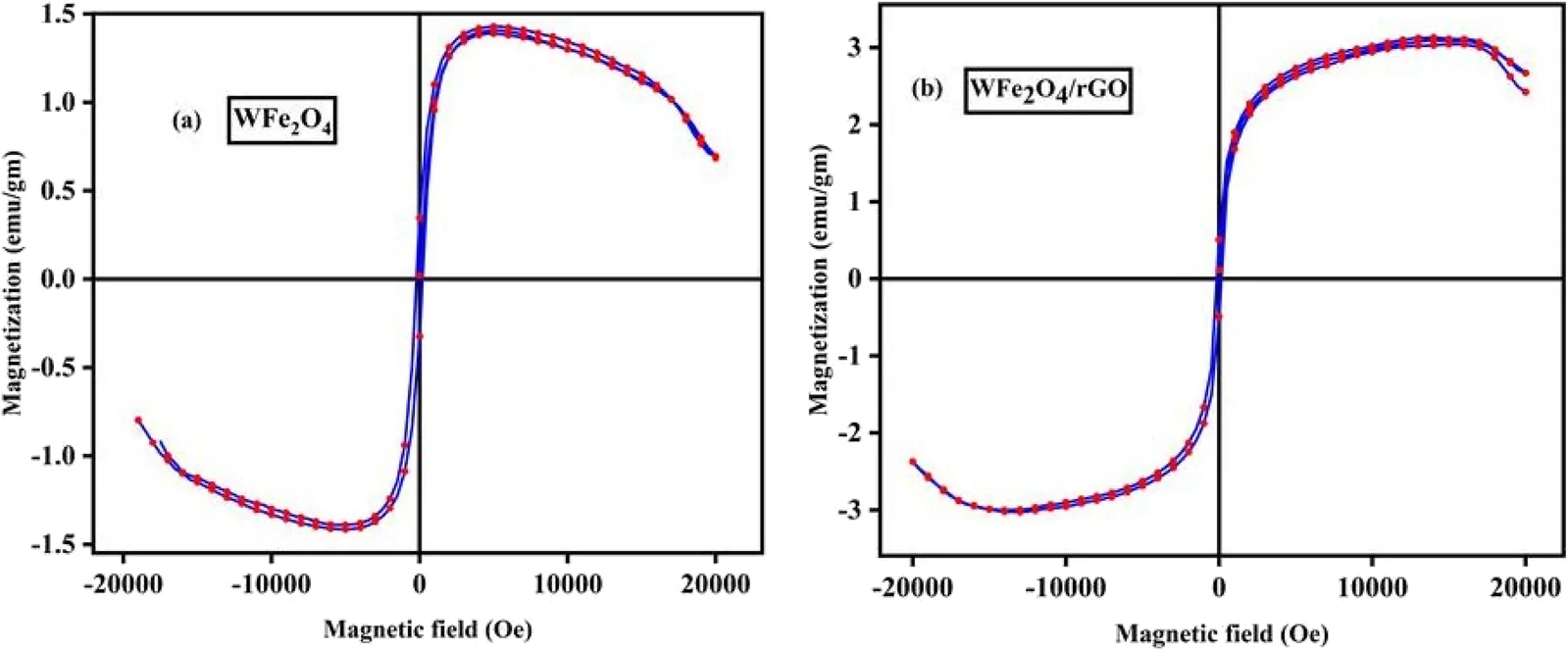
(a and b) Magnetization curve for WFe_2_O_4_ and WFe_2_O_4_/rGO.

The surface textural characteristics of the WFe_2_O_4_/rGO NPs were investigated using nitrogen adsorption–desorption measurements based on the Brunauer–Emmett–Teller theory.^67–71^ The N_2_ adsorption–desorption isotherm recorded at 77 K is shown in Fig. 7a, while the corresponding pore size distribution curve is presented in Fig. 7b. The obtained isotherm exhibits a typical Type IV curve accompanied by a well-defined hysteresis loop, which is a characteristic feature of mesoporous materials.^67,68,71^ The existence of a hysteresis loop in the adsorption/desorption curve suggests the occurrence of capillary condensation within the pores of the nanocomposite structure. The specific surface area was determined using the BET equation with data from the linear portion of the BET plot obtained for P/Po values ranging from 0.05–0.30. The significant surface area of the nanocomposite is due to a homogeneous distribution of particles throughout the support, thus minimizing particle agglomeration and maximizing the density of reactive surface sites. BJH method was used to evaluate the pore size distribution by analyzing the desorption branch of the isotherm shown as Fig. 7(c).^68^ Results indicated that the majority of pores are mesopores, where diffusion of reactants can occur efficiently and increase the surface reactivity of the nanocomposite. The WFe_2_O_4_/rGO nanocomposite possesses a well-developed mesoporous structure with a relatively large specific surface area such as 75.76 m^2^ g^−1^ with pore size of 13.81 nm which is beneficial for applications in catalysis and adsorption.^67,68^

**Fig. 7:**
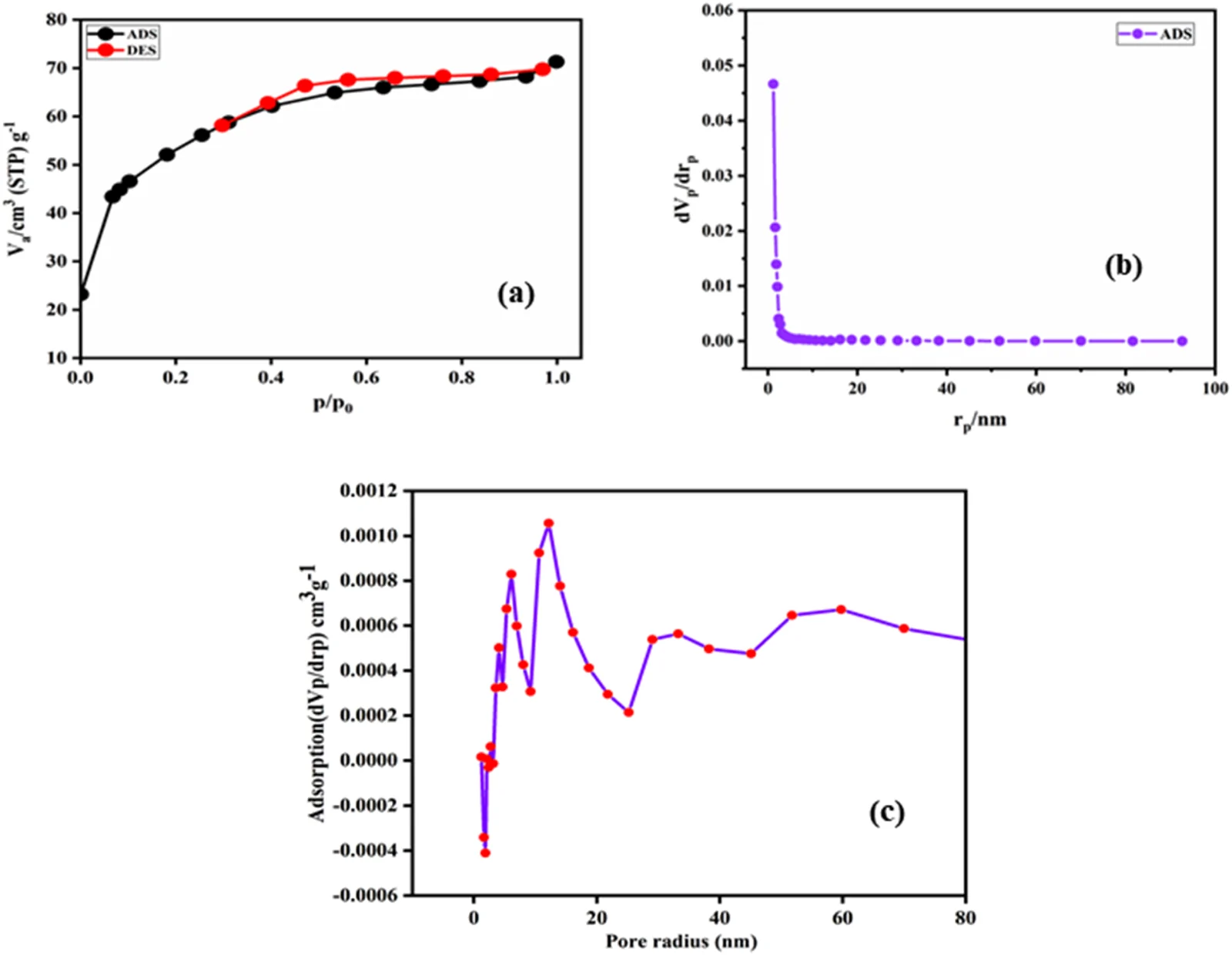
(a) N_2_ adsorption and desorption of WFe_2_O_4_/rGO; (b) BET surface area; (c) BJH pore size distribution.

The Raman spectrum of investigated samples was shown in Fig. 8. The spectrum compares WFe_2_O_4_ and its composite with reduced graphene oxide WFe_2_O_4_/rGO.^72–76^ The prominent peak in WFe_2_O_4_ (red line) near 718 cm^−1^ and 807 cm^−1^ indicates metal–oxygen stretching modes such as W–O and Fe–O. The peaks near 250–350 cm^−1^ represent Fe–O bending vibrations. In WFe_2_O_4_/rGO (black line) D band at 1353 cm^−1^ indicates structural defects and disorder in the carbon lattice, and the G band at 1587 cm^−1^ indicates vibrations in sp^2^ carbon atoms.^72–74^ The 2D band at nearly 2700 cm^−1^ indicates the second-order overtone of the D band of the graphene layers and crystalline quality.^73,75^ The ID/IG value near 0.89 indicates a moderately defective graphitic lattice.^74,76^ The presence of both the oxide peaks and carbon bands confirms successful synthesis of WFe_2_O_4_/rGO NPs.

**Fig. 8:**
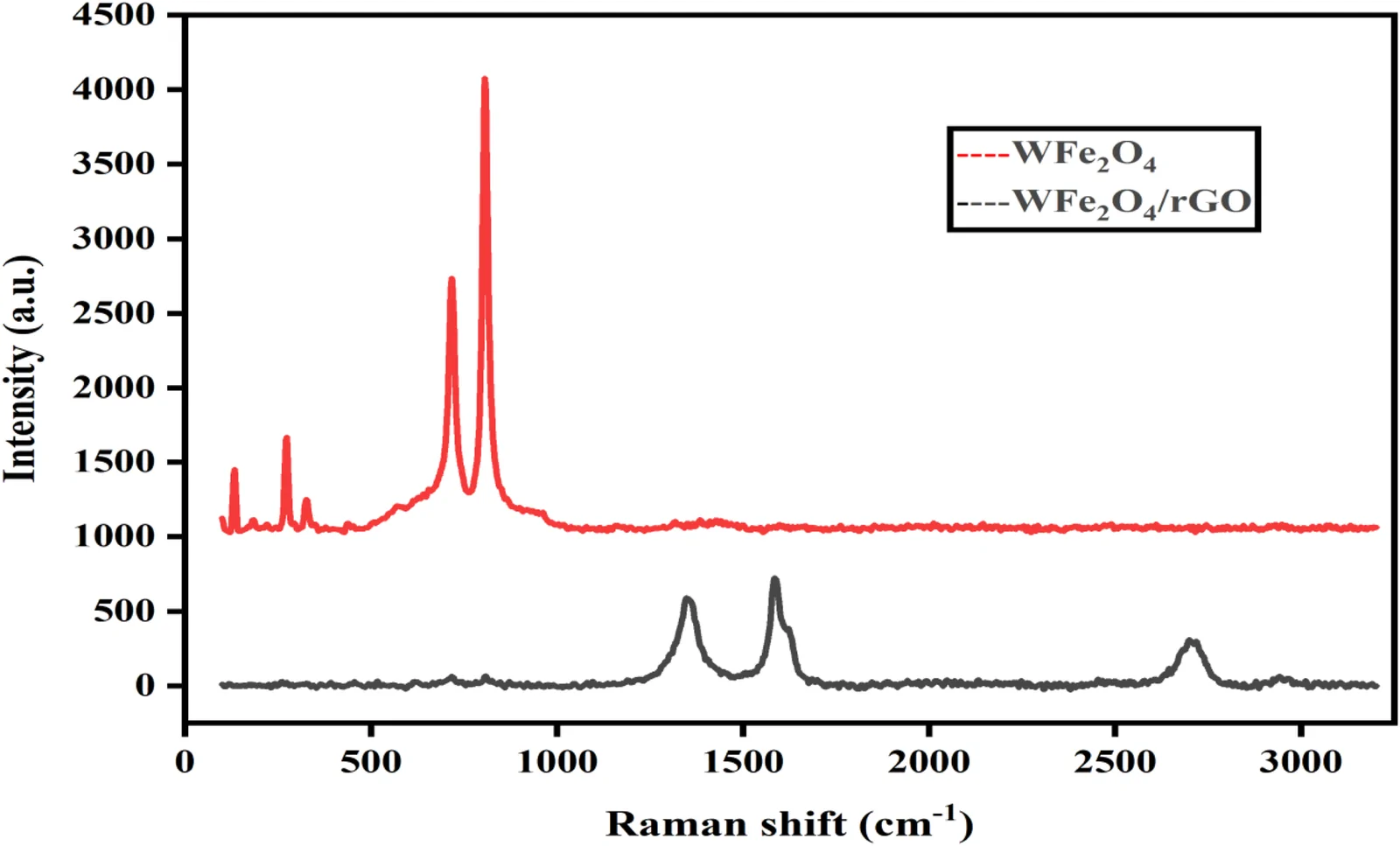
Raman spectra of WFe_2_O_4_ and WFe_2_O_4_/rGO nanocatalyst.

The thermogravimetric analysis of the synthesized WFe_2_O_4_/rGO NPs was carried out in the temperature range of 25–995 °C shown in Fig. 9 to evaluate its thermal stability. An initial weight loss of approximately 8.19% below 200 °C is attributed to the removal of physically adsorbed water molecules and volatile solvent species adsorbed on the surface. A gradual weight loss of about 5–6% between 200 and 800 °C corresponds to the decomposition of residual oxygen-containing functional groups present on the surface of rGO sheets.^5,47,48^ A pronounced weight loss observed between 850 and 950 °C is associated with the oxidation and degradation of the graphitic carbon framework of rGO. The nanocomposite retains nearly 70% of its original mass at 995 °C, indicating the high thermal stability of the WFe_2_O_4_/rGO phase. The results demonstrate that WFe_2_O_4_/rGO possesses excellent thermal stability and strong interfacial interaction between the ferrite nanoparticles and rGO matrix.

**Fig. 9:**
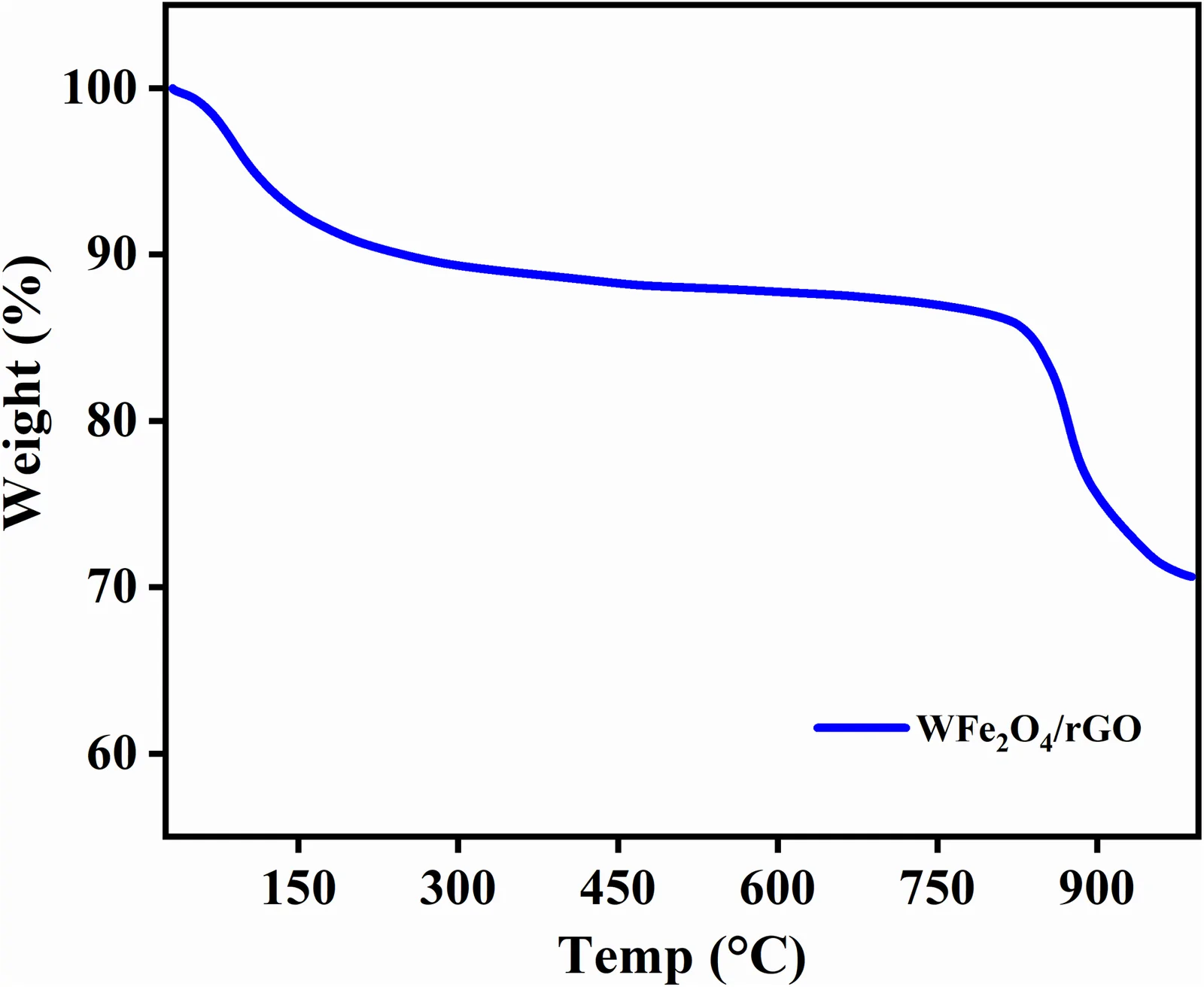
TGA analysis of WFe_2_O_4_/rGO nanocomposite.

The particle size analyzer and zeta potential tool were used to analyze the size and stability of the synthesized nanocomposite. The synthesized WFe_2_O_4_/rGO NPs are shown in Fig. 10(a and b), which demonstrates an average particle size of 98.0 nm and a zeta potential value of −40.2 mV. This indicates that the nanoparticles are well dispersed and colloidally stable.

**Fig. 10:**
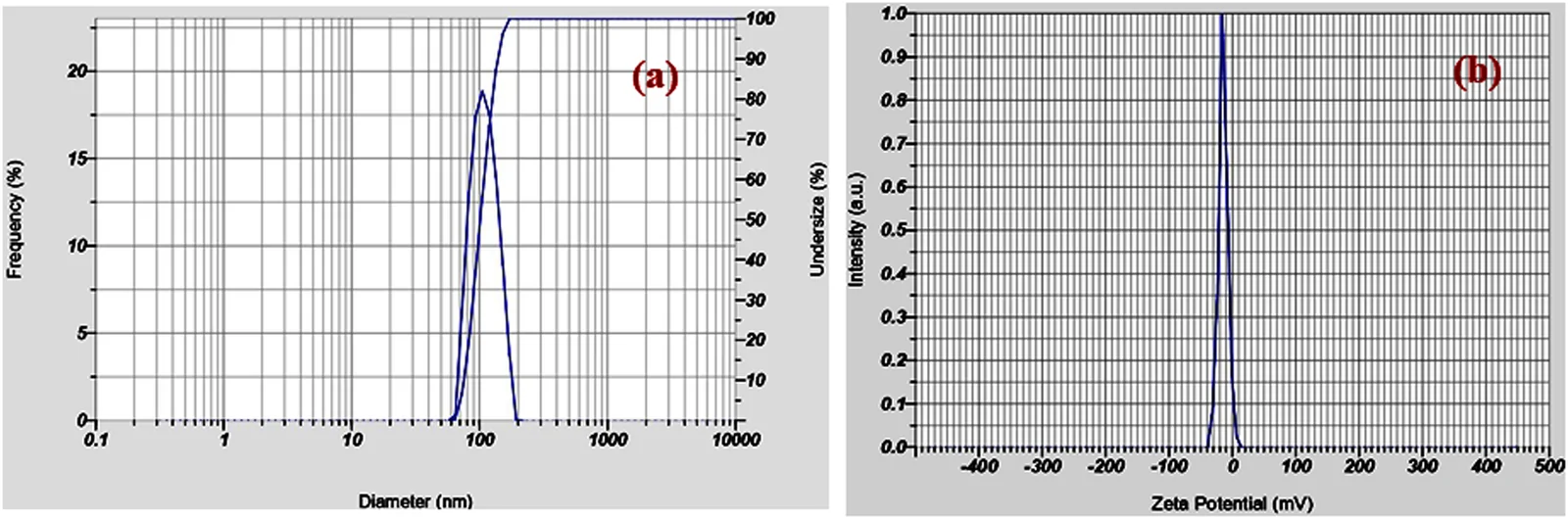
(a) Particle size analyzer and (b) zeta potential.

The XPS spectrum of WFe_2_O_4_/rGO composite shows Fig. 11a–f characteristic signals of W, Fe, O, and C. The W 4f, Fe 2p, and O 1s signals originate from the tungsten iron oxide structure, while the C 1s signal is associated mainly with the rGO component. The absence of prominent signals from other elements indicates the relatively good surface purity of the synthesized WFe_2_O_4_/rGO composite. In Fig. 11b shows high resolution spectrum of W 4f. These consist of well-defined spin orbit doublet located at 35.83 and 37.86 eV, the peaks are assigned to W 4f_7/2_ and W 4f_5/2_, respectively with a separation of 2.03 eV. The clear doublet and appropriate spin orbit separation confirm the presence of oxidized tungsten (W^6+^ species) in the WFe_2_O_4_ lattice. Although, in Fig. 11c shows the Fe 2p spectrum displays two principal peaks at 714.17 and 724.58 eV, corresponding to the Fe 2p_3/2_ and Fe 2p_1/2_ respectively. The energy difference between these two peaks is 10.41 eV, consistent with the spin orbit splitting associated with Fe 2p states. The Fe 2p indicates the presence of oxidized iron (Fe^2+^/Fe^3+^) species in the WFe_2_O_4_ composite. Furthermore, in Fig. 11d shows the C 1s spectrum exhibits a dominant peak at 285.53 eV. The C 1s component is primarily attributed to C–C/C=C bonds associated with the graphitic carbon framework of rGO. The relatively broad nature of the C 1s peak may arise from structural disorder, defects and residual oxygen-containing functional groups in rGO. In Fig. 11e shows the O 1s spectrum exhibits a dominant peak at 530.94 eV, which can be assigned predominantly to lattice oxygen (O^2−^), associated with W–O and Fe–O bonds in the WFe_2_O_4_ oxide framework.

**Fig. 11:**
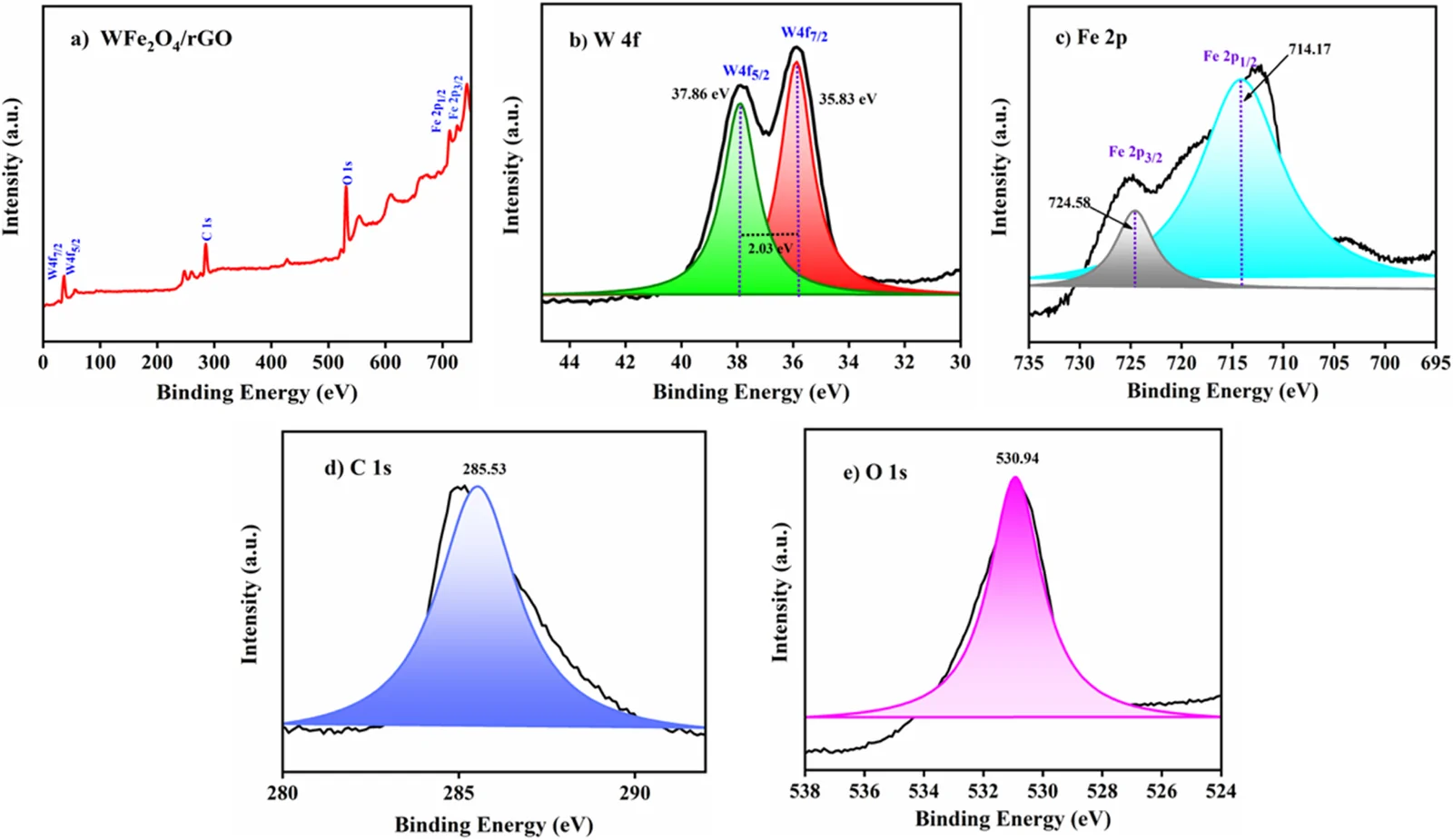
(a) XPS broad range survey spectrum, a high-resolution spectrum of WFe_2_O_4_/rGO (b) W 4f, (c) Fe 2p, (d) C 1s, (e) O 1s, of WFe_2_O_4_/rGO nanocomposite.

### 2.2. Catalytic application of WFe_2_O_4_/rGO nanocatalyst in the synthesis of thiadiazolo[2,3-*b*]quinazolin-6(7*H*)-one derivatives

We initiated our exploration by examining the reactivity of 4-nitrobenzaldehyde (T1) (1 mmol), 2-amino-5-phenyl-1,3,4-thiadiazole (S) (1 mmol), and dimedone (U) (1 mmol), which were selected as a model to optimize the reaction conditions, as shown in Table 1.^2,3,10,30,32,77^ To commence the investigation, initially, the model reaction was carried out in the absence of the catalyst (Table 1, entry 1); the desired product was obtained in trace yield even after 24 h at 80 °C under solvent-free conditions. When a catalytic amount of WFe_2_O_4_ (5 mg) was added in DMF and water, this led to a trace-to-moderate yield of the desired product (Table 1, entries 3 and 4).

**Table 1:** Optimization reaction condition of synthesis of thiadiazolo [2,3-*b*] quinazolin-6-(*7H*)-ones(V1–8)^a^

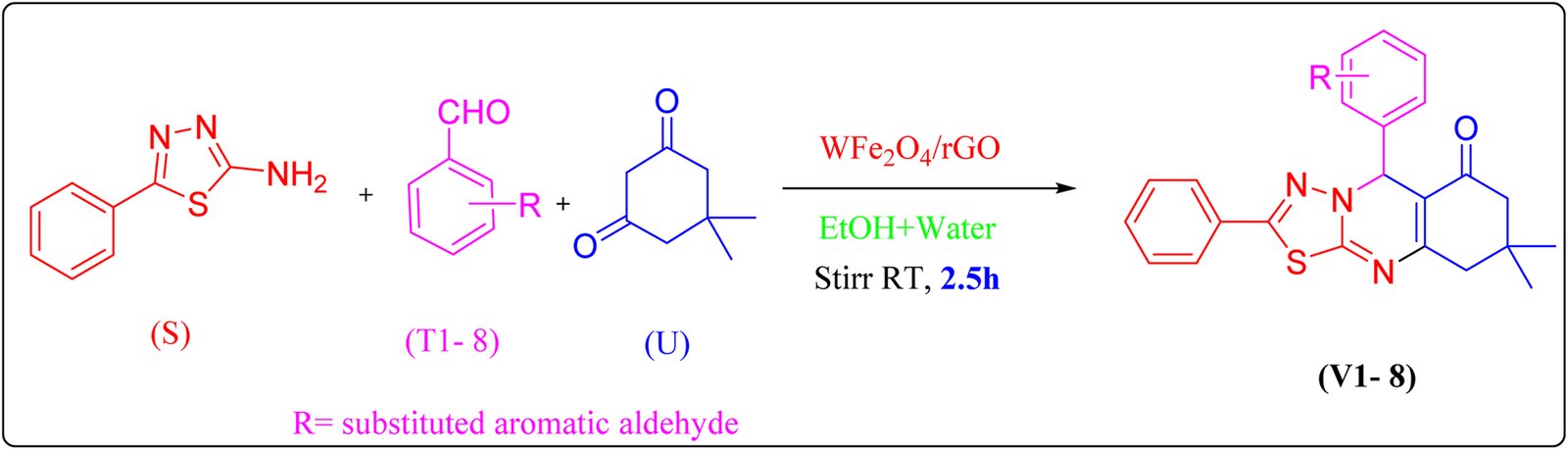					
Entry	Catalyst (mg)	Solvent	Temp.	Time (h)	Yield^b^ (%)
1	—	—	80 °C	24	Traces
2	GO (5 mg)	DMF	60 °C	24	25
3	WFe_2_O_4_ (5 mg)	DMF	60 °C	20	38
4	WFe_2_O_4_ (5 mg)	Water	60 °C	16	40
5	WFe_2_O_4_/GO (5 mg)	Water	R.T.	8	66
6	WFe_2_O_4_/rGO (5 mg)	Ethanol	R.T.	6	69
7	WFe_2_O_4_/rGO (5 mg)	Water–ethanol	R.T.	3	74
8	WFe_2_O_4_/rGO (10 mg)	Water–ethanol	R.T.	2.5	81
9	**WFe**_**2**_ **O**_**4**_ **/rGO (15 mg)**	**Water–ethanol**	**R.T.**	**2.5**	**89**
10	WFe_2_O_4_/rGO (20 mg)	Water–ethanol	R.T.	2.5	84
11	WFe_2_O_4_/rGO (25 mg)	Water–ethanol	R.T.	2.5	79

a Reaction condition: 2-amino-5-phenyl,1,3,4-thiadiazole (S) (1 mmol), dimedone (U) (1 mmol), and substituted aromatic aldehyde derivative (Ta-h) (1 mmol) were refluxed to room temp with 15 mg WFe_2_O_4_/rGO in an ethanol water solvent system.

b Isolated yields.

Next, the utility of the WFe_2_O_4_/rGO nanocatalyst for the same model reaction was evaluated using 5 mg of catalyst in various solvents or solvent mixtures, including water, ethanol and ethanol : water (1 : 1 v/v) system (Table 1, entries 5–8).^78–80^ From these results, ethanol:water was identified as the preferred solvent, giving the best product yield (Table 1, entry 7). It is also used for further catalytic screening studies. Interestingly, the reaction carried out in ethanol : water (1 : 1 v/v) with 15 mg of the WFe_2_O_4_/rGO nanocatalyst also proceeded efficiently, yielding 89% of the desired product (Table 1, entry 9). The same model reaction was repeated using 20 mg and 25 mg of catalyst in ethanol : water (1 : 1 v/v), but no significant change in reaction time or product yield was observed (Table 1, entries 10–11).^1,2,4^ However, no significant improvement in product yield was observed in any of the other cases, justifying the utility of 15 mg of WFe_2_O_4_/rGO nanocatalyst for the efficient synthesis of thiadiazolo[2,3-*b*]quinazolin-6(7*H*)-ones derivative V1 (89%) in ethanol : water (1 : 1 v/v) under room temperature conditions (Table 1, entry 9).

To extend the scope of this newly synthesized WFe_2_O_4_/rGO catalyst, various thiadiazolo[2,3-*b*]quinazolin-6(7*H*)-one derivatives possessing electron-withdrawing and electron-donating groups were synthesized (Table 2, entries V1–V8).^30–32^ Notably, thiadiazolo[2,3-*b*]quinazolin-6(7*H*)-ones derivatives having electron-withdrawing (–NO_2_ and –Cl) groups afforded the desired products in excellent yield within 2.5 h. On the other hand, electron-donating (–OH, –CH_3_, –N(CH_3_)_2_, –OCH_3_) groups afforded good yields within 2.5 h. The present protocol not only provides an excellent yield of products in shorter reaction times but also offers benefits in terms of catalyst recyclability.

**Table 2:** Synthesis of thiadiazolo [2,3-*b*] quinazolin-6-ones (V1–8) using WFe_2_O_4_/rGO nanocatalyst^a^

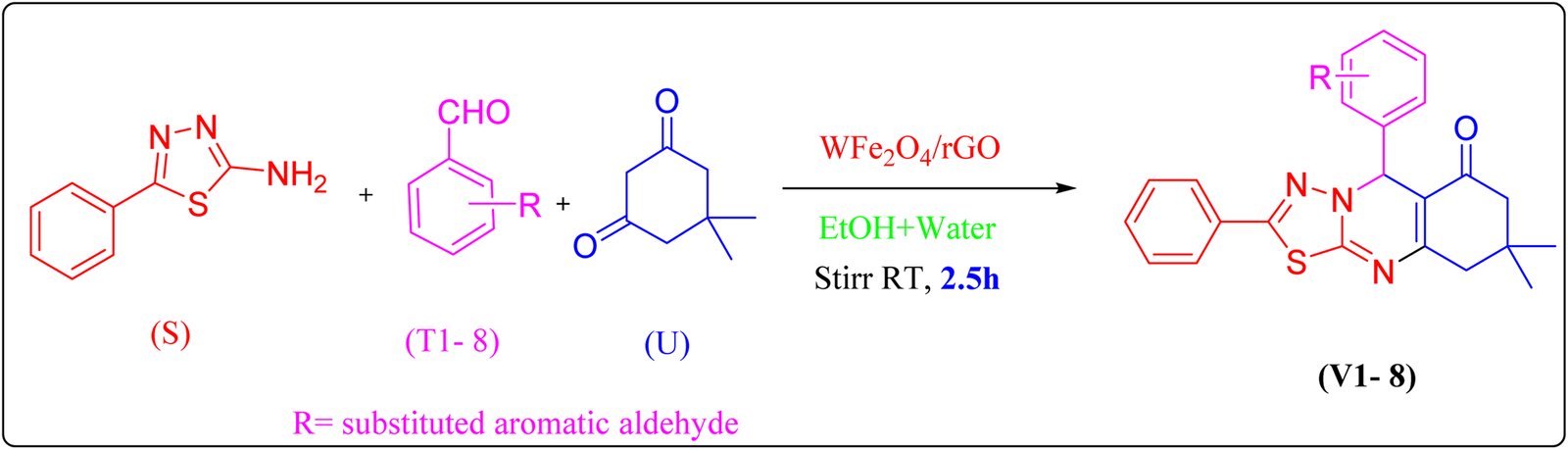						
Entry	Aldehyde (T1–8)	Product (V1–8)	Time (h)	Yield^b^ (%)	TON/TOF (H^−1^)	Melting point (°C)
1	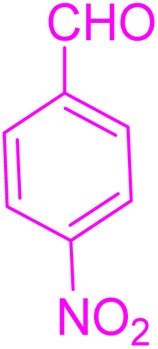	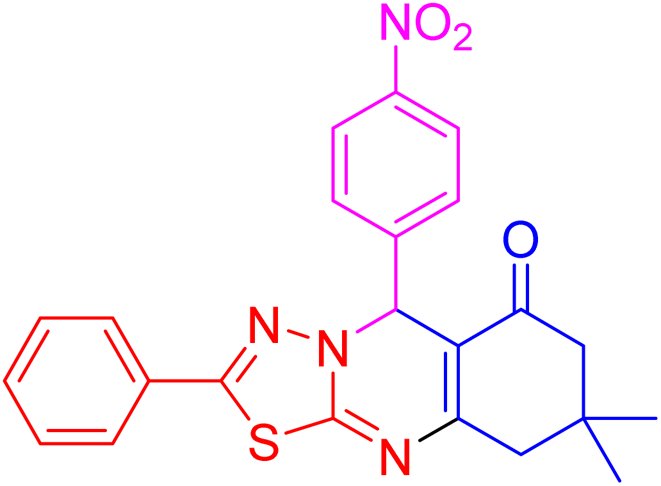	2.5	89	2133/853	205
2	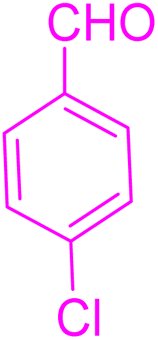	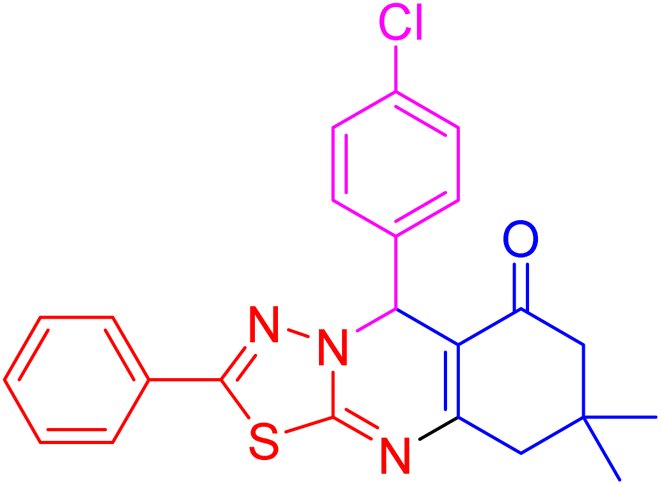	2.5	86	2061/824	213
3	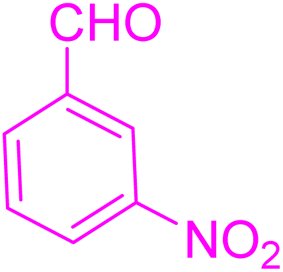	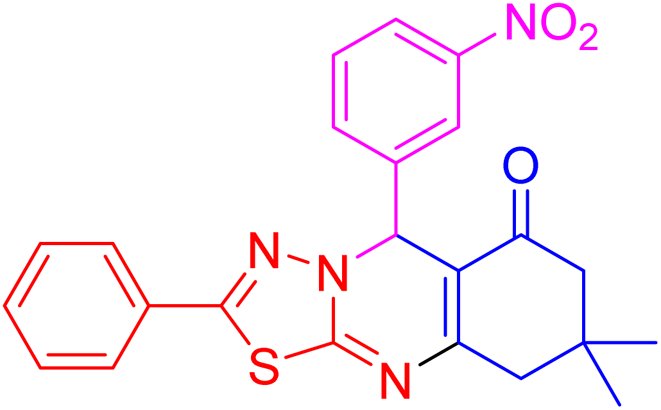	2.5	87	2085/834	209
4	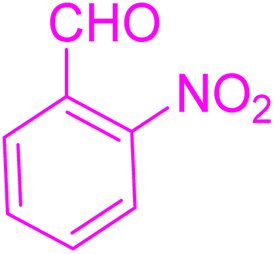	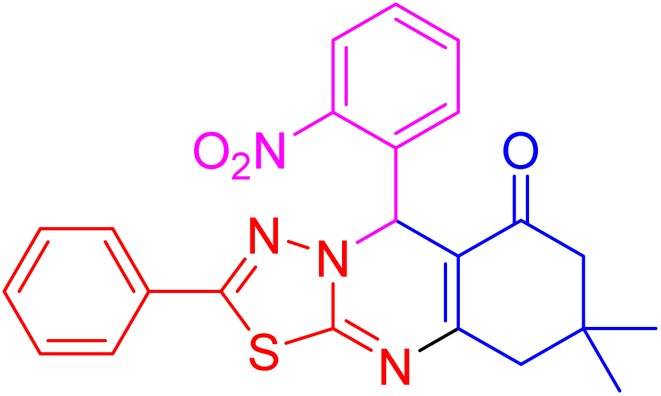	2.5	86	2061/824	216
5	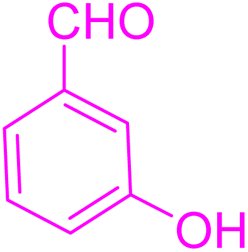	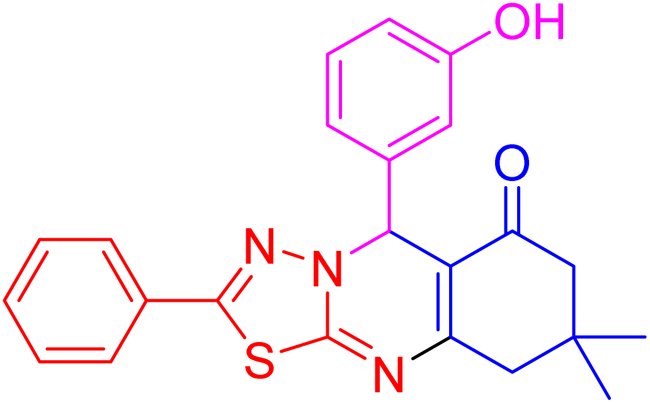	2.5	80	1917/766	218
6	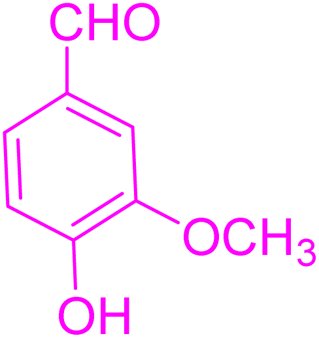	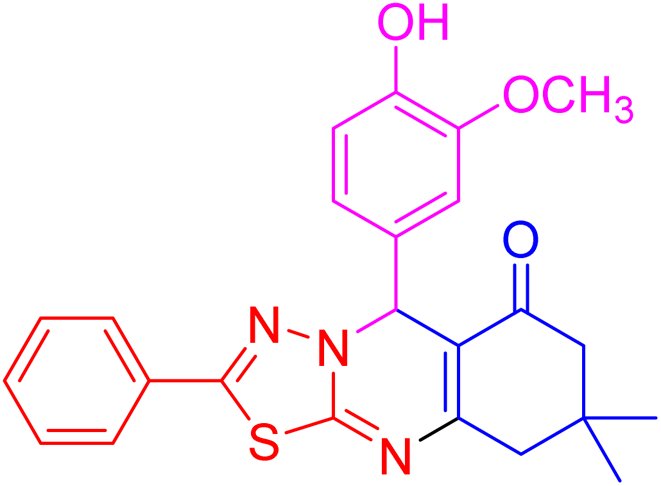	2.5	82	1965/786	217
7	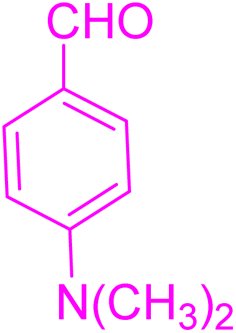	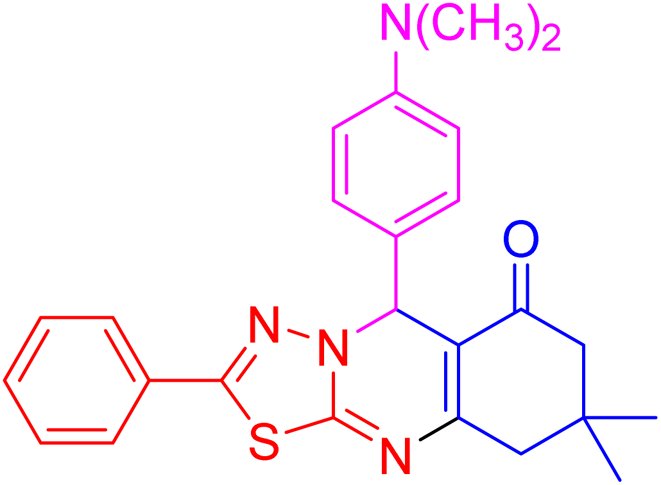	2.5	81	1941/776	205
8	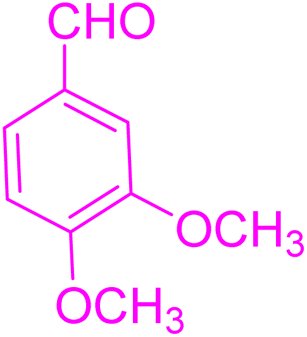	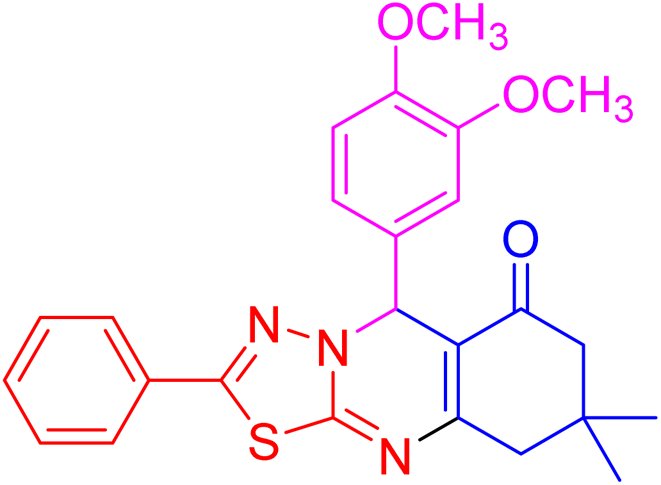	2.5	80	1917/766	193

a Reaction condition: 2-amino-5-phenyl,1,3,4-thiadiazole (S) (1 mmol), dimedone (U) (1 mmol), and substituted aromatic aldehyde derivative (Ta-h) (1 mmol) were refluxed to room temp with 15 mg WFe_2_O_4_/rGO in an ethanol water solvent system.

b Isolated yields.

Previously, there were few reports available in the literature based on the synthesis of thiadiazolo[2,3-*b*]quinazolin-6(7*H*)-ones motifs. Notably, using WFe_2_O_4_/rGO as a heterogeneous catalyst in this protocol has demonstrated significant advantages over previously reported methods.^2,3,10^ To our delight, the key benefits of this approach include operational simplicity, the use of a green catalyst in an equimolar mixture of ethanol : water (1 : 1 v/v), high atom economy, effortless magnetic recoverability and reusability, and easy product isolation and purification without the need for a column chromatographic procedure (Table 3).

**Table 3:** Comparison of WFe_2_O_4_/rGO heterogeneous catalyst for the synthesis of thiadiazolo [2,3-*b*] quinazolin-6-ones derivatives (V1–8) with other catalysts reported in literature^a^

Entry	Catalyst	Solvent	Temp. (°C)	Time (h)	Yield (%)^b^
1	Bu_4_NHSO_4_	Ethanol + water	60	2	92
2	Molecular I_2_	CH_3_CN	80	3	80
3	Diphenylhydramine-CoCl_2_·6H_2_O deep eutectic solvent	Solvent free	120	2	90
4	**Magnetic WFe**_**2**_ **O**_**4**_ **/rGO**	**Ethanol + Water**	**R.T.**	**2.5**	**89**

a Reaction condition: 2-amino-5-phenyl,1,3,4-thiadiazole (S) (1 mmol), dimedone (U) (1 mmol), and substituted aromatic aldehyde derivative (Ta-h) (1 mmol) were refluxed to room temp with 15 mg WFe_2_O_4_/rGO in an ethanol water solvent system.

b Isolated yields.

To evaluate the efficiency of the newly developed magnetic WFe_2_O_4_/rGO heterogeneous catalyst, its performance was compared against various catalytic systems reported in the literature for the synthesis thiadiazolo [2,3-*b*] quinazolin-6-ones derivatives. Catalyst such as Bu_4_NHSO_4_ (entry 1), molecular I_2_ (entry 2) and deep eutectic solvent system (entry 3) required elevated temp ranging from 60 °C to 120 °C to achieve optimal yields. In contrast, the magnetic WFe_2_O_4_/rGO catalyst (entry 4) successfully facilitated the reaction at room temp. (R.T.). It provided an excellent yield of 89% within a short reaction time of 2.5 hours. Furthermore, the green solvent mixture highlights the environmentally benign nature of this protocol compared to hazardous media like CH_3_CN. Consequently, the magnetic WFe_2_O_4_/rGO composite serves as a highly sustainable, energy efficient and competitive alternative to existing methods.

#### 2.2.1. Spectroscopic data of thiadiazolo[2,3-*b*]quinazolin 6-one synthesized compounds (V1–8)

##### 2.2.1.1 5-(4-Nitrophenyl)8,9-dihydro-8,8-dimethyl-2-phenyl-5*H*-[1,3,4]thiadiazolo[2,3-*b*]quinazolin-6(7*H*)-one (V-1)

M.P. 205 °C, yield 89%, M.F. C_23_H_20_O_3_N_4_S; IR (KBr/cm^−1^): 3374, 3088, 2958, 1721, 1516, 1382, 1232; ^1^H NMR (400 MHz, DMSO-d_6_): *δ* (ppm) 7.5–7.8 (m, 4H), 7.0–7.3 (m, 5H), 6.2 (s, 1H, –CH), 2.4 (s, 2H, –CH_2_), 2.1 (s, 2H, –CH_2_), 1.1 (s, 3H, –CH_3_), 0.8 (s, 3H, –CH_3_); ^13^C NMR (100 MHz, DMSO-d_6_): *δ* (ppm) 204.32, 196.06, 187.25, 168.93, 157.30, 129.63, 124.15, 113.89, 109.78, 56.59, 50.78, 28.26, 29.72, 26.49; EI-MS (*m*/*z*: RA%): [M + 4] 436 (*m*/*z*: 100%).

##### 2.2.1.2 5-(4-Chlorophenyl)8,9-dihydro-8,8-dimethyl-2-phenyl-5*H*-[1,3,4]thiadiazolo[2,3-*b*] quinazolin-6(*7H*)-one (V-2)

M.P. 213 °C, yield 86%, M.F. C_23_H_20_ON_3_SCl; IR (KBr/cm^−1^): 3374, 3088, 2958, 1721, 1516, 1382, 1232; ^1^H NMR (400 MHz, DMSO-d_6_): *δ* (ppm) 7.9–7.3 (m, 4H), 7–7.3 (m, 5H), 5.7 (s, 1H, –CH), 2.2 (s, 2H, –CH_2_), 2.5 (s, 2H, –CH_2_), 1.1 (s, 3H, –CH_3_), 0.9 (s, 3H, –CH_3_); ^13^C NMR (100 MHz, DMSO-d_6_): *δ* (ppm) 197.26, 181.95, 168.93, 140.29, 131.36, 133.32, 129.64, 126.08, 112.42, 56.58, 49.50, 40.40, 31.40, 28.26; EI-MS (*m*/*z*: RA%): 421 (*m*/*z*: 100%).

##### 2.2.1.3 5-(3-Nitrophenyl)8,9-dihydro-8,8-dimethyl-2-phenyl-5*H*-[1,3,4]thiadiazolo[2,3-*b*] quinazolin-6(7*H*)-one (V-3)

M.P. 209 °C, yield 87%, M.F. C_23_H_20_O_3_N_4_S; IR (KBr/cm^−1^): 3264, 3088, 3061, 1632, 1593, 1511, 1376, 1249; ^1^H NMR (400 MHz, DMSO-d_6_): *δ* (ppm) 7.0–7.4 (m, 5H) 7.5–8.2 (m, 4H), 6.04 (s, 1H, –CH), 2.51 (s, 2H, –CH_2_), 2.11 (s, 2H, –CH_2_), 1.15 (s, 3H, –CH_3_), 0.97 (s, 3H, –CH_3_) ; ^13^C NMR (100 MHz, DMSO-d_6_): *δ* (ppm) 190.40, 184.50, 172.37, 169.17, 143.72, 150.97, 128.13, 124.73, 117.12, 109.69, 41.79, 26.48, 23.51; EI-MS (*m*/*z*: RA%): [M + 4] 436 (*m*/*z*: 100%).

##### 2.2.1.4 5-(2-Nitrophenyl)8,9-dihydro-8,8-dimethyl-2-phenyl-5*H*-[1,3,4]thiadiazolo[2,3-*b*] quinazolin −6(7*H*)-one (V-4)

M.P. 216 °C, yield 86%, M.F. C_23_H_20_O_3_N_4_S; IR (KBr/cm^−1^): 3371, 3280, 3088, 2959, 1739, 1518, 1383, 1232; ^1^H NMR (400 MHz, DMSO-d_6_): *δ* (ppm) 7.2–8.1 (m, 4H), 7.0–7.2 (m, 5H), 6.34 (s, 1H, –CH), 2.27 (s, 2H, –CH_2_), 2.3 (s, 2H, –CH_2_), 1.18 (s, 3H, –CH_3_), 0.95 (s, 3H, –CH_3_); ^13^C NMR (100 MHz, DMSO-d_6_): *δ* (ppm) 204.55, 168.94, 157.09, 132.72, 131.42, 113.83, 101.50, 56.61, 42.79, 46.97, 33.14, 28.27, 26.54. ppm; EI-MS (*m*/*z*: RA%): [M + 4] 436 (*m*/*z*: 100%).

##### 2.2.1.5 5-(3-Hydroxylphenyl)8,9-dihydro-8,8-dimethyl-2-phenyl-5*H*-[1,3,4]thiadiazolo[2,3-*b*] quinazolin-6(7*H*)-one (V-5)

M.P. 218 °C, yield 80%, M.F. C_22_H_21_O_2_N_3_S; IR (KBr/cm^−1^): 3272, 3072, 2959, 1592, 1513, 1468, 1139 cm^−1^. ^1^H NMR (400 MHz, DMSO-d_6_): *δ* (ppm) 7.0–7.81 (m, 4H), 7.00–6.60 (m, 5H), 6.43 (s, 1H, –CH), 8.9 (s, –OH), 2.70 (s, 2H, –CH_2_), 2.27 (s, 2H, –CH_2_), 1.09 (s, 3H, –CH_3_), 1.01 (s, 3H, –CH_3_); ^13^C NMR (100 MHz, DMSO-d_6_): *δ* (ppm) 168.94, 157.35, 131.35, 129.63, 126.57, 115.25, 78.55, 47.18, 31.34; EI-MS (*m*/*z*: RA%): 391 (*m*/*z*: 100%).

##### 2.2.1.6 5-(4-Hydroxy-3-methoxyphenyl)8,9-dihydro-8,8-dimethyl-2-phenyl-5*H*-[1,3,4]thiadiazolo[2,3-*b*]quinazolin-6(7*H*)-one (V-6)

M.P. 217 °C, yield 82%, M.F. C_24_H_23_O_3_N_3_S; IR (KBr/cm^−1^): 3269, 3058, 2957, 1632, 1588, 1512, 1467, 1238 cm^−1^ 2957 ^1^H NMR (400 MHz, DMSO-d_6_): *δ* (ppm) 8.11–8.33 (m, 5H), 6.51–7.5 (m, 3H), 8.54 (s, 1H, –OH), 5.60 (s, 1H, –CH), 3.83 (s, 3H, –OCH_3_), 2.29 (s, 2H, –CH_2_), 2.13 (s, 2H, –CH_2_), 1.20 (s, 3H, –CH_3_), 0.96 (s, 3H, –CH_3_); ^13^C NMR (100 MHz, DMSO-d_6_): *δ* (ppm) 189.06, 168.90, 144.33, 147.34, 126.57, 131.39, 129.03, 129.59, 115.15, 115.57, 110.81, 55.60, 46.60, 46.77, 31.80, 31.31; EI-MS (*m*/*z*: RA%): [M + 4] 437 (*m*/*z*: 100%).

##### 2.2.1.7 5-(4-*N*,*N*-Dimethylphenyl)8,9-dihydro-8,8-dimethyl-2-phenyl-5*H*-[1,3,4]thiadiazolo[2,3-*b*]quinazolin-6(7*H*)-one (V-7)

M.P. 205 °C, yield 81%, M.F. C_24_H_26_N_4_OS; IR (KBr/cm^−1^): 3088, 2958, 1721, 1530, 1362, 1232; ^1^H NMR (400 MHz, DMSO-d_6_): *δ* (ppm) 7.20–7.30 (m, 4H), 7-7.50 (m, 5H), 6.11 (s, 1H, –CH), 2.35 (s, 6H), 2.25 (s, 2H, –CH_2_), 2.08 (s, 2H, –CH_2_), 1.17 (s, 3H, –CH_3_), 0.96 (s, 3H, –CH_3_); ^13^C NMR (100 MHz, DMSO-d_6_): *δ* (ppm) 195.26, 140.29, 131.36, 129.64, 126.08, 112.42, 55.55, 45.40, 40.40, 31.40, 28.26; EI-MS (*m*/*z*: RA%): 430 (*m*/*z*: 100%).

##### 2.2.1.8 5-(3,4-Dimethoxyphenyl)8,9-dihydro-8,8-dimethyl-2-phenyl-5*H*-[1,3,4]thiadiazolo[2,3-*b*]quinazolin-6(7*H*)-one (V-8)

M.P. 193 °C, yield 80%, M.F. C_25_H_25_N_3_O_3_S; IR-3058, 2957, 1632,1467, 1125, 1238 cm^−1 1^H NMR (400 MHz, DMSO-d_6_): *δ* (ppm) 7.54–7.26 (m, 5H), 7-6.5 (m, 3H), 6.35 (s, 1H, –CH), 3.73 (s, 3H, –OCH_3_), 3.83 (s, 3H, –OCH_3_), 2.19 (s, 2H, –CH_2_), 2.13 (s, 2H, –CH_2_), 1.20 (s, 3H, –CH_3_), 0.83 (s, 3H, –CH_3_); ^13^C NMR (100 MHz, DMSO-d_6_): *δ* (ppm) 189.06, 168.90, 144.33, 147.34, 126.57, 131.39, 129.59, 115.15, 55.60, 46.60, 46.77, 31.80, 31.31; EI-MS (*m*/*z*: RA%): 446 (*m*/*z*: 100%).

### 2.3. Computational details: density functional theory

#### 2.3.1. Optimized molecular structure

The thiadiazolo quinazolineone derivatives (V1, V2, V5, and V7) were optimized using the DFT/B3LYP (Becke three-parameter Lee–Yang–Parr) hybrid functional method^40,43,81,82^ with the 6-311G++(d,p) basis set gas phase using the Gaussian 16 package and the Gauss-View 6.0 molecule visualization tool.

The calculated geometrical parameters, that is, bond lengths and bond angles, are given in Tables S2–S5 (SI). This indicates that the bond lengths and bond angles of the five rings are consistent, with variation only at the substituted benzene positions.^39,41^Fig. 12. Optimization structure of molecule with labelling: (a) V1; (b) V2; (c) V5; (d) V7.

**Fig. 12:**
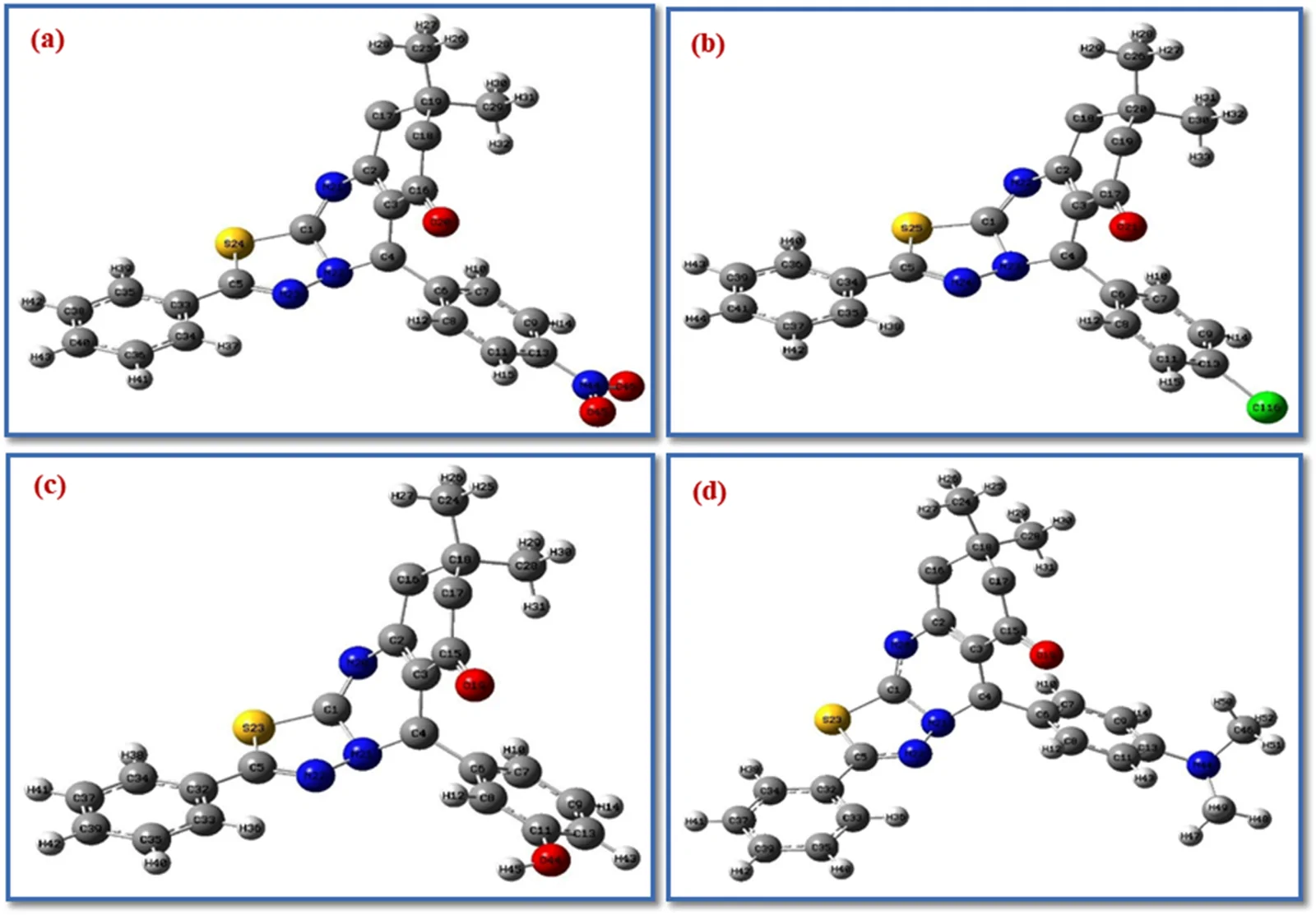
Optimization structure of molecule with labelling (a) V1; (b) V2; (c) V5; (d) V7.

#### 2.3.2. Mulliken atomic charges

Mulliken atomic charges of the thiadiazoloquinazolineone derivatives (V1, V2, V5, and V7) were calculated by DFT at the B3LYP level using the 6-311++G(d,p) basis set in the gas phase.^40,83–85^ The Mulliken atomic charge of carbon (C) and nitrogen (N) atoms is either positive or negative, indicating the distribution of electron density around the atoms. All hydrogen atoms possess a positive charge value. Oxygen and sulfur have only negative charge values. C16 (V1), C17 (V2), C15 (V5), and C15 (V7) have higher negative charge values of −1.133554, −1.218185, −1.125230, and −1.07432, respectively, indicating that the carbon atom is linked to an electronegative atom, *i.e.*, the carbonyl carbon 84. In V1, H14 (0.257452) and H15 (0.257343) have larger positive values than other hydrogen atoms, as they are ortho to the –NO_2_ group. However, this is not found in V2, V5, or V7. The –OH group causes H45 (0.2586) to have larger positive values in V5 (Fig. 13a–d).

**Fig. 13:**
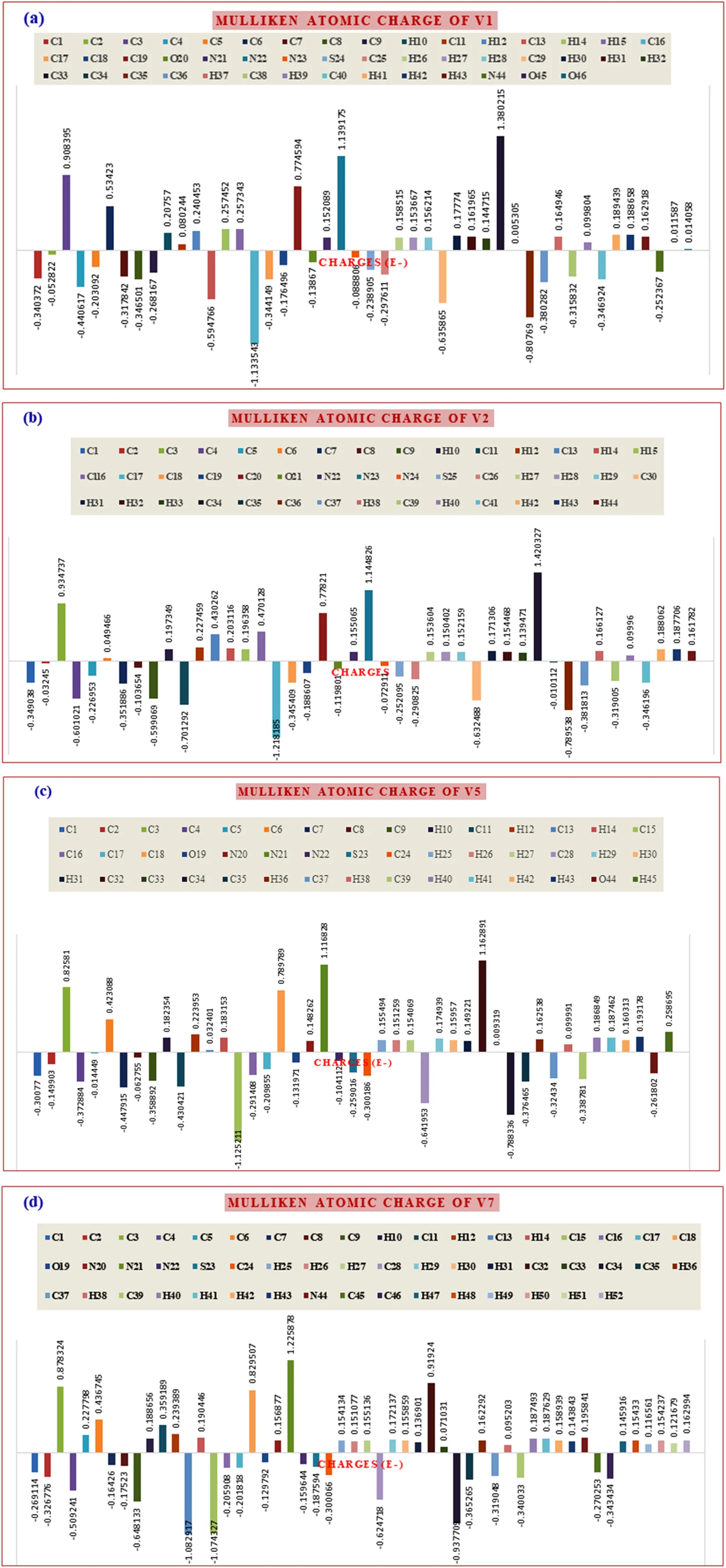
(a) Mulliken atomic charge values of V1. (b) Mulliken atomic charge values of V2. (c) Mulliken atomic charge values of V5. (d) Mulliken atomic charge values of V7.

#### 2.3.3. Frontier molecular orbital study

FMO analysis was carried out to explain the electronic properties and charge arrangement in the synthesized thiadiazoloquinazolineone derivatives.^39,41,86^ It is possible to isolate electron-donating and accepting zones, which are important for the reactivity assessment and prediction of where interactions can take place, by assessing frontier molecular orbitals. The HOMO energy and LUMO energy, and their spatial configurations were evaluated in derivatives (V1, V2, V5, and V7) and are shown in Table 4. In quantum chemistry, the energy difference between HOMO and LUMO is a strong marker of a molecule's tendency to be involved in a chemical reaction. A smaller energy gap is typically a characteristic of chemical softness and increased polarizability, which are favourable to electron transfer and reactivity, particularly in delocalized π-electron systems. On the other hand, a high energy gap implies chemical hardness and better electronic stability.^39,42,87^ The Δ*E* values obtained from V2 and V7 were 2.0645 and 2.2343 eV, respectively, indicating increased electronic softness in the molecules. On the contrary, compounds V1 and V5 exhibited higher energy gaps, 2.6313 and 2.6038 eV, respectively, which indicates less reactivity and a less mobile electronic structure. The smaller HOMO–LUMO energy gap of V2 (2.0645) indicates that it will be more electrically responsive and more reactive. These results from the frontier orbital distribution give us an insight into the electronic nature of these compounds and allow us to locate reactive sites Fig. 14.

**Table 4:** Electronic parameters of synthesized compounds^a^

Entry	*E*_total_ (a.u.)	*E*_HOMO_ (eV)	*E*_LUMO_ (eV)	Δ*E* (eV)	*I* (eV)	*A* (eV)
V1	−1728.2220	−6.4520	−3.8206	2.6313	6.4520	3.8206
V2	−1983.2747	−5.9573	−3.8928	2.0645	5.9573	3.8928
V5	−1598.9056	−6.1515	−3.5477	2.6038	6.1515	3.5477
V7	−1657.6446	−5.9271	−3.6928	2.2343	5.9271	3.6928

a *I* = −*E*_HOMO_ & *A* = −*E*_LUMO_.

**Fig. 14:**
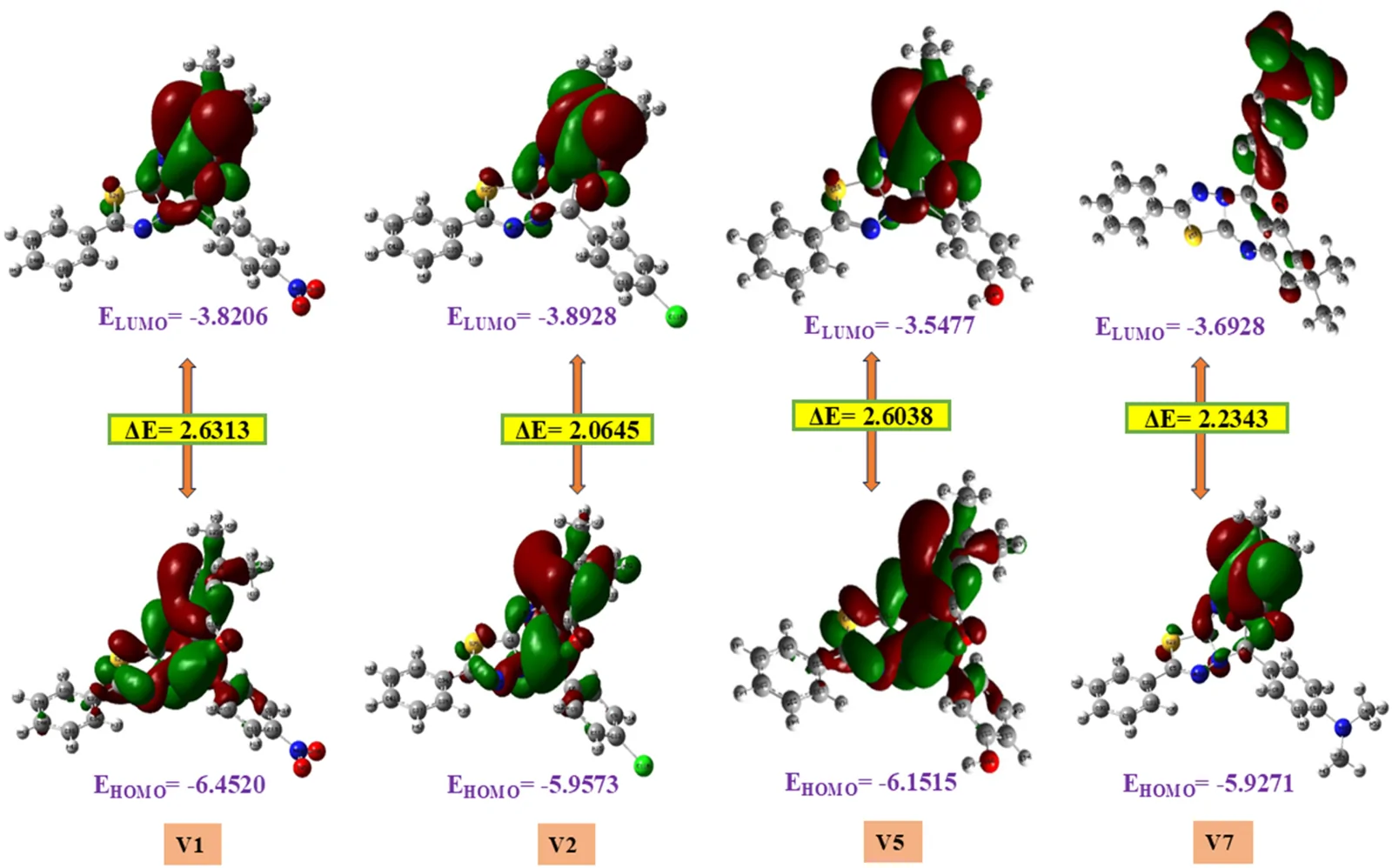
Frontier molecular orbit (HOMO–LUMO) diagrams of V1, V2, V5 & V7 by DFT at the B3LYP level using the 6-311++G(d,p) basis set.

Compounds V1, V2, V5, and V7 were selected for DFT calculation. V1, V2, and V5 were included because they showed the most favourable binding free energies among the docked compounds (V1–V6) in the molecular docking study, while V7 was not part of the docking set and was instead included as a representative electron-rich derivative for comparison of its electronic structure with the docked compounds. Important descriptors such as ionization potential (*I*) and electron affinity (*A*) were analysed from the data.^39,42,87^ From the data, compounds V2 and V7 show inferior ionization potentials; therefore, the molecules are more likely to lose electrons and consequently are more easily ionisable than V1 and V5. By contrast, compound V1 has significantly greater electron affinity, showing how it has a greater propensity to attract an electron relative to compounds V1 and V5 shown in Fig. 14.

#### 2.3.4. Global descriptors study

The Global Quantum Chemical Descriptors for the synthesized compounds V1, V2, V5, and V7 have been summarized within Table 5.^39,42,87^ These include Electronegativity (*χ*) which describes an atom's or molecule's “ability” to attract electron density. This also describes an atom/molecule's “chemical hardness” (*η*), which can be described as how easily it can lose or gain electrons. The “softness” (*σ*) is simply the inverse of the “hardness”. Other descriptors used were the “electrophilicity index” (*ω*) which helps predict a molecule's propensity to accept electrons. Also included was the “chemical potential” which has no direct units and is related to the chemical hardness. Lastly, another important descriptor listed was the “maximum electronic charge transfer”, this indicates the amount of electron density one molecule may pass onto another. All these descriptors help explain both molecular reactivity and stability.^39,41,42^

**Table 5:** Quantum chemical parameters^a^

Entry	*χ* (eV)	*η* (eV)	*σ* (eV)	*ω* (eV)	Pi (eV)	Δ*N*_max_
V1	5.1363	0.6313	1.5840	20.8943	−5.1363	8.1359
V2	4.9250	1.0322	0.9687	11.7488	−4.9250	4.7711
V5	4.8496	1.3019	0.7681	9.0324	−4.8496	3.7250
V7	4.8099	1.1171	0.8951	10.3549	−4.8099	4.3057

a *χ* = *I* + *A*/2; *η* = *I* − *A*/2; *σ* = 1/*η*; *ω* = Pi/2*n* Pi = −*χ*; Δ*N*_max_ = −Pi/*η*.

Among the synthesized compounds V1 was found to have the greatest “Electronegativity” (5.1363 eV), “Softness” (1.5840 eV), “Electrophilicity Index” (20.8943 eV), and “Maximum Electronic Charge Transfer” (Δ*N*_max_; 8.1359), all of which show a high degree of electron accepting ability, greater chemical reactivity, and higher charge transfer potential than the other three compounds studied. On the other hand, V5 had the largest “Chemical Hardness” (1.3019 eV), and therefore, shows increased molecular stability compared to the other compounds.

#### 2.3.5. Molecular electrostatic potential

The molecular electrostatic potential was calculated to identify the reactive sites of thiadiazoloquinazolineone derivatives (V1, V2, V5, and V7) at the B3LYP/6-311++G(d,p) level in the gas phase.^88–92^ The MEP surfaces (Table 5), show that the carbonyl oxygen atoms possess the most negative electrostatic potential (red), indicating favorable sites for electrophilic attack. In V1, the (–NO_2_) substituents also exhibit negative electrostatic potential (yellow), whereas the remaining substituents are located in regions of positive electrostatic potential (light blue). The green regions correspond to nearly neutral electrostatic potential. The contour maps further reveal that regions with denser contour lines possess stronger electrostatic fields, confirming the electron-rich nature of the carbonyl oxygen and its role as the primary reactive site (Table 6).

**Table 6:** Molecular electrostatic potential surface and contour

Entry	MEP surface	MEP counter
V1	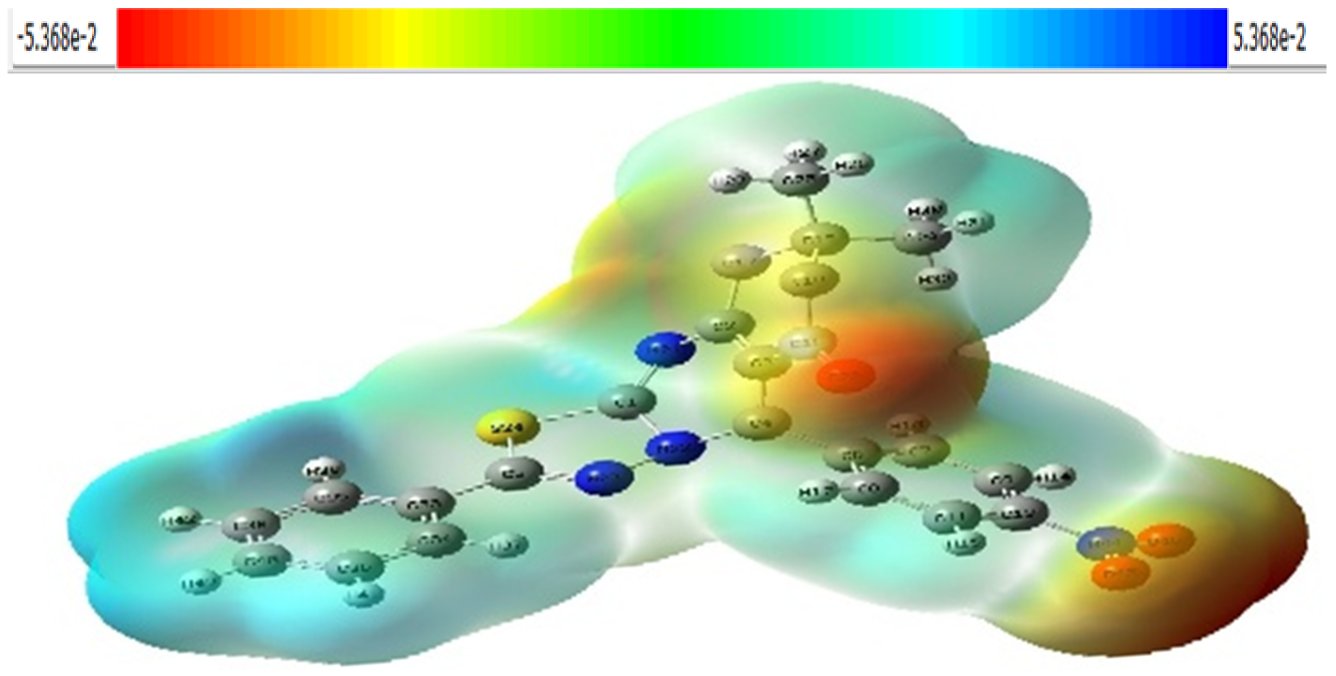	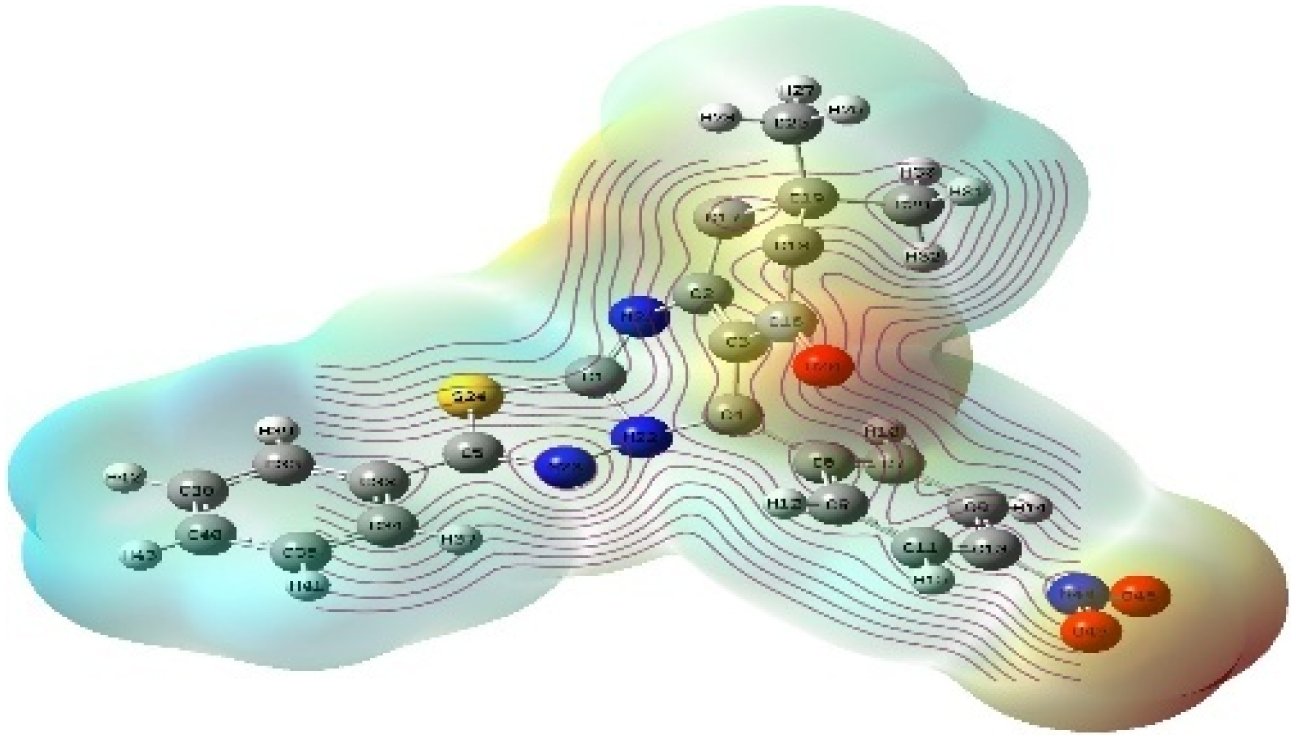
V2	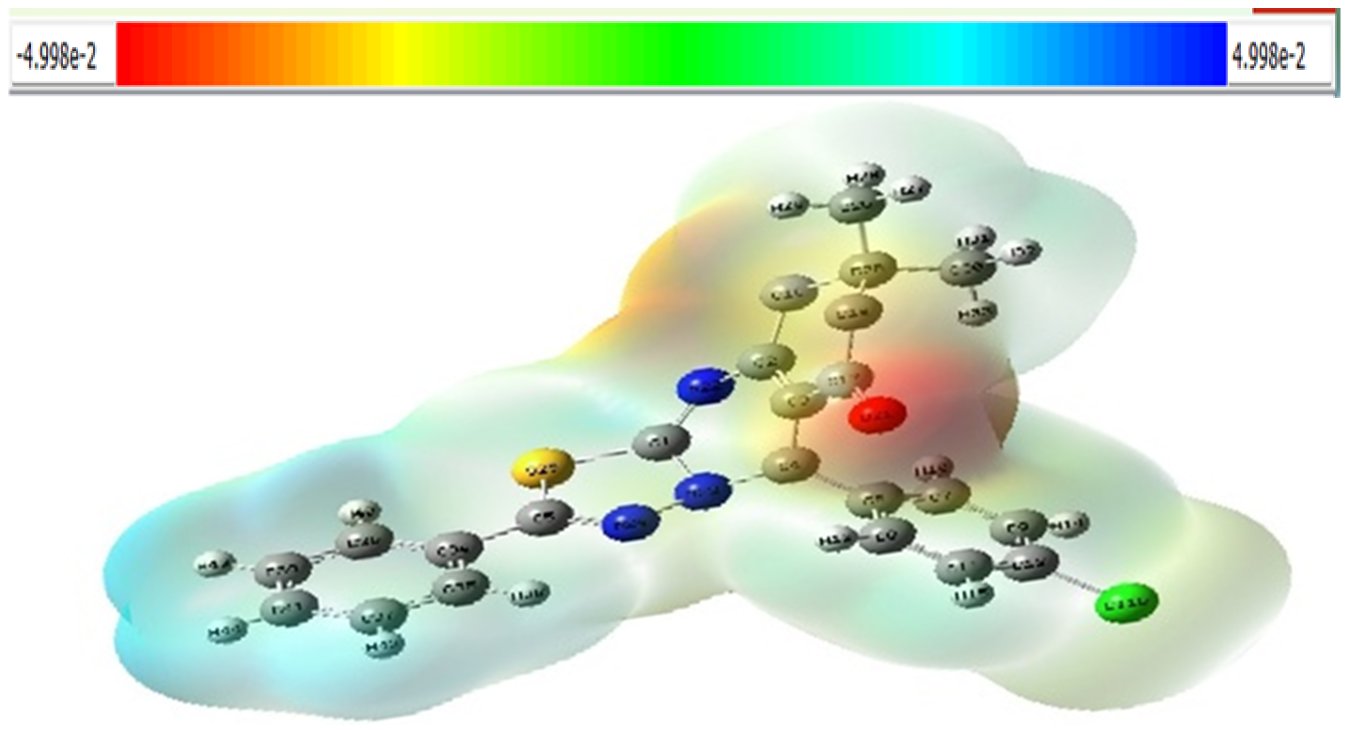	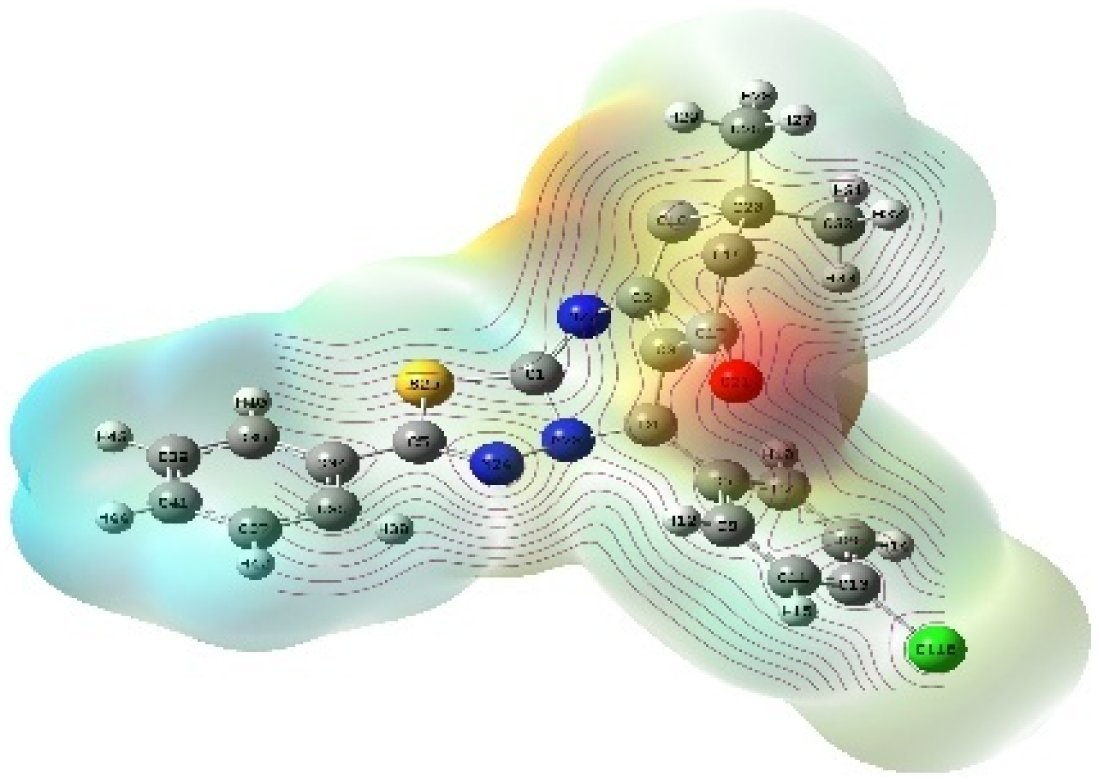
V5	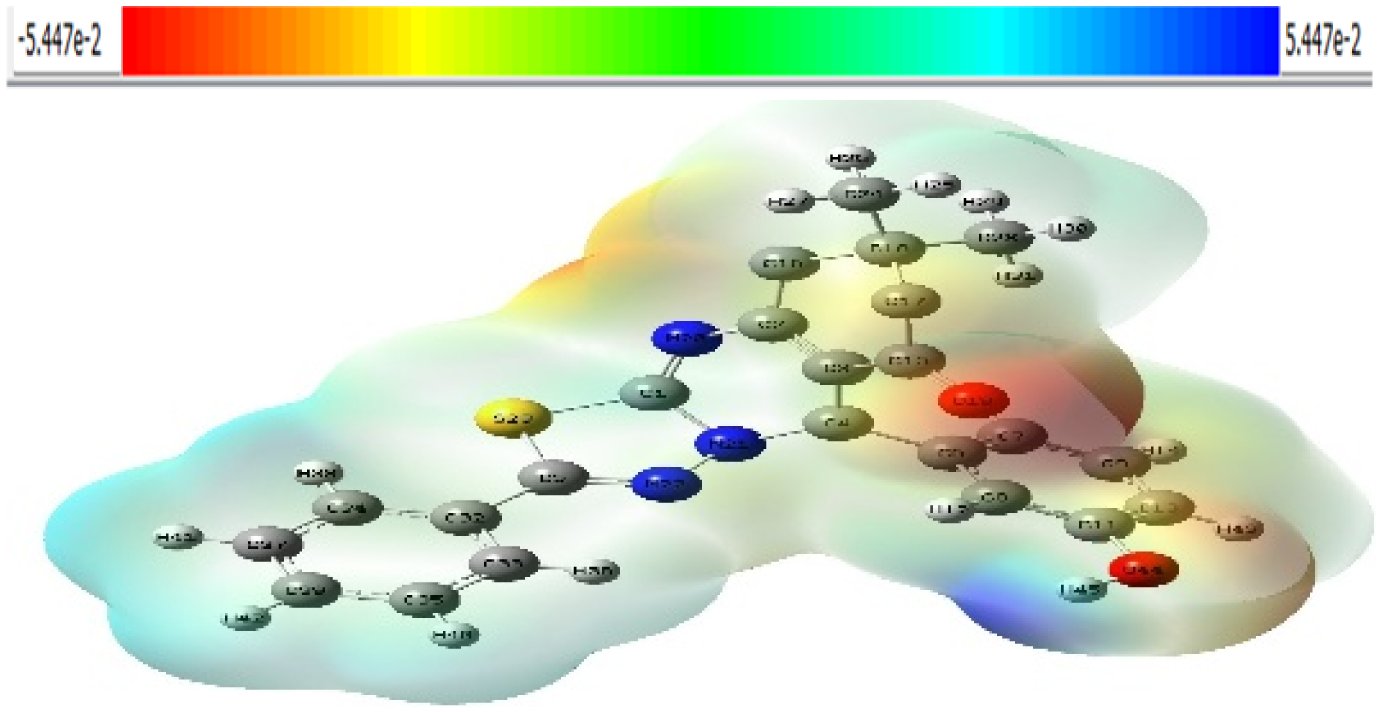	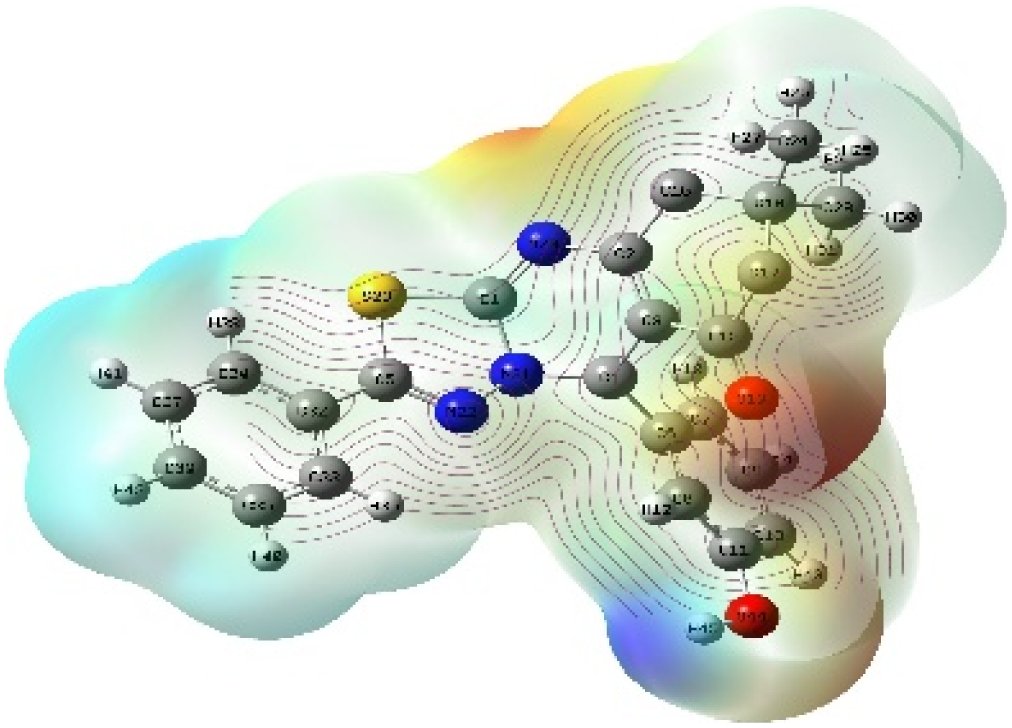
V7	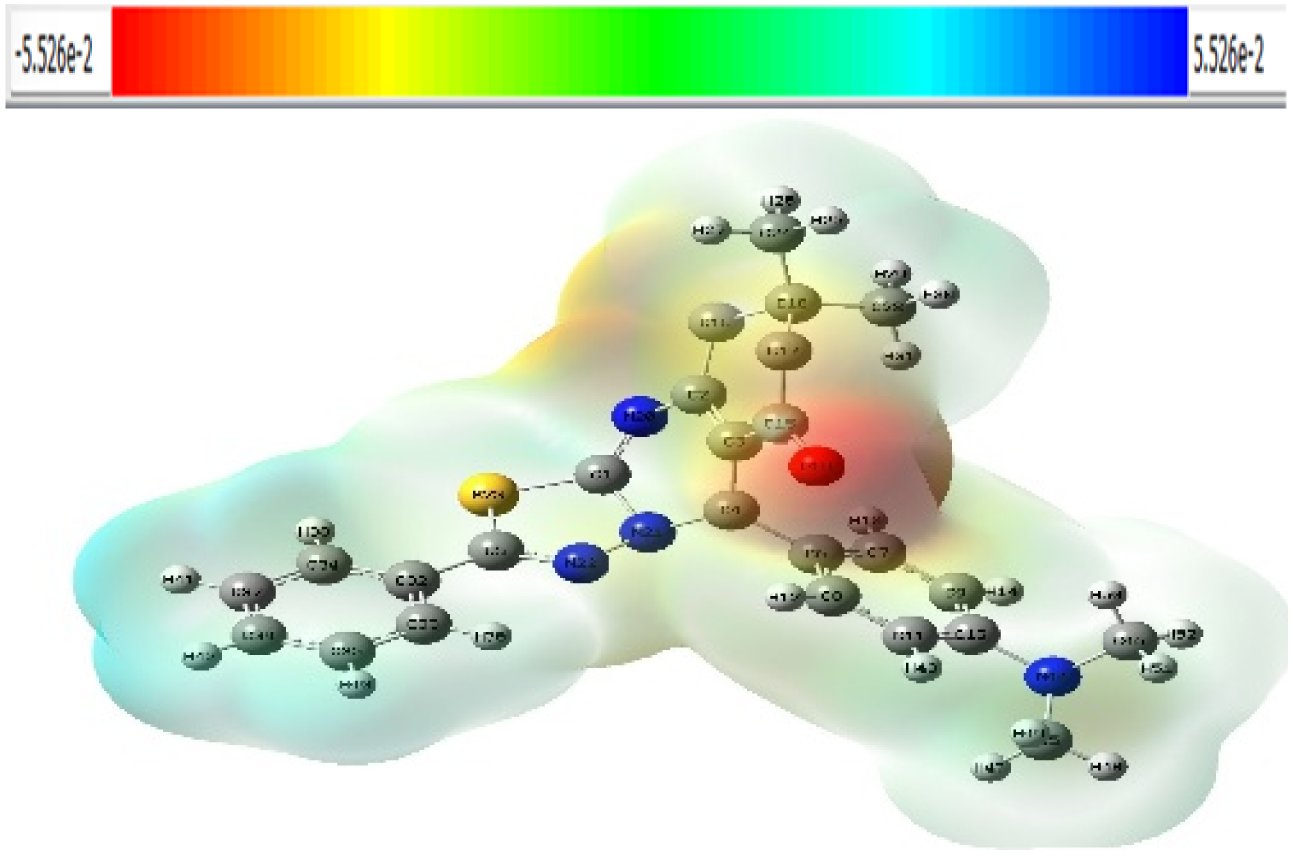	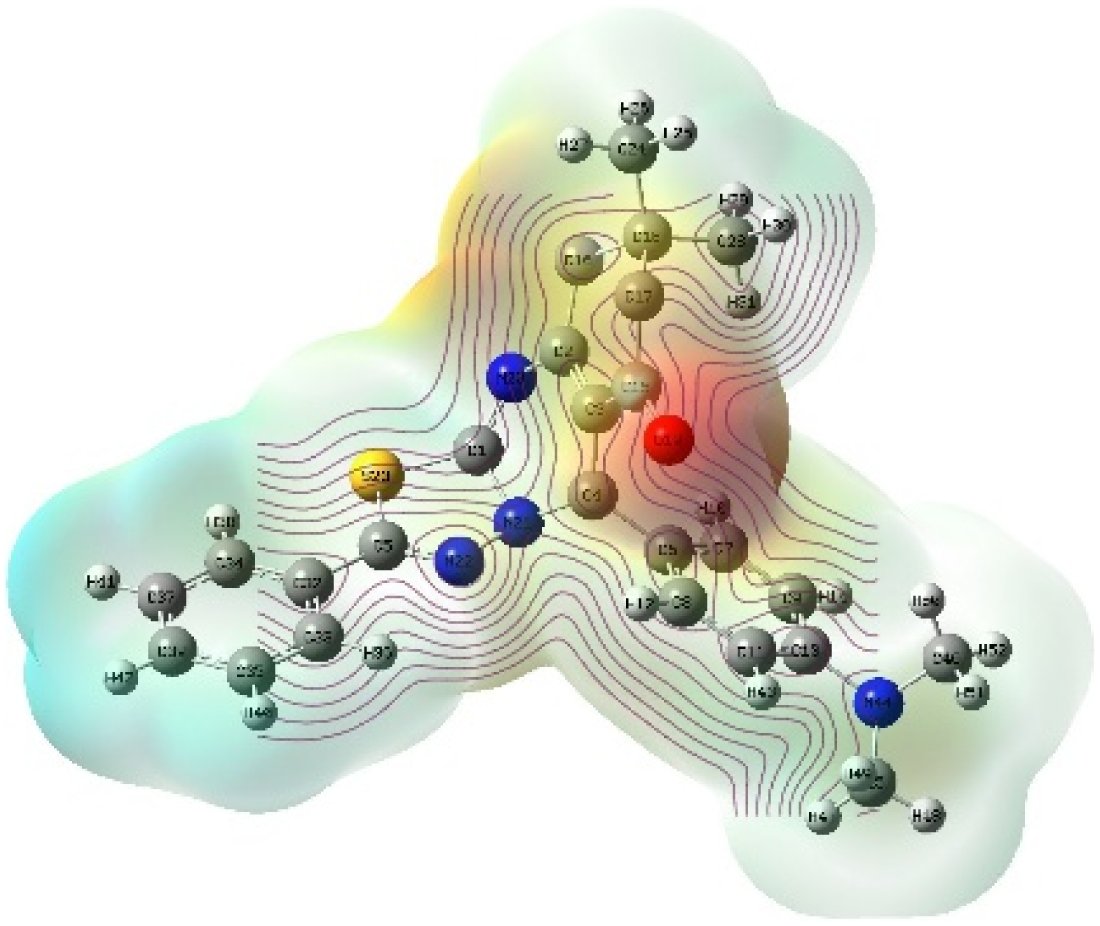

### 2.4. Molecular docking

#### 2.4.1. Docking analysis and correlation with antibacterial activity

The AutoDock Tools 1.5.4 was used to prepare the input files for molecular docking.^57–60^ Molecular docking analysis was carried out to assess the structural rationale underlying the antibacterial phenotype of the synthesized ligands (V1–V6) against *Escherichia coli* by modeling their interactions with the putative target protein 4DUH.^26,27,35,93^ The co-crystallized ligand displayed high binding affinity (−9.4 kcal mol^−1^) shown in Table 7, forming a key hydrogen bond with Asn46, extensive π-alkyl interactions with Ile78, Ile94, Pro79, and Val120, and stabilizing electrostatic interactions with Glu50, Arg76, and Lys103.^26^ These residues collectively define a predominantly hydrophobic binding pocket flanked by polar and charged hotspots that are important for ligand anchoring.

**Table 7:** Docking scores of the investigated compounds against 4DUH

Ligand	Binding affinity (kcal mol^−1^)
Co-crystallized_ligand	−9.4
V1	−6.7
V2	−7.1
V3	−6.8
V4	−6.9
V5	−6.7
V6	−6.4

It should be noted that the small differences in docking scores among V1–V6 (ranging from −6.4 to −7.1 kcal mol^−1^) fall within the standard error margin of the AutoDock Vina scoring function and should not be interpreted as meaningful quantitative rankings.

A range of docking energies for these analogs was obtained ranging from −6.4 to −7.1 kcal mol^−1^. In addition to the common type of interaction seen with the reference ligand, *i.e.*, π-alkyl contact with Ile78, Ile94 and Pro79, each member of the series showed a secondary hydrogen bonding interaction with Arg136.^26^ Therefore, it can be concluded that orientation of heteroatoms towards this residue is important for providing some degree of stabilization to the binding of the thiadiazoloquinazolinones shown in Fig. 15.

**Fig. 15:**
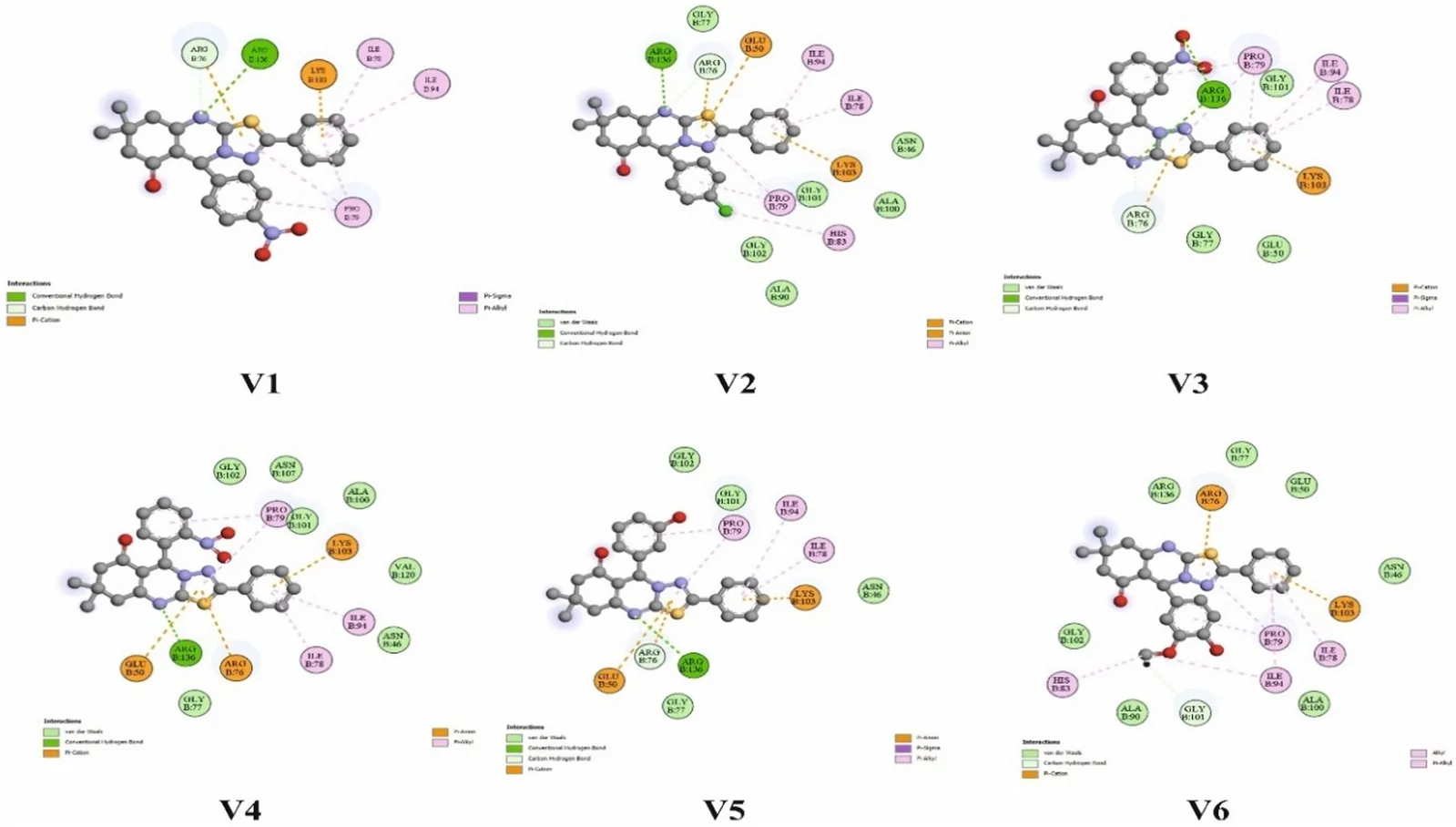
2D interaction analysis of docking results.

Compound V2 had the best docking energy (−7.1 kcal mol^−1^) yet the smallest zone of inhibition (16 mm) when tested against *E. coli* at 20 mg mL^−1^. An aromatic ring substituted by chlorine in V2 has improved its hydrophobic surface area and polarity compared to unsubstituted compounds. Therefore, it provides additional π-alkyl contact sites (*e.g.*, with His83) along with balanced electrostatic contact sites with Glu50 and Lys103.

When we examine compounds V1 and V5—both of which had equivalent docking scores of −6.7 kcal mol^−1^, the difference in their observed inhibition zones was notable as evidenced by zone sizes at 20 mg mL^−1^ of 17 mm and 20 mm respectively. While both ligands maintain the expected hydrophobic contacts as well as similar contact sites within the active site of Lys103, the variation in the substituents used likely produces variations in activity that are due to factors beyond the initial binding affinity. Compound V1 has an aromatic ring containing a nitro group, which will contribute to the rigidity of the molecule as well as create unfavorable conditions for desolvation effects. Conversely compound V5 contains a phenol/alkoxy framework that allows for increased hydrogen bonding capabilities creating greater flexibility in terms of adapting its position relative to the binding pocket and therefore potentially improving both the ability to bind and cell permeability. These findings highlight how useful docking scores can be as a predictor of biological activity when considering only static binding affinities. Compound V6 contained the lowest docking score in this set of compounds (−6.4 kcal mol^−1^) and displayed the highest level of antibacterial activity (zone size at 20 mg mL^−1^ = 21 mm). A comparison of its structure to those compounds with higher docking scores indicated that it does not have a strong hydrogen bonded interaction with Arg136, while all other compounds do. In addition, the presence of an alkoxy group in compound V6 could improve either its solubility and/or membrane penetration properties. Therefore, while its low docking score would suggest a lower predicted affinity for the target enzyme compared to other compounds in this study, its high antibacterial activity suggests that there are multiple contributors to the effectiveness of antibacterial agents. Overall, it must be acknowledged that the static docking scores show little quantitative correlation with the experimental antibacterial data. This is scientifically expected, as whole-cell zone-of-inhibition data are heavily influenced by solubility, membrane permeability, and efflux mechanisms that are not captured by target-specific computational docking. Additionally, while compounds V1 and V5 demonstrate a nonintuitive relationship between docking results and antibacterial potency (*i.e.*, they exhibit very different antibacterial potencies while having similar docking results), combined with the fact that compounds V2 (the highest docking result) and V6 (the lowest docking result) were among the most effective inhibitors of *Escherichia coli* growth, demonstrates that structural parameters such as interaction stability, flexibility in terms of adapting conformationally to the active site, and solvent mediated effects play a significant role in determining biologic activity independent of static docking predictions. Thus, compounds V1, V2, V5, and V6 were chosen for molecular dynamics studies to further investigate the time dependent stability of important interactions, protein–ligand flexibility, and solvation behavior and provide a link between docking predictions and observed antibiotic response.^26,57^ Prior to docking, crystallographic waters were removed, and appropriate protonation states were assigned to the protein at physiological pH. The grid box was defined with center coordinates of *X* = 32.3901, *Y* = 6.8671, and *Z* = 5.8420, and dimensions set to *X* = 24.3942 Å, *Y* = 27.0675 Å, and *Z* = 24.3067 Å. The docking exhaustiveness was set to 8.

#### 2.4.2. Molecular dynamics simulations

Molecular dynamics simulations were utilized for a better understanding of how and which components are dynamically responsible for antibacterial activity.^36–38,61^ Four compounds V1 and V5 (same docking score), V2 (the compound with the best docking score), and V6 (the compound with lowest docking score however, the highest phenotypic activity) were evaluated in terms of their molecular dynamic behavior over 100 ns shown in Fig. 16.

**Fig. 16:**
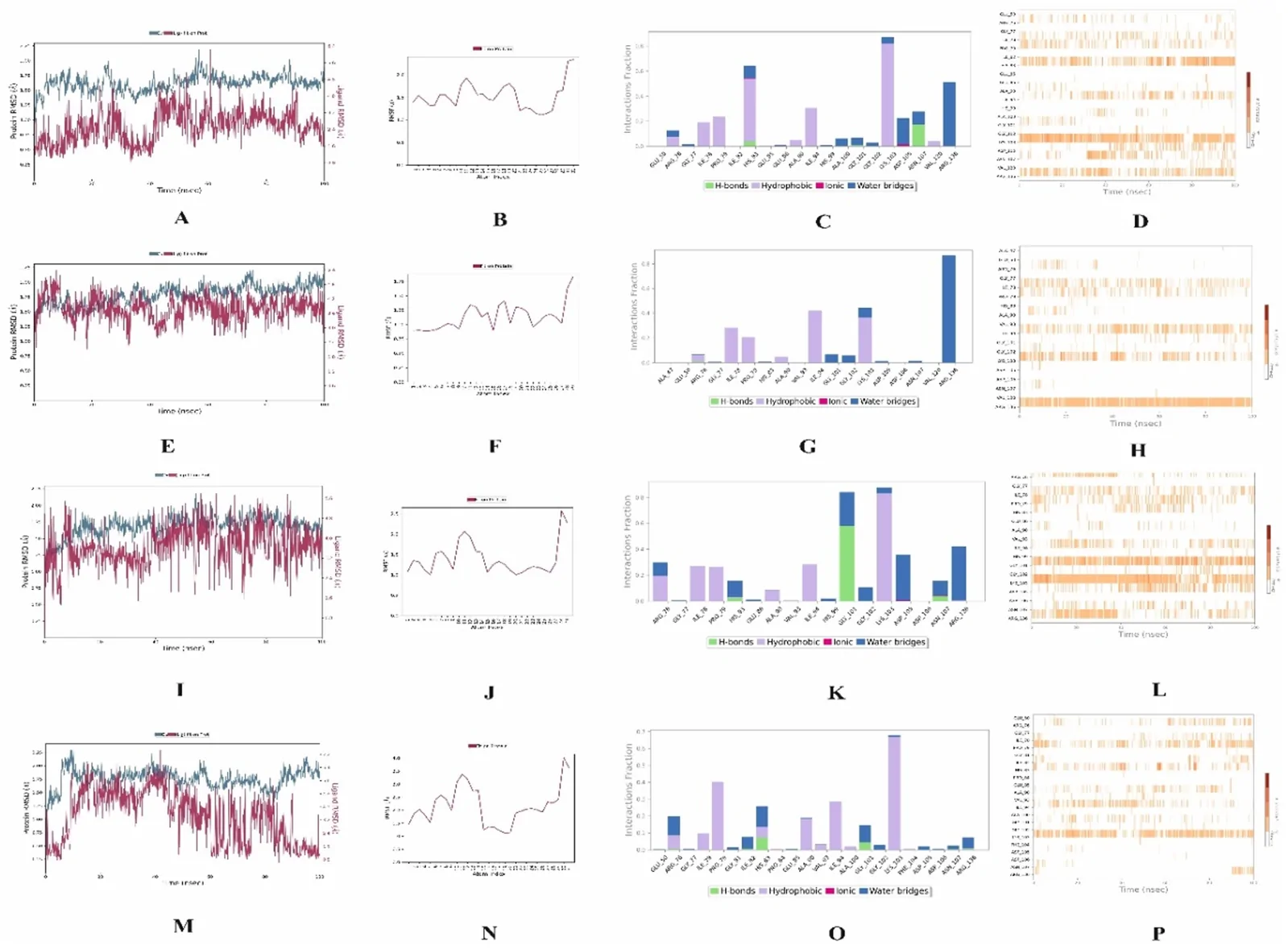
MD simulation results: RMSD (A, E, I and M), ligand RMSF (B, F, J and N), contact interactions (C, G, K and O), and interaction timelines (D, H, L and P) for V1 (A–D), V2 (E–H), V5 (I–L), and V6 (M–P).

Simulation stability was assessed by evaluating the convergence and plateau behavior of the RMSD trajectories over time.^36,37^ The compound V1 demonstrates a constant binding model through the entire simulation. The protein–ligand RMSD for V1 equilibrated and reached a plateau around 3.0 Å, indicating a stable trajectory over the simulation timeframe. Additionally, the ligand's RMSF remained mostly constant at roughly 1.5 Å; however, there are some localized RMSF fluctuations as large as 2.0 Å due to the nitro-substituted aromatic group. While the nitro-substituted aromatic group provides additional polarity to the compound, it limits the ability of the compound to adaptively bind within the pocket. An examination of the interactions between the protein and ligand reveals continued hydrophobic interactions with residues Ile78, Pro79, Ile94, His83, and Lys103.^26^ Also, the compound maintains a stable hydrogen bonding interaction with residue Asn107, and a transient interaction with Arg136. Although the position of the compound remains relatively stable in the pocket, the compound has high solvent accessibility (the solvent accessible surface area, SASA fluctuates between 150 and 300 Å^2^) and compactness (the radius gyration, rGyr is in the range of 4.7 to 4.9 Å) consistent with moderate antibacterial activity despite having a similar docking score to compound V2.

V5, with a similar docking score to V1 (−6.7 kcal per mole), had significantly better antibacterial efficacy (20 mm *vs.* 17 mm). MD simulations provided comparable levels of stability (RMSD ∼4.0 Å) but different interaction profiles; the alkoxy group of V5 facilitates increased hydrogen bonding versatility, as evidenced by the continued presence of hydrogen bonds with Gly101 and strong hydrophobic contacts with Arg76, Ile78, Pro79, Ile94 and Lys103. Like Gly101, V5 maintains ongoing contact with Lys103 over the entire course of the simulation; however, unlike V1, V5 maintains some level of peripheral flexibility (ligand RMSF ∼1.0 Å, where one of the methylene groups extends ∼2.5 Å). A relatively small range of SASA (150–250 Å^2^) and constant rGyr (range ∼4.6–4.8 Å) suggest a smaller, dynamically optimal binding configuration. Therefore, these data suggest a possible explanation for why V5 has improved bioactivity relative to V1, as well as demonstrate the importance of persistent interaction formation and ligand conformational adaptability to determine phenotypically relevant activities. Although compound V2 has the highest docking score (−7.1 kcal per mole), it exhibits a stable protein–ligand complex during the simulation time frame, with RMSD ∼4.2 Å. The chlorinated aryl ring of V2 may increase the hydrophobic character of the aromatic ring as well as increase the degree of polarization at the expense of hydrogen-bonding potential. Consistent with this prediction, V2 forms primarily hydrophobic contacts with Ile78, Pro79, Ile94 and Lys103. Contact with Arg136 is maintained for much of the simulation. Low values for ligand RMSF (∼1.0 Å) indicate that the binding site remains relatively fixed with very little opportunity for conformational motion; thus, few persistent polar contacts are observed for V2 compared to V5 in addition to slightly larger SASA (150–275 Å^2^). This combination suggests a stable binding site that lacks reinforcement *via* dynamic processes, consistent with the moderate experimental zone of inhibition (16 mm at 20 mg mL^−1^) of V2 despite being docked with greater affinity. The most dramatic example was V6, which exhibited the lowest docking score (−6.4 kcal per mole) yet produced the largest inhibition zone (21 mm). Dynamic behavior during MD simulations resulted in a wide variation in RMSD (range 1.6–4.8 Å); the phenyl ring directly attached to the thiadiazolone nucleus remained firmly anchored within the receptor binding pocket for virtually all of the simulation time. In contrast, the majority of the molecule underwent rotational rearrangement suggesting a possible “pivot-anchor” type binding mechanism in which a highly stable aromatic anchor occupies the binding pocket while maintaining periphery flexibility for adapting interactions. As such, V6 periodically formed hydrogen bonds with His83 and Gly101 as well as contacting Lys103 and forming hydrophobic contacts with Arg76, Ile78, Pro79, His83, Ala90, and Ile94. While SASA values were significantly higher for V6 (indicating significant exposure to solvent) a variety of mechanisms exist whereby the increased dynamic adaptability and anchoring capabilities can potentially compensate for lower static affinity; which is consistent with the observed discrepancy between the docking score and the phenotypic data, highlighting the possible contributions of kinetic factors and molecular plasticity to drug activity^37,38,62^ (Fig. 17).

**Fig. 17:**
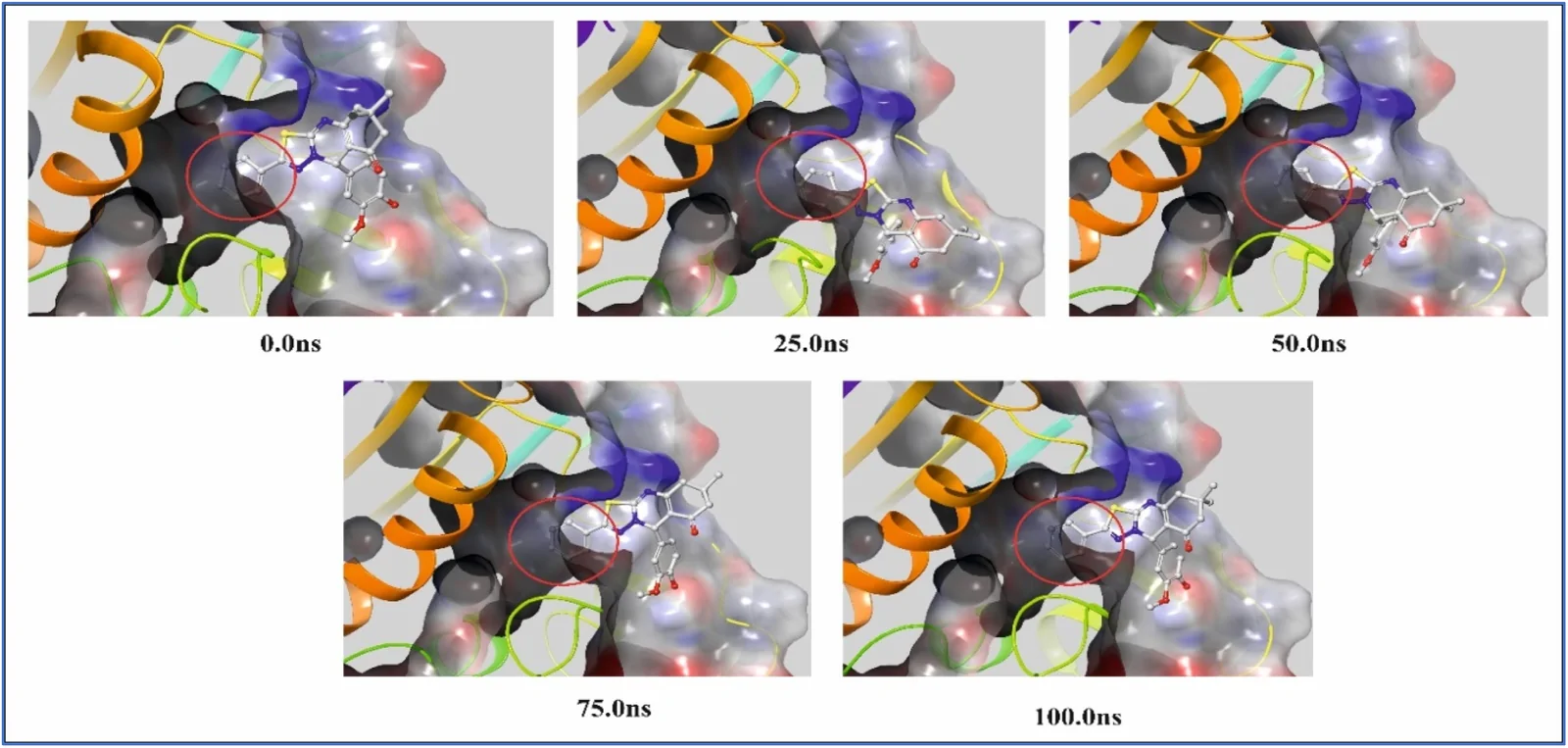
Pivot anchored binding mode of V6.

Collectively, the molecular dynamics data provide an explanation for why docking is able to make accurate predictions about what should be active against bacteria based on experimental antibacterial activity. Docking was correct when it identified important amino acid residue contacts for docking as well as the overall ranking trend of affinities; however, docking did not address other important issues such as how long the interactions last, how flexible the molecules are during the interaction, or how stable the interactions are.^36–38^ Through MD simulation analysis, these additional factors were shown to be very important contributors to the observed phenotypic potencies. Overall, this illustrates the need for inclusion of MD simulations within structure–activity relationship studies so that the dynamics involved in protein–ligand recognition can be captured and correlated with resultant biological efficacies.

### 2.5. Plausible reaction mechanism

A suitable pathway to the synthesis of thiadiazolo[2,3-*b*]quinazolin 6-ones has been proposed in Scheme 2(a–c).^30–32,77^ The first stage involves the Knoevenagel condensation of arylmethanealdehyde with dimedone resulting in an intermediate product denoted as “arylmethylene–dimedone” Scheme 2(a). The metal active sites on the WFe_2_O_4_/rGO nanoparticals coordinates with the oxygen atom of carbonyl groups that increases the electrophilicity of the aldehyde carbonyl carbon speeding the initial condensation to form an intermediate “arylmethylene–dimedone”. The second stage involves a Michael reaction where 2-amino-5-phenyl-1,3,4-thiadiazole acts as a nucleophile to add to the intermediate Scheme 2(b). The catalyst continues to coordinate with the intermediate (a) this makes the system more reactive towards nucleophilic attacks by amino group of thiadiazol to form intermediate (b). This results in the cyclized compound through elimination of a water molecule as shown in (c).^29,32^ The catalyst assists in proton shifting and stabilizes leaving hydroxyl group as protonated oxonium ion that lowers the activation energy required for the elimination of water molecules and cyclization to yield stable Scheme 2(c) thiadiazolo[2,3-*b*]quinazolin 6-ones derivatives.^29,32^ The function of reduced graphene oxide is to provides a high surface area carbon support that prevents the aggregation of magnetic nanoparticles, ensuring maximum exposure of active catalytic sites to the reactants.

### 2.6. Catalyst recyclability study

To further highlight the practical advantages of the developed protocol, we studied the recyclability of the WFe_2_O_4_/rGO catalyst, as shown in Fig. 18.^2–4,10^ The recyclability and reusability of the catalyst were evaluated for the synthesis of thiadiazolo[2,3-*b*]quinazolin 6-one under the optimized reaction conditions. After each catalytic cycle, the catalyst was isolated from the reaction mixture using the external magnet, thoroughly washed with ethanol and ethyl acetate, and oven dried under a vacuum oven before being reused for five subsequent cycles. The structural integrity of the catalyst was confirmed after five successive runs, with minimal loss of catalytic activity.^33,34^

**Fig. 18:**
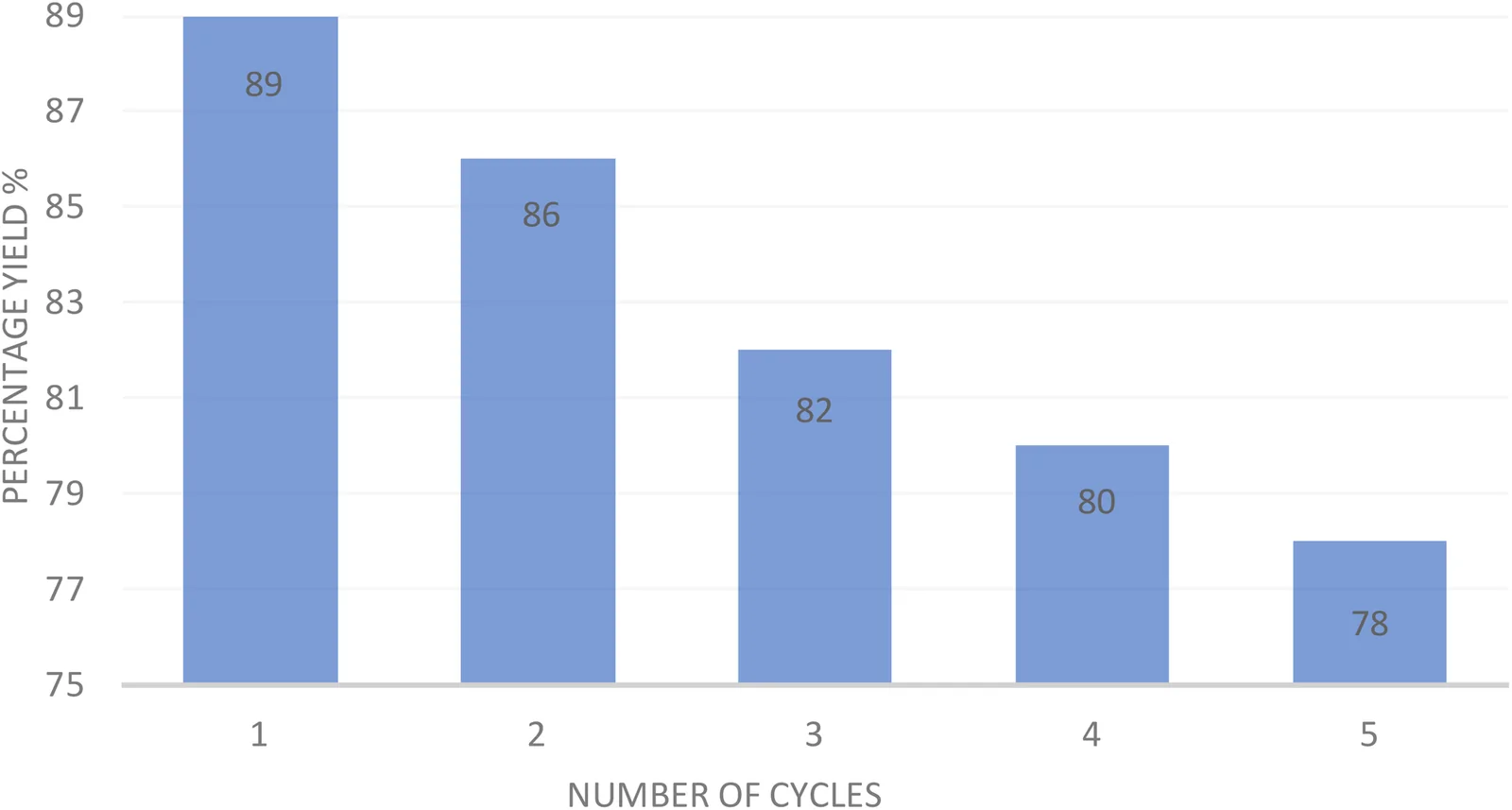
Reusability of WFe_2_O_4_/rGO in the synthesis of thiadiazolo [2,3-*b*] quinazolin-6 ones (V1–8).

### 2.7. Hot filtration test

The heterogeneous nature of the nanocatalyst was studied *via* hot filtration or leaching test.^33,34,94–96^ In this method, the synthesis of thiadiazolo[2,3-*b*]quinazoline-6-one derivatives (V1–V8) was carried out in the presence of WFe_2_O_4_/rGO nanocatalyst under the optimized reaction conditions. The reaction was allowed to proceed for 1 h (approximately half of the total reaction time), after which the reaction mixture was divided into two equal halves. From one-half of the reaction mixture, the catalyst was magnetically separated, while the other portion was left with the catalyst intact. Both portions were then allowed to continue under the same conditions for an additional 2 h.^33,34^ It was observed that no further formation of thiadiazolo[2,3-*b*]quinazoline-6-one occurred in the portion from which the catalyst was removed, while the portion containing the catalyst proceeded smoothly to completion, yielding the desired product in 89%.^33,34,96^ These findings are consistent with predominantly heterogeneous catalytic behavior with limited leaching under these conditions. Hot filtration, together with EDX and FT-IR analysis of the recovered solid, indicates that no detectable change in catalyst composition occurs after reaction; however, these methods do not quantify the concentration of dissolved metal species in the filtrate, and trace leaching below their detection limits cannot be excluded without elemental analysis (*e.g.*, ICP-OES/ICP-MS) of the reaction filtrate, which was not performed in this study.

## 3 Conclusion

In conclusion, a magnetically recoverable WFe_2_O_4_/rGO nanocatalyst was synthesized *via* a hydrothermal route and applied to the efficient synthesis of thiadiazolo[2,3-*b*]quinazolin-6(7*H*)-one derivatives (V1–V8) in good to excellent yields under ambient, aqueous (ethanol:water) conditions. The catalyst combines operational simplicity, low cost, limited leaching as indicated by hot filtration and spectroscopic analysis, and straightforward magnetic separation and reuse. DFT calculations revealed a significantly lower HOMO–LUMO energy gap in the aqueous phase than in the gas phase, attributable to stronger heteroatom–water interactions. Molecular docking identified key binding interactions against the target protein, while molecular dynamics simulations resolved cases where docking scores alone could not explain the observed antibacterial potency, underscoring the importance of binding-site dynamics and ligand flexibility in structure–activity relationships.

## 4 Experimental

### 4.1. Materials and chemicals

All chemicals and solvents used in this work were obtained from Sigma-Aldrich and were used without further purification. The substrate purity and the reaction progress were monitored by using silica-coated TLC plates under a UV lamp. Thin-layer chromatography was performed using a silica gel-coated aluminium plate. Identification of the compounds was carried out by melting point measurement, determined in an open capillary tube, and is uncorrected.

### 4.2. Characterization tools

The as-prepared nanocatalyst was thoroughly characterized using various techniques to gain insights into its surface morphology, functionality, composition, stability, and other essential characteristics. The FT-IR studies were carried out on a Bruker Alpha spectrophotometer using KBr pellets. X-ray diffractograms were acquired on a Bruker D8 instrument using Cu Kα1 radiation with *λ* = 1.5418 Å in the range 2*θ* = 20–80° at a scan rate of 2° min^−1^. The FE-SEM micrographs were collected using a JEOL JSM-IT200 instrument and further supported with the X-ray energy dispersive spectroscopy. A magnetization value of the nanocatalyst was measured by using a vibrating sample magnetometer at room temperature between −20 000 and +20 000 Oe.^44–46^ Finally, the synthesized molecules were characterized using ^1^H (400 MHz) and ^13^C (100 MHz) Brucker Avance spectrometers. Spectra were recorded using DMSO-d_6_ as dissolving solvent and TMS as an internal standard. GC-MS analysis was carried out on a Shimadzu QP2010 spectrometer to detect the mass of the synthesized compounds.

### 4.3. Preparation of WFe_2_O_4_/rGO nanocatalyst by hydrothermal method

#### 4.3.1. Preparation of reduced graphene oxide

The graphene oxide was prepared from natural graphite flakes using the modified Hummers route.^47,48^ 75 mL of concentrated sulfuric acid was mixed with 3 g of graphite flakes, 1.5 g of sodium nitrate, and 9 g of KMnO_4_ under continuous stirring. The mixture was kept in an ice bath at a low temperature under magnetic stirring at 250 rpm. After 20 min, 3 g of KMnO_4_ was slowly added to the mixture of GO solution and continuously stirred for 12 h at low temperature.^49^ After this period, the resulting mixture was shown to be a brown-colored viscous liquid. Additionally, 5 g of KMnO_4_ was added to the aforementioned suspension, and the suspension turned into a dark brown precipitate. In this step of the process, the obtained mixture undergoes the reduction process while using 30% H_2_O_2_ with double distilled water solution. Furthermore, the resultant suspension was subjected to the centrifugation process and was washed 10% with hydrochloric acid and DDW water to remove impurities and some hydrated metal ions remaining in the suspension. Finally, the resultant product was dried at 80 °C overnight to obtain the crystalline graphene oxide powder. Reduced graphene oxide powder was obtained through the reduction of graphene oxide powder. The graphene oxide powder dissolved in 150 mL distilled water and sonicated for 2 h. Then 15 mL of 90% hydrazine hydrate was added to the suspension and pH was adjusted to 10 using 0.1 M NaOH solution.^50,51^ Then the reaction mixture was kept at 80 °C for 7 h, then filtered and washed with ethanol and water several times. The reduced graphene oxide (rGO) powder was obtained after drying at 70 °C for 24 h.

#### 4.3.2. Preparation of WFe_2_O_4_ NPs by a hydrothermal method

The synthesis of WFe_2_O_4_ NPs was carried out by a hydrothermal method.^52–55^ The 0.2949 g Na_2_WO_4_·2H_2_O was dissolved in 30 mL of distilled water. The 0.5796 g of Fe(NO_3_)_3_·9H_2_O was mixed in 30 mL of deionized water. The two solutions were mixed with constant stirring. The resulting solution was stirred at room temperature for 2 h. After that, 15 mL of 30% NH_4_OH solution was added to the mixture to adjust the pH value; the mixture was stirred for 3 h. The mixture was then put into a Teflon-lined autoclave and kept at 200 °C for 8 h. The precipitate was then washed with distilled water and ethanol. The precipitate was then dried in an oven at 90 °C for 6 h. Thus, as prepared, powdered WFe_2_O_4_ nanoparticles were finally obtained.

#### 4.3.3. Preparation of WFe_2_O_4_/rGO nanocatalyst by hydrothermal method

The graphite flakes are oxidized to graphene oxide by well-established modified Hummers' methods.^47,48^ The graphene oxide was reduced by using hydrazine hydrate.^50,51^ The synthesis of WFe_2_O_4_/rGO NPs by the hydrothermal method. Typically, a certain amount of reduced graphene oxide was dispersed in 40 mL of distilled water and sonicated for 1.5 hours. At the same time 0.5898 g Na_2_WO_4_·2H_2_O and 1.1592 g of Fe(NO_3_)_3_·9H_2_O were mixed in 40 mL of deionized water. After that, the reduced graphene oxide dispersion was added to the above mixture with constant stirring. The resulting solution was stirred at room temperature for 2 h. After that, the 25 mL 30% NH_4_OH solution was added to the mixture to adjust the pH value of mixture; it was stirred for 3 h. The mixture was then put into a Teflon-lined autoclave and kept at 200 °C for 8 h. The precipitate was then washed with distilled water and ethanol. The precipitate was then dried in an oven at 90 °C for 6 h.^7,56^ Thus, as-prepared, powdered WFe_2_O_4_/rGO NPs were finally obtained.

### 4.4. General procedure for the synthesis of thiadiazoloquinazolinone derivatives

The condensation of 2-amino-5-phenyl-1,3,4-thiadiazole (S) (1 mmol, 0.177 g), dimedone (U) (1 mmol, 0.140 g), and different substituted aromatic aldehydes (T1–T7) (1 mmol) were added to 2.5 mL water and 2.5 mL ethanol in the presence of magnetic WFe_2_O_4_/rGO NPs catalyst (15 mg) in 100 mL round bottom flask.^2,3,10^ At room temperature, the reaction was continually stirred. Thin-layer chromatography in an ethyl acetate and petroleum ether (20 : 80, v/v) solvent solution was used to monitor the reaction's progress. Upon completion of the reaction, the nanocatalyst was separated by applying a strong external magnet, and the corresponding crude product was filtered and recrystallized from hot ethanol to afford the desired thiadiazolo[2,3-*b*]quinazoline-6-ones derivatives (V1–V8) (Scheme 1).

**Scheme 1:**
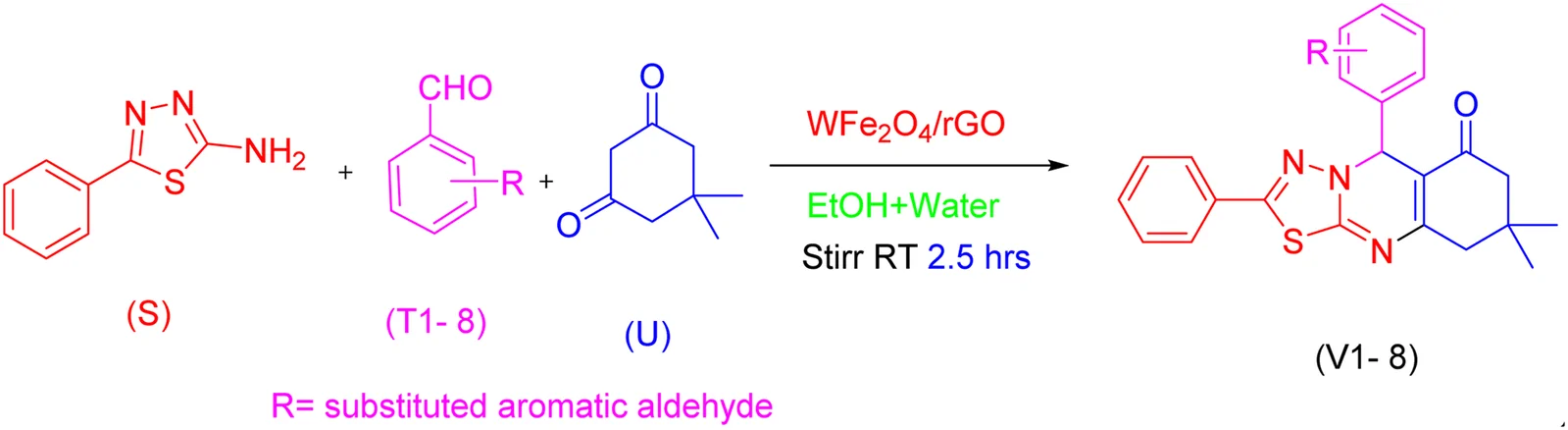
Synthesis of thiadiazolo [2,3-*b*] quinazolin-6-(7H)-ones (V1–8) promoted by the WFe_2_O_4_/rGO nanocatalyst.

**Scheme 2:**
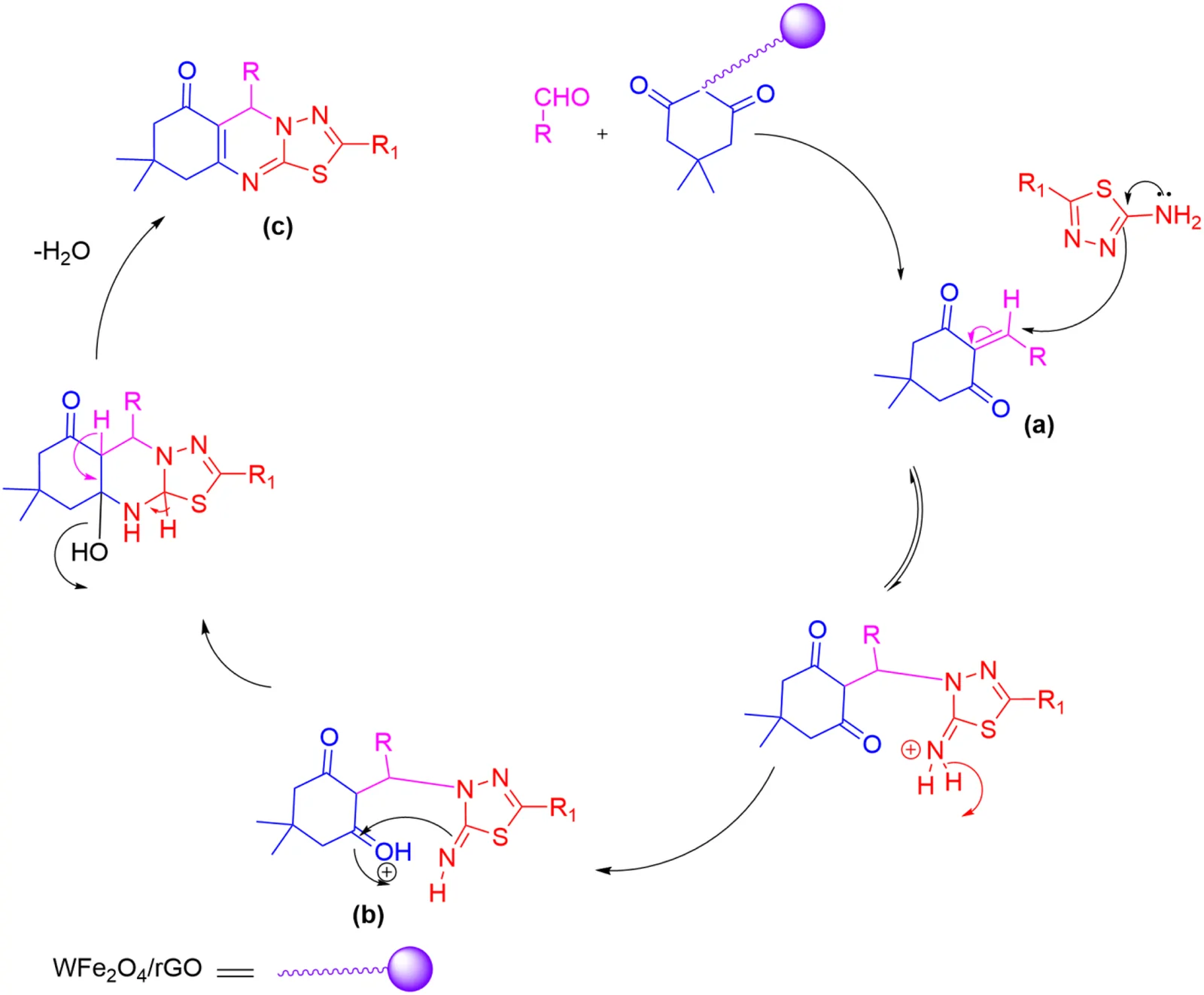
Proposed mechanism for the synthesis of thiadiazolo[2,3-*b*]quinazolin-6(7*H*)-one derivatives: (a) arylmethylene-dimedone, (b) intermediate, and (c) derivative.

### 4.5. Computational details for the molecular docking study

#### 4.5.1. Protein preparation

Crystal structure of the target protein (PDB ID: 4DUH, *E. coli* DNA gyrase B) was obtained from the Protein Data Bank and was prepared for docking and MD simulations by removing non-essential molecules, adding hydrogen atoms, and structurally validating integrity using UCSF Chimera.^26,57,58^

#### 4.5.2. Ligand structure preparation

The structures of all the ligands (V1–V6) were sketched and geometry-optimized using MarvinSketch using the MMFF94 force field to acquire energetically feasible structures (Marvin 25.3.5, ChemAxon, https://www.chemaxon.com).^57^

#### 4.5.3. Molecular docking

Molecular docking analysis was performed using PyRx with the AutoDock Vina scoring engine.^57–60^ The binding site was defined according to the position of the co-crystallized ligand, and a uniform grid box was applied across all ligands to ensure comparability of docking poses. Docking poses were ranked by predicted binding affinity, and the highest-scoring conformations were selected for further analysis. Protein–ligand interactions were examined using BIOVIA Discovery Studio Visualizer and UCSF Chimera.

#### 4.5.4. Molecular dynamics simulations

Molecular dynamics simulations were performed using the Desmond module for complexes V1, V2, V5, and V6.^36–38,61^ All systems were solvated in explicit aqueous solution, neutralized with counterions, energy-minimized, equilibrated, and simulated for 100 ns under NPT conditions (*T* = 300.0 K, *P* = 1.01325 bar). Trajectory analyses included root-mean-square deviation, ligand root-mean-square fluctuation, solvent-accessible surface area, radius of gyration (rGyr), and protein–ligand contact persistence. Computational results were correlated with antibacterial activity by measuring inhibition zones at 20 mg mL^−1^.^37,38,62^

## Author contributions

Varsharani S. More: writing – review & editing, writing – original draft, methodology, investigation, formal analysis, data curation, conceptualization. Vaibhav B. Sankpal: methodology, investigation, formal analysis, data curation. Asha D. Patil: formal analysis, data curation. Santosh S. Chobe: investigation, formal analysis, data curation. Kuldeep T. Padhyare: validation, methodology, Navanand B. Wadwale: validation, methodology, Vijay N. Bhosale: formal analysis. Shrikant S. Nilewar: methodology, data curation. Sandeep G. Sontakke: review & editing, formal analysis. Lalit D. Bhosale: formal analysis. Sambhaji R. Mane: formal analysis. Gopinath S. Khansole: writing – review & editing, writing – original draft, data curation, validation, conceptualization.

## Conflicts of interest

The authors declare that they have no known competing financial interests or personal relationships that could have appeared to influence the work reported in this paper.

## Supplementary Material

RA-016-D6RA06264D-s001

## Data Availability

The data supporting the findings of this study are available in the supplementary information (SI) accompanying this article. Supplementary information: additional experimental and computational details, including DFT-calculated geometrical parameters (bond lengths and bond angles; Tables S2–S5) and spectroscopic characterisation data (NMR, IR and mass spectra) for the synthesised compounds. See DOI: https://doi.org/10.1039/d6ra06264d.
