# Supplementary material for: Magnetically recoverable WFe2O4/rGO catalyzed green synthesis of thiadiazolo[2,3-b] quinazolin-6(7H)-one derivatives: structural, DFT, and molecular docking studies
Source: RSC Adv. 2026 Sep 10;16(43):53959–80. doi: 10.1039/d6ra06264d (PMC13559896; doi:10.1039/d6ra06264d)
Supplement: Supplementary file 1 [file RA-016-D6RA06264D-s001.pdf]

Supplementary Information (SI) for RSC Advances.
This journal is © The Royal Society of Chemistry 2026

Magnetically Recoverable WFe_2O_4/rGO Catalyzed Green Synthesis of Thiadiazolo[2,3-b] quinazolin-6(7H)-one Derivatives: Structural, DFT, and Molecular Docking Studies

Varsharani S. More[1,2], Vaibhav B. Sankpal[2], Asha D. Patil[3], Santosh S. Chobe[4], Kuldeep T. Padhyar[5], Navanand B. Wadwale[5], Vijay N. Bhosale[6], Shrikant S. Nilewar[7], Sandeep G. Sontakke[8], Lalit D. Bhosale[9], Sambhaji R. Mane[1], Gopinath S. Khansole*[2]

[1]Department of Chemistry, K. R. P. Kanya Mahavidyalaya, Urun-Ishwarpur, Sangli-415409 (M.S.) India.
[2]Department of Chemistry, Deshbhakt Anandrao Balwantrao Naik Arts and Science College, Chikhali, Sangli-415408 (M.S.) India.
[3]Department of Physics, Deshbhakt Anandrao Balwantrao Naik Arts and Science College, Chikhali, Sangli-415408 (M.S.) India.
[4]Department of Chemistry, Research Centre, M.G.V's Loknete Vyankatrao Hiray Arts, Science and Commerce College, Panchavati, Nashik-03(M.S.) India- 422003
[5]Department of Chemistry, P.G. Research Centre, M. S. G. College, Malegaon Camp, Malegaon, Nashik(M.S.) India- 423105
[6]Department of Chemistry, P.G. Research Centre, Yeshwant Mahavidyalaya, Nanded (M.S.) India- 431602
[7]Department of Pharmaceutical Chemistry, Maliba Pharmacy College, Uka Tarsadia University, Gujrat, India-394350.
[8]Department of Chemistry, Lonavala Education Trust's Dr. B.N. Purandare Arts, Smt. S.G. Gupta Commerce and Smt. Shardaben Amrutlal Mithaiwala Science College, Lonavala-410403, Pune, (M.S.), India.
[9]Department of Chemistry, Yashwantrao Chavan Warana Mahavidyalaya, Warananagar University, Warana Nagar, (M.S.) India- 416 114
Corresponding author Email: gopinathskhansole@gmail.com

Supporting Information

INDEX

1. Table of Grid box parameters used for molecular docking ……………………………...**S1**
2. Table of optimized Bond Length and Bond Angles Calculated at the 6-311++G(d,p) level of V1 molecule……………………………………………………………………………**S2**
3. Table of optimized Bond Length and Bond Angles Calculated at the 6-311++G(d,p) level of V2 molecule ………………………………………………………………………**S3**
4. Table of optimized Bond Length and Bond Angles Calculated at the 6-311++G(d,p) level of V5 molecule ………………………………………………………………………**S4**
5. Table of optimized Bond Length and Bond Angles Calculated at the 6-311++G(d,p) level of V7 molecule ………………………………………………………… …………..**S5**
6. General procedure for the synthesis of thiadiazolo quinazolinones derivatives(V1-V7)……………………………………………………………………………… **S6**
7. Spectroscopic data for products……………………………………………………**S7-S14**
8. Some spectra of representative compounds: ……………………………………**S15-S37**

……….S1…………………

Table : Grid box parameters used for molecular docking.

Parameter	X	Y	Z
Center	32.3901	6.8671	5.8420
Dimensions (Å)	24.3942	27.0675	24.3067

…………S2………………………

Computational Details: Density Functional Theory

Table: Optimized Bond Length and Bond Angles Calculated at the 6-311++G(d,p) level of V1 molecule

Bond lengths (Å)					
C1-S24	1.742	**N44-O46**	**1.238**	C25-H28	1.070
C1-N21	1.324	**N44-O45**	**1.238**	C5-N23	1.324
C1-N22	1.475	C11-H15	1.071	C5-S24	1.777
C2-C17	1.546	C11-C8	1.401	N23-N22	1.426
C2-C3	1.344	C8-H12	1.070	C5-C33	1.540
C3-C4	1.520	C2-N21	1.486	C33-C34	1.402
C3-C16	1.531	C16-O20	1.258	C33-C35	1.402
C4-N22	1.445	C16-C18	1.533	C35-H39	1.070
C4-C6	1.540	C18-C19	1.548	C35-C38	1.402
C6-C7	1.542	C19-C17	1.544	C38-H42	1.069
C6-C8	1.402	C19-C25	1.540	C38-C40	1.401
C7-H10	1.071	C19-C29	1.540	C40-H43	1.070
C7-C9	1.401	C29-H30	1.070	C40-C36	1.402
C9-H14	1.071	C29-H32	1.070	C36-H41	1.707
C9-C13	1.402	C29-H31	1.070	C36-C34	1.402
C13-C11	1.402	C25-H26	1.070	C34-H37	1.070
**C13-N44**	**1.472**	C25-H27	1.070		
Bond angles (Å)					
C36-C40- H43	119.4805	C19-C25-H27	109.4543	C9-C13- H14	120.0307
C43-C40-C38	120.0452	C19-C25-H26	109.4580	C9-C13-N44	119.9708
C34-C36- H41	119.9742	C18-C19-C29	111.6782	C9-C13-C11	120.0561
C37-C34-C33	120.0114	C19-C29-H30	109.4636	C13-N44-O46	120.0203
C33-C35-H39	119.9958	C19-C29-H31	109.4361	C13-N44-O45	120.0251
C35-C38-H42	119.9895	C19-C29-H32	109.4958	C11-C13-N24	119.9729
C33-C5-S24	123.4910	C18-C16-O20	121.5475	C11-C13- H15	120.0461
C33-C5-N23	123.4724	C18-C16-C3	116.9998	C11-C8- H15	119.9962
S24-C1-N22	107.9175	C16-C3-C2	120.8400	C8-C11-C13	119.9575
N22-C1-N21	125.9453	C3-C16-O20	121.3858	C13-C8-C6	119.9955
C1-N21-C2	114.1350	C3-C4-C6	120.8496	C8-C11- H12	120.0232
C2-C3-C4	115.6292	C4-C6-C7	119.9665	O45-N14-O46	119.9545
N22-C4-C3	118.5722	C4-C6-C8	119.9944	C1-S24-C5	87.5424
C3-C2-C17	119.9576	C6-C7-H10	120.0320	S24-C5-N23	113.0305

C2-C17-C19	119.4817	C6-C8-H12	119.9818	N23-N22-C1	105.8200
C17-C19-C18	105.4731	C10-C7-C9	119.9620	N23-N22-C4	117.8311
C17-C19-C25	109.2021	C7-C9-H14	120.0235		
C19-C25-H28	109.4529	C7-C9-C13	119.9457		

…………S3………………………

Table : Optimized Bond Length and Bond Angles Calculated at the 6-311++G(d,p) level of V2 molecule

Bond lengths (Å)					
C1-S25	1.743	**C13-Cl16**	**1.760**	C30-H33	1.070
C1-N22	1.342	C13-C11	1.401	C5-C34	1.540
C1-N23	1.474	C11-H15	1.070	C5-S25	1.777
C2-N22	1.486	C11-C8	1.401	C5-N24	1.324
C2-C3	1.344	C8-H12	1.070	N23-N24	1.426
C2-C18	1.456	C17-O21	1.258	C34-C35	1.401
C3-C4	1.520	C17-C19	1.533	C35-C37	1.401
C3-C17	1.531	C19-C20	1.547	C35-H38	1.070
C4-C6	1.540	C20-C19	1.547	C37-H42	1.070
C4-N23	1.445	C20-C26	1.540	C37-C41	1.401
C6-C7	1.401	C26-H27	1.070	C41-C44	1.070
C6-C8	1.401	C26-H28	1.070	C41-C39	1.401
C7-H10	1.070	C26-H29	1.070	C39-H43	1.070
C9-H14	1.070	C20-C30	1.540	C39-C36	1.401
C7-C9	1.401	C30-H31	1.070	C36-H40	1.070
C9-C13	1.401	C30-H32	1.070	C34-C36	1.401
Bond angles (Å)					
C37-C41- H44	120.0000	C2-C3-C4	115.6221	C3-C4-C6	120.8511
C41-C39- H44	120.0000	N24-N23-C4	117.8013	C4-C6-C7	120.0000
C41-C37-H42	120.0000	C4-C3-C17	123.4932	C4-C6-C8	120.0000
C35- C37- H42	120.0000	C3-C17-O21	121.3898	C6-C7-H10	120.0000
C37-C35-H38	120.0000	C3-C2-C18	119.9912	C6-C8-H12	120.0000
C35-C34- H38	120.0000	C2-C18-C20	119.4741	C6-C7-C9	120.0000
C34-C36-H40	120.0000	C18-C20-C26	109.2308	C7-C9-H14	120.0000
C35-C34-C5	120.0000	C20-C26-H27	109.4712	C18-C7-C9	120.0000
C34-C5-S25	123.4923	C20-C26-H28	109.4712	C9-C7- H14	120.0000
C34-C5-N24	123.4823	C20-C26-H29	109.4712	C9-C13- H14	120.0000
N24-N23-C1	105.8150	C3-C19-C20	116.9801	C9-C13-Cl16	120.0000
C5-S25-C1	87.5185	C17-C19-C20	115.7592	C9-C13-C11	120.0000
S25-C1-N22	126.0858	C19-C20-C30	111.6869	C13-C11-H15	120.0000
S25-C1-N23	107.9345	C18-C20-C30	112.0490	C14-C9-H13	120.0000
C1-N22-C2	114.1473	C20-C30-H31	109.4712	C11-C13-Cl16	120.0000
N22-C1-N23	125.9251	C20-C30-H32	109.4712	C11-C8- H15	120.0000
N22-C2-C3	117.5025	C20-C30-H33	109.4712	C8-C11- H12	120.0000

…………S4………………………

Table : Optimized Bond Length and Bond Angles Calculated at the 6-311++G(d,p) level of V5 molecule

Bond lengths (Å)					
C1-S23	1.743	C13-C11	1.401	C28-H31	1.070
C1-N20	1.324	C11-C8	1.401	C5-S23	1.777
C1-N21	1.474	**C11-O44**	**1.430**	C5-N22	1.324
C2-C3	1.344	**O44-H45**	**0.960**	C5-C32	1.540
C2-N20	1.486	C8-H22	1.070	C32-C33	1.401
C3-C4	1.520	C15-O19	1.258	C33-C35	1.401
C3-C15	1.531	C15-C17	1.533	C33-H36	1.070
C4-N21	1.445	C17-C18	1.547	C35-H40	1.070
C4-C6	1.540	C18-C16	1.544	C35-C39	1.401
C6-C7	1.401	C18-C24	1.540	C39-H42	1.070
C6-C8	1.401	C18-C28	1.540	C39-C37	1.401
C7-H10	1.071	C24-H25	1.070	C37-H41	1.070
C7-C9	1.401	C24-H26	1.070	C37-C34	1.401
C9-H14	1.070	C24-H27	1.070	C34-H38	1.070
C9-C13	1.401	C28-H29	1.070	C34-C32	1.401
**C13-H43**	**1.070**	C28-H30	1.070		
Bond angles (Å)					
C42-C39-C37	119.9999	N21-C4-C3	118.5661	C18-C28-H31	109.4712
C35- C39- H42	120.0000	N21-C4-C6	120.4491	C17-C15-O19	121.5630
C39-C37-H41	120.0000	N20-C2-C3	117.5025	C15-C3-C4	123.9132
C39-C35-H40	120.0000	N20-C2-C16	122.4493	C3-C4-C6	120.8511
C34-C37- H41	119.9999	C2-C3-C15	120.8137	C4-C6-C7	120.0000
C37-C34-H38	119.9999	C3-C15-O19	121.3898	C4-C6-C8	119.9999
C32-C34- H38	120.0000	C3-C15-C17	116.9801	C6-C7-H10	120.0000
C35-C33-H36	120.0000	O9-C15-C17	121.5630	C6-C8-H12	119.9999
C33-C35- H40	120.0000	C15-C17-C18	115.7992	C6-C7-C9	119.9999
H32-C33- H36	119.9999	C2-C16-C18	119.4241	C9-C7- H10	120.0000
C32-C5-S23	123.4924	C16-C18-C24	109.2308	C7-C9-H14	120.0000
C32-C5-N22	123.4823	C17-C18-C24	109.5931	C13-C9- H14	119.9999
C5-S23-C1	87.5185	C18-C24-H25	109.4712	C9-C13-H43	119.9999
C5-N22-N21	113.3482	C18-C24-C26	109.4712	C9-C13-C11	120.0000
N22-N21-C1	105.8150	C18-C24-C27	109.4712	C11-C13- H43	120.0000
S23-C1-N21	107.9345	C17-C18-C28	111.6869	C13-C11-O44	120.0000
S23-C1-N20	126.0857	C16-C18-C28	112.0490	C11-O44-H45	119.4712
N21-C1-N20	125.9251	C18-C28-H29	109.4712	C11-C13-H43	120.0000
N22-N21-C4	117.8013	C18-C28-H30	109.4712		

Table : Optimized Bond Length and Bond Angles Calculated at the 6-311++G(d,p) level of V7 molecule

Bond lengths (Å)					
C1-S23	1.743	C8-H12	1.070	C18-C28	1.540

C1-N20	1.324	**N44-C45**	**1.470**	C28-H29	1.070
C1-N21	1.474	**N44-C46**	**1.470**	C28-H30	1070
C2-C3	1.344	C45-H47	1.070	C28-H31	1070
C2-C16	1.546	C45-H48	1.070	C5-C32	1.540
C3-C4	1.520	C45-H49	1.070	C5-S23	1.777
C4-C6	1.540	C46-H50	1.070	C5-N22	1.324
C4-N21	1.445	C46-H51	1.070	C32-C33	1.401
C6-C7	1.401	C46-H52	1.070	C32-C34	1.401
C6-C8	1.401	C3-C15	1.531	C33-H36	1.070
C7-H10	1.070	C15-O19	1.258	C33-C35	1.401
C7-C9	1.401	C15-C17	1.533	C35-H40	1.070
C9-H14	1.070	C17-C18	1.547	C35-C39	1.401
C9-C13	1.401	C18-C16	1544	C39-H42	1.070
**C13-N44**	**1.470**	C18-C24	1.540	C39-C37	1.402
C13-C11	1.401	C24-H25	1.070	C37-H41	1.070
C11-H43	1.070	C24-H26	1.070	C37-C34	1401
C11-C8	1.401	C24-H27	1.070	C34-H38	1.070
Bond angles (Å)					
C37-C39- H42	119.9981	N21-C4-C3	118.5381	C8-C11-H43	120.0010
C35-C39- H42	119.9987	N20-C2-C16	122.4368	C13-C11- H43	119.9930
C39-C37-H41	120.0040	C2-C16-C18	119.5152	C13-C9-H14	120.0086
C41-C37-C34	119.9986	C2-C3-C15	120.7988	C11-C13-H24	119.9600
C37-C34-H38	120.0103	C3-C15-O19	121.3894	C9-C13-N44	120.0255
C32-C35- H38	119.9989	C3-C15-C17	116.9923	C13-N44-C45	117.4917
C33-C35- H40	120.0013	C16-C18-C24	107.2202	N44-C45-H47	109.4729
C35-C33-H36	120.0019	C18-C24-H27	109.5637	N44-C45-H48	109.4622
C32-C33-H36	120.0006	C18-C24-H26	109.4783	N44-C45-H49	109.4776
C33-C32-C5	119.9733	C18-C24-H25	109.4715	N44-C45-H50	109.4848
C34-C32-C5	120.0085	C17-C18-C28	111.6915	N44-C46-H51	109.4688
C32-C5-S23	123.5352	C18-C28-H29	109.4640	N44-C46-H52	109.4592
C32-C5-N22	123.4333	C18-C28-H30	109.4733	H50-C46-H52	109.4678
C5-N22-N21	113.3512	C18-C28-H31	109.4784	H52-C46-H51	109.4707
C5-S23-C1	87.4939	C4-C6-C7	120.0330	H47-C45-H49	109.4607
C23-C1-N21	107.9331	C4-C6-C8	119.9626	H48-C45-H47	109.4805
N22-N21-C1	105.8232	C6-C7-C10	120.0038	H49-C45-H47	109.4607
C23-C1-N20	126.0848	C6-C8-H12	120.0029	C13-N44-C46	109.1767
N22-N21-C4	117.7502	C6-C7-C9	119.9968	C13-N44-C45	117.4917
C1-N21-C4	107.1453	C6-C8-C11	119.9979	C45-N44-C46	109.2491
C1-N20-C2	114.0955	C7-C9-H14	120.0069	S23-C1-N20	126.0848
N20-C2-C3	117.5042	C7-C9-C13	119.9843	S23-C1-N21	107.9331

General procedure for the synthesis of thiadiazolo quinazolinones derivatives (V1-V8):

The condensation of 2-amino 5-phenyl 1,3,4-thiadiazole (S) (1mmol, 0.177g), dimedone (U) (1mmol, 0.140 g), and different substituted aromatic aldehydes (T1-7)

(1mmol) were added to 2.5 ml water and 2.5 ml ethanol in the presence of magnetic WFe_2O_4/rGO nanocomposite catalyst (15 mg) in 100ml round bottom flask. At room temp, the reaction was continually stirred. Thin-layer chromatography (TLC) in an ethyl acetate and petroleum ether (20:80, v/v) solvent solution was used to monitor the reaction's progress. Upon completion of the reaction, the nanocatalyst was separated by applying a strong external magnet, and the corresponding crude product was filtered and recrystallized from hot ethanol to afford the desired thiadiazolo [2,3-*b*] quinazoline-6-ones derivatives **(V1-8)**

……………………………………….S7-S14…………………………………

<table>
<tr><td colspan="2">5-(4-nitrophenyl)-8,9-dihydro-8,8-dimethyl-2-phenyl-5H-[1,3,4] thiadiazolo[2,3-b]quinazolin-6(7H)-one(V-1)</td></tr>
<tr><td>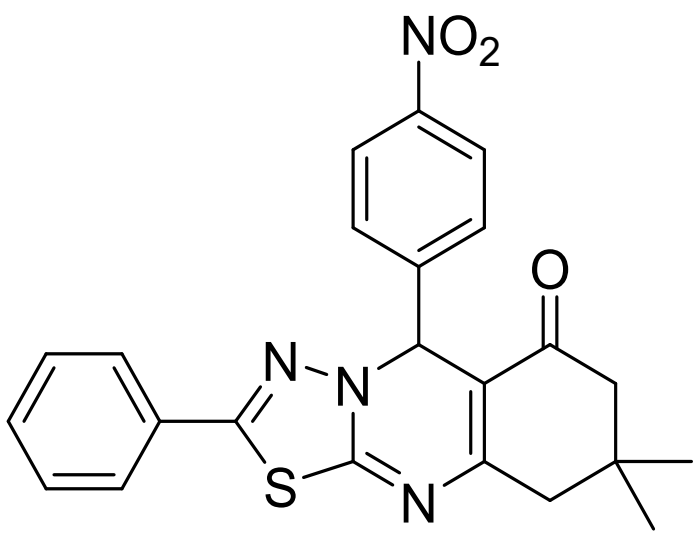</td><td>M.P. 205^0C, Yield 89%, M.F. $C_{23}H_{20}O_3N_4S$; IR (KBr/cm^{-1}): 3374, 3088, 2958, 1721, 1516, 1382, 1232 ; ^{1}H NMR (400 MHz, DMSO–d_6): δ (ppm) 7.5- 7.8 (m, 4H), 7.0- 7.3 (m, 5H), 6.2 (s, 1H, -CH), 2.4 (s, 2H -CH_2), 2.1 (s, 2H -CH_2), 1.1 (s, 3H,- CH_3), 0.8 (s, 3H,- CH_3); ^{13}C NMR(300 MHz, DMSO–d_6): δ (ppm) 204.32, 196.06, 187.25, 168.93, 157.30, 129.63, 124.15, 113.89, 109.78, 56.59, 50.78, 28.26, 29.72, 26.49; EI-MS (m/z: RA%): [M+4] 436 (m/z: 100%).</td></tr>
<tr><td colspan="2">5-(4-chlorophenyl)-8,9-dihydro-8,8-dimethyl-2-phenyl-5H-[1,3,4]thiadiazolo[2,3-b]quinazolin-6(7H)-one(V-2)</td></tr>
<tr><td>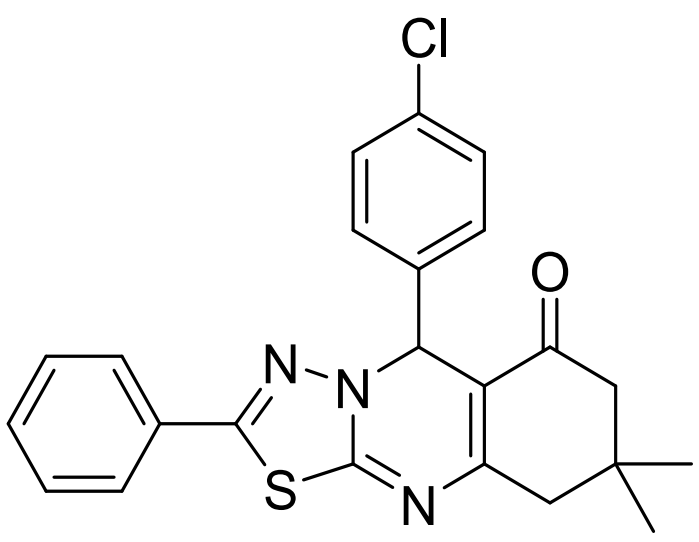</td><td>M.P. 213^0C, Yield 86%, M.F. $C_{23}H_{20}ON_3SCl$; IR (KBr/cm^{-1}): 3374, 3088, 2958, 1721, 1516, 1382, 1232; ^{1}H NMR (400 MHz, DMSO–d_6): δ (ppm) 7.9- 7.3 (m, 4H), 7-7.3 (m, 5H), 5.7 (s, 1H, -CH), 2.2 (s, 2H -CH_2), 2.5 (s, 2H -CH_2), 1.1 (s, 3H- CH_3), 0.9 (s, 3H- CH_3); ^{13}C NMR (100 MHz, DMSO–d_6): δ (ppm) 197.26, 181.95, 168.93, 140.29, 131.36, 133.32, 129.64, 126.08, 112.42, 56.58, 49.50, 40.40,31.40, 28.26; EI-MS (m/z: RA%): 421 (m/z: 100%).</td></tr>
<tr><td colspan="2">5-(3-nitrophenyl)8,9-dihydro-8,8-dimethyl-2-phenyl-5H-[1,3,4] thiadiazolo[2,3-b]quinazolin-6(7H)-one (V-3)</td></tr>
<tr><td>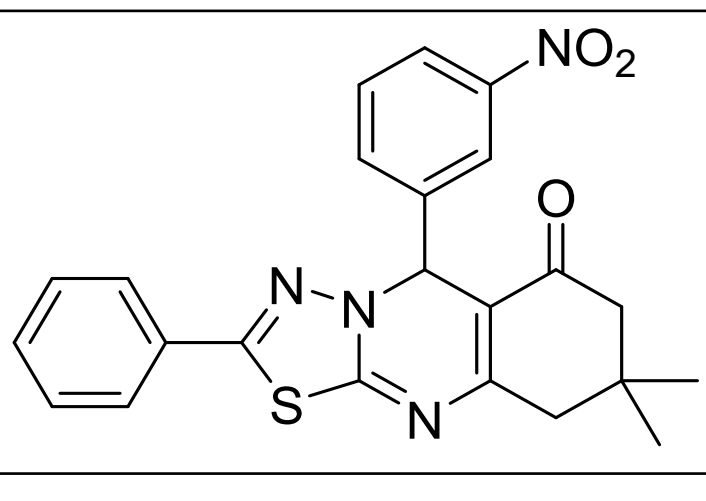</td><td>M.P. 209^0C, Yield 87%, M.F. $C_{23}H_{20}O_3N_4S$; IR (KBr/cm^{-1}): 3264, 3088, 3061, 1632, 1593, 1511, 1376, 1249; ^{1}H NMR (400 MHz, DMSO–d_6): δ (ppm) 7.0- 7.4 (m, 5H) 7.5-8.2 (m, 4H), 6.04 (s, 1H, -CH), 2.51 (s, 2H -CH_2), 2.11 (s, 2H -CH_2), 1.15 (s, 3H,- CH_3), 0.97 (s, 3H,- CH_3); ^{13}C NMR(100 MHz, DMSO–d_6): δ (ppm)190.40,</td></tr>
</table>

	184.50, 172.37, 169.17, 143.72, 150.97, 128.13, 124.73, 117.12, 109.69, 41.79, 26.48, 23.51 : EI-MS (m/z: RA%): [M+4] 436 (m/z: 100%).
**5-(2-nitrophenyl)8,9-dihydro-8,8-dimethyl-2-phenyl-5H-[1,3,4]thiadiazolo[2,3-b] quinazolin -6(7H)-one(V-4)**	
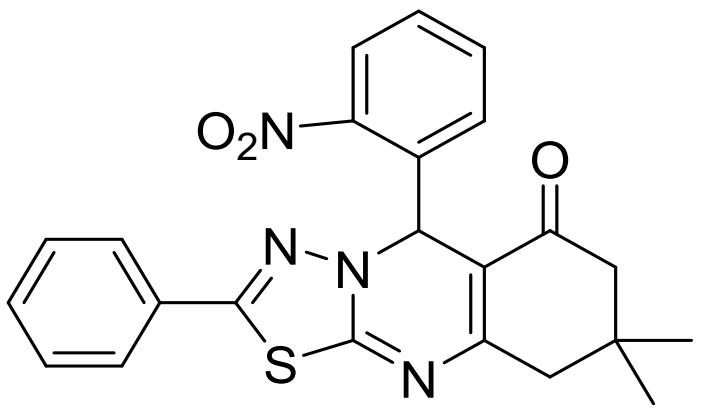	M.P. 216^{0}C, Yield 86%, M.F. $C_{23}H_{20}O_3N_4S$; IR (KBr/cm^{-1}): 3371, 3280, 3088, 2959, 1739, 1518, 1383, 1232;. ^{1}H NMR (400 MHz, DMSO–d$_6$): δ (ppm) 7.2-8.1 (m, 4H), 7.0-7.2 (m, 5H), 6.34 (s, 1H, -CH), 2.27 (s, 2H -CH_2), 2.3 (s, 2H -CH_2), 1.18 (s, 3H,- CH_3), 0.95 (s, 3H,- CH_3); ^{13}C NMR(100 MHz, DMSO–d$_6$): δ (ppm) 204.55, 168.94, 157.09, 132.72, 131.42, 113.83, 101.50, 56.61, 42.79, 46.97, 33.14, 28.27, 26.54. ppm: : EI-MS (m/z: RA%): [M+4] 436 (m/z: 100%).
**5-(3-hydroxylphenyl)8,9-dihydro-8,8-dimethyl-2-phenyl-5H-[1,3,4] thiadiazolo[2,3-b] quinazolin-6(7H)-one(V-5)**	
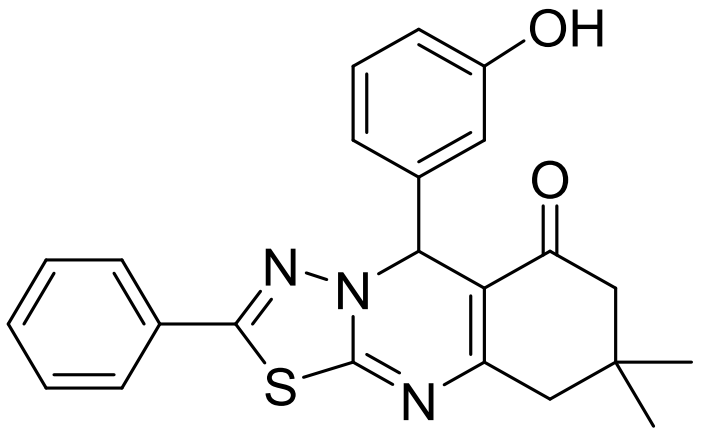	M.P. 218^{0}C, Yield 80%, M.F. $C_{22}H_{21}O_2N_3S$; IR (KBr/cm^{-1}): 3272, 3072, 2959, 1592, 1513, 1468, 1139 cm^{-1}. ^{1}H NMR (400 MHz, DMSO–d$_6$): δ (ppm) 7.0-7.81 (m, 4H), 7.00-6.60 (m, 5H), 6.43 (s, 1H, -CH), 8.9 (s, -OH), 2.70 (s, 2H -CH_2), 2.27 (s, 2H -CH_2), 1.09 (s, 3H,- CH_3), 1.01 (s, 3H,- CH_3); ^{13}C NMR(100 MHz, DMSO–d$_6$): δ (ppm) 168.94, 157.35, 131.35, 129.63, 126.57, 115.25, 78.55, 47.18, 31.34; : EI-MS (m/z: RA%): 391 (m/z: 100%).
**5-(4-hydroxy-3-methoxyphenyl)8,9-dihydro-8,8-dimethyl-2-phenyl-5H-[1,3,4] thiadiazolo[2,3-b]quinazolin-6(7H)-one(V-6)**	
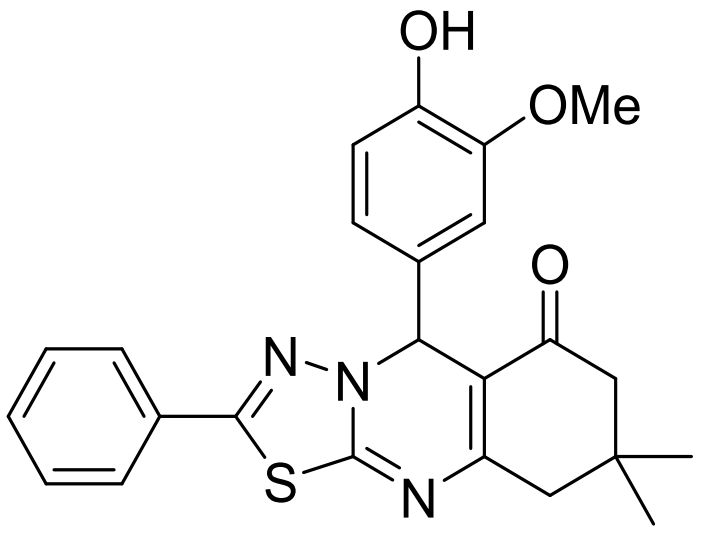	M.P. 217^{0}C, Yield 82%, M.F. $C_{24}H_{23}O_3N_3S$; IR (KBr/cm^{-1}): 3269, 3058, 2957, 1632, 1588, 1512, 1467, 1238 cm^{-1} 2957; ^{1}H NMR (400 MHz, DMSO–d$_6$): δ (ppm) 8.11-8.33 (m, 5H), 6.51 -7.5 (m, 3H), 8.54 (s, -1H -OH), 5.60 (s, 1H, -CH), 3.83 (s, 3H, -OCH_3), 2.29(s, 2H -CH_2), 2.13 (s, 2H -CH_2), 1.20 (s, 3H,- CH_3), 0.96 (s, 3H,- CH_3); ^{13}C NMR(100 MHz, DMSO–d$_6$): δ (ppm) 189.06, 168.90, 144.33, 147.34, 126.57, 129.03, 131.39, 129.59, 115.15, 115.57, 110.81, 55.60, 46.60, 46.77, 31.80, 31.31 ; : EI-MS (m/z: RA%): [M+4] 437. (m/z: 100%).
**5-(4-N,Ndimethylphenyl)8,9-dihydro-8,8-dimethyl-2-phenyl-5H-[1,3,4]thiadiazolo[2,3-b]quinazolin-6(7H)-one(V-7)**	

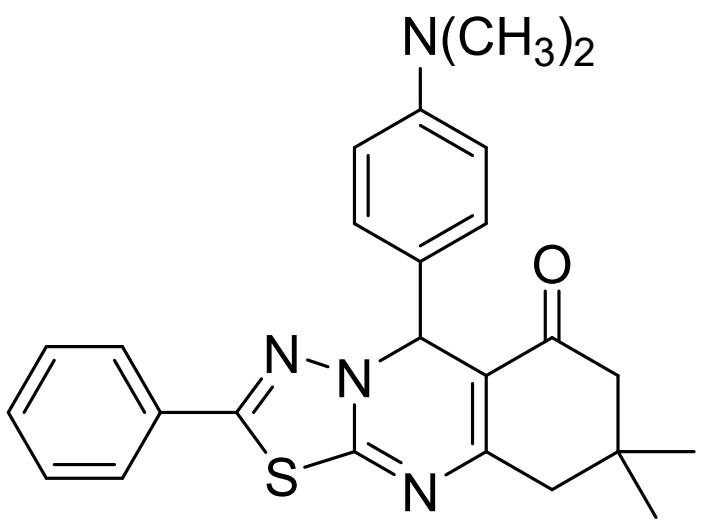	M.P. 205^{0}C, Yield 81%, M.F. $C_{24}H_{26}N_4OS$; IR (KBr/cm^{-1}): 3088, 2958, 1721, 1530, 1362, 1232 ; ^{1}H NMR (400 MHz, DMSO–d$_6$): δ (ppm) 7.20 - 7.30 (m, 4H), 7-7.50 (m, 5H), 6.11 (s, 1H, -CH), 2.35 (s, 6H), 2.25 (s, 2H -CH_2), 2.08 (s, 2H -CH_2), 1.17 (s, 3H,- CH_3), 0.96 (s, 3H,- CH_3); ^{13}C NMR(100 MHz, DMSO–d$_6$): δ (ppm) 195.26, 140.29, 131.36, 129.64, 126.08, 112.42, 55.55, 45.40, ,40.40, 31.40, 28.26 EI-MS (m/z: RA%): 430 (m/z: 100%).
**5-(3,4-dimethoxyphenyl)8,9-dihydro-8,8-dimethyl-2-phenyl-5H-[1,3,4] thiadiazolo[2,3-b]quinazolin-6(7H)-one(V-8)**	
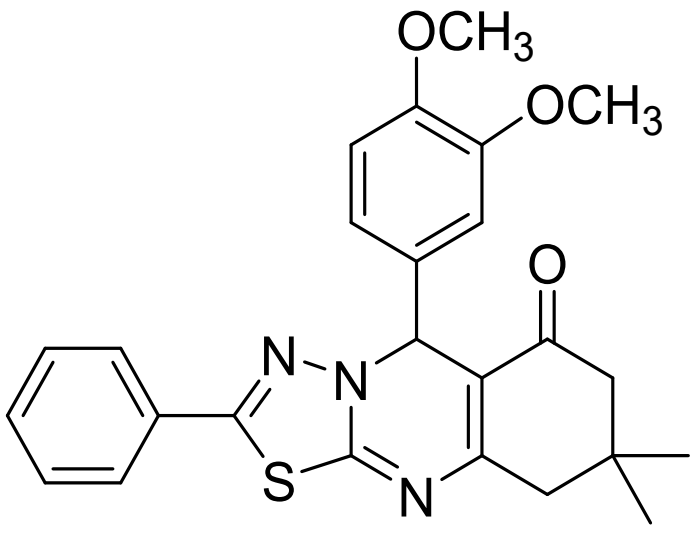	M.P. 193^{0}C, Yield 80%, M.F. $C_{25}H_{25}N_3O_3S$; IR-3058, 2957, 1632,1467, 1125, 1238 cm^{-1} ^{1}H NMR (400 MHz, DMSO–d$_6$): δ (ppm) 7.54 - 7.26 (m, 5H), 7-6.5 (m, 3H), 6.35 (s, 1H, -CH), 3.73 (s, 3H, -OCH_3), 3.83 (s, 3H, -OCH_3), 2.19 (s, 2H -CH_2), 2.13 (s, 2H -CH_2), 1.20 (s, 3H,- CH_3), 0.83 (s, 3H,- CH_3); ^{13}C NMR(100 MHz, DMSO–d$_6$): δ (ppm) 189.06, 168.90,144.33, 147.34, 126.57, 131.39, 129.59, 115.15, 55.60, 46.60, 46.77, 31.80, 31.31 ; EI-MS (m/z: RA%): 446 (m/z: 100%).

……………………………..S15-S37……………………………….

Some spectra of Representative compounds:

IR Spectrum of Compound (V-1): S15

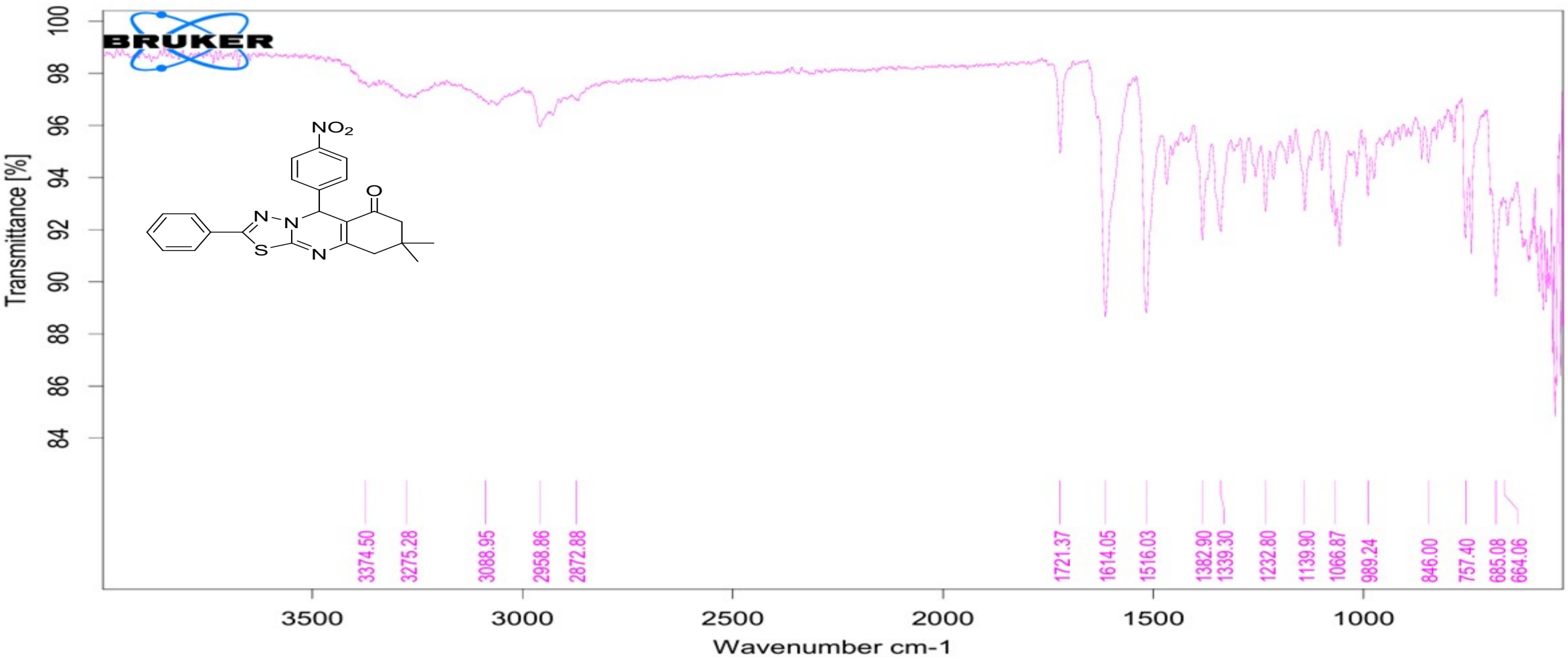

The IR Spectrum of 5-(4-nitrophenyl)-8,9-dihydro-8,8-dimethyl-2-phenyl-5H-[1,3,4] thiadiazolo[2,3-b]quinazolin-6(7H)-one.

^{1}H NMR Spectrum of Compound (V-1) S16

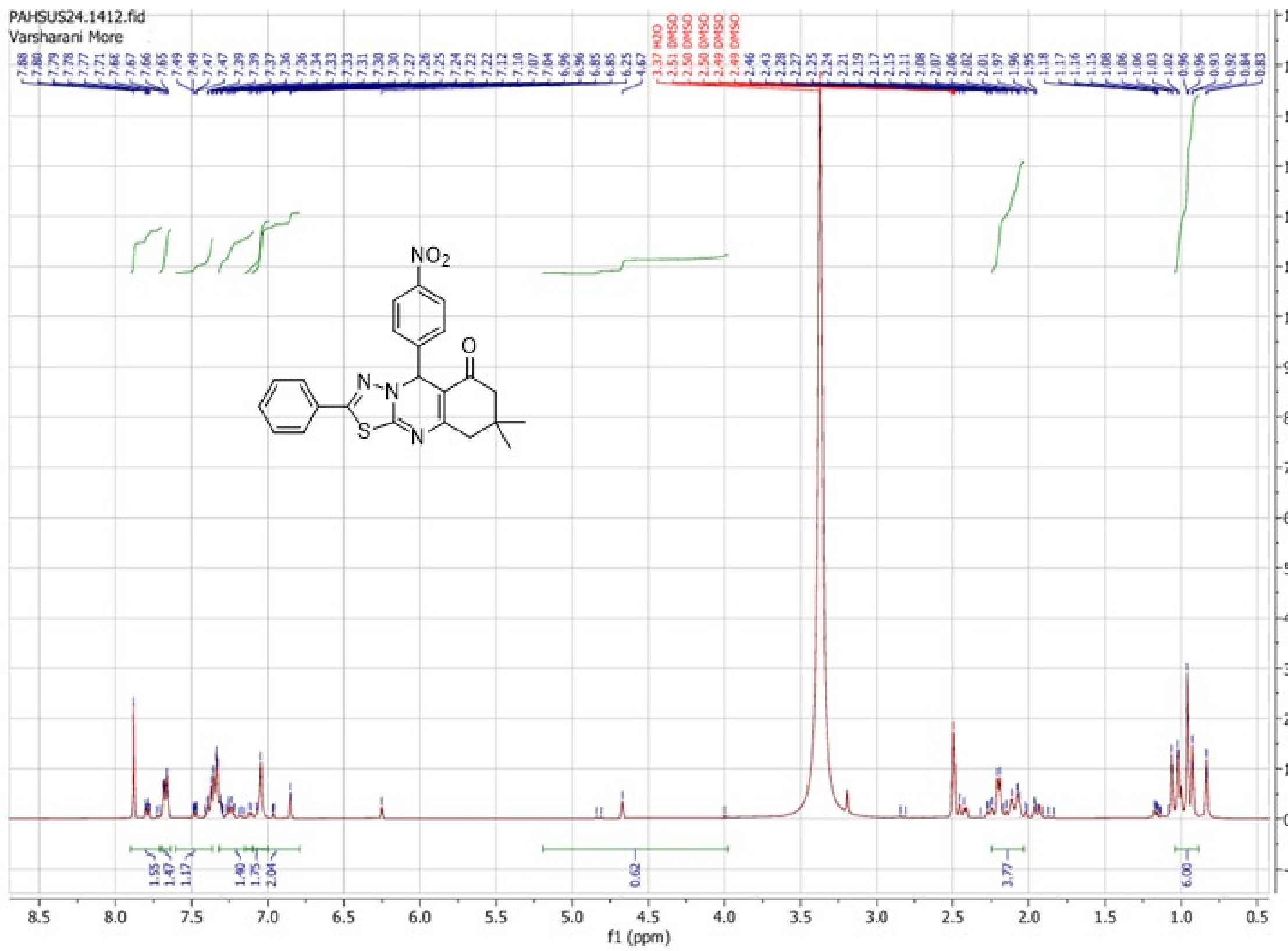

The ^{1}H NMR of 5-(4-nitrophenyl)-8,9-dihydro-8,8-dimethyl-2-phenyl-5H-[1,3,4] thiadiazolo[2,3-b]quinazolin-6(7H)-one.

^{13}C NMR Spectrum of Compound (V-1) S17

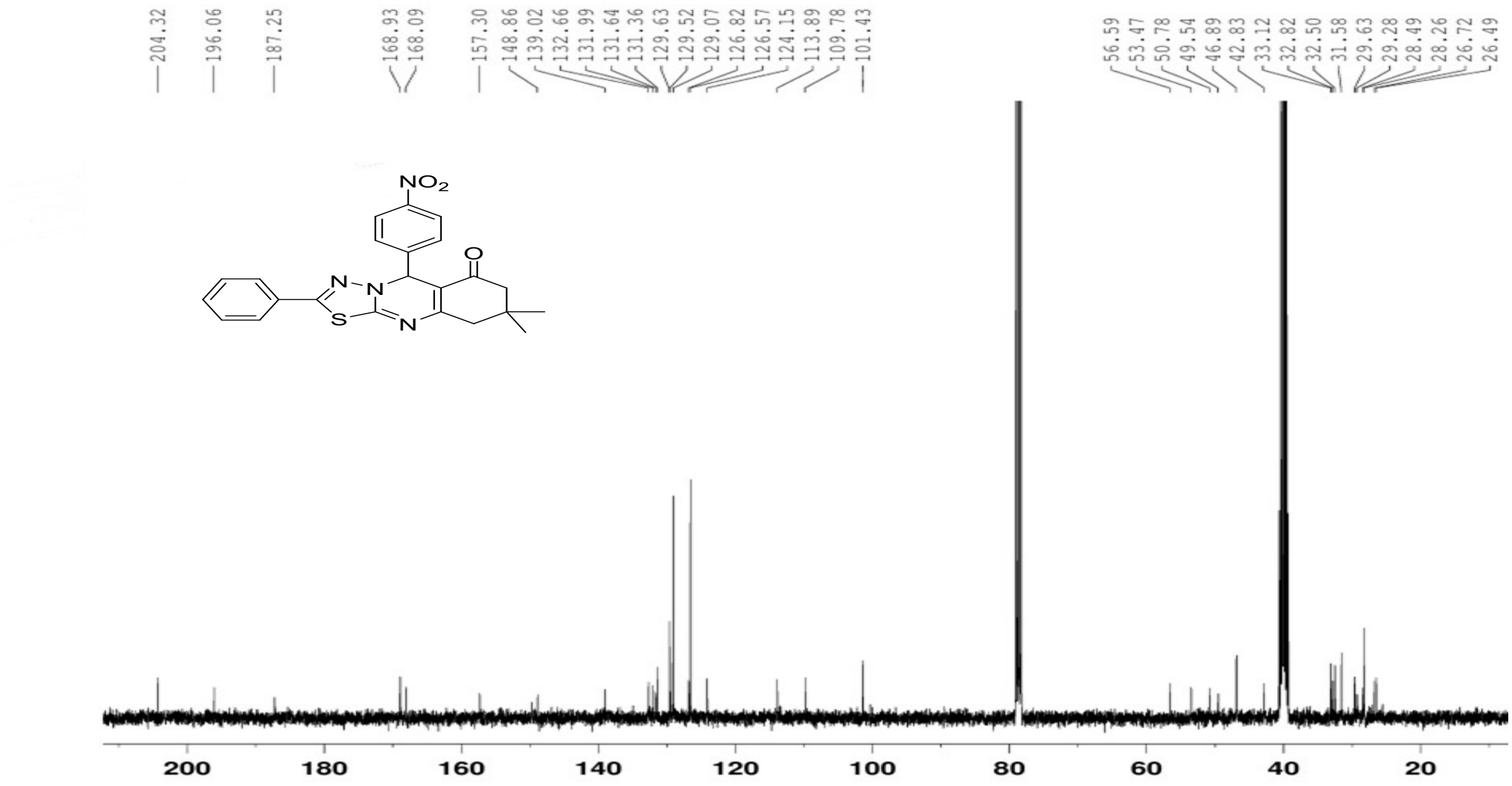

The ^{13}C NMR of 5-(4-nitrophenyl)-8,9-dihydro-8,8-dimethyl-2-phenyl-5H-[1,3,4] thiadiazolo[2,3-b]quinazolin-6(7H)-one.

Mass Spectrum of Compound (V-1) S18

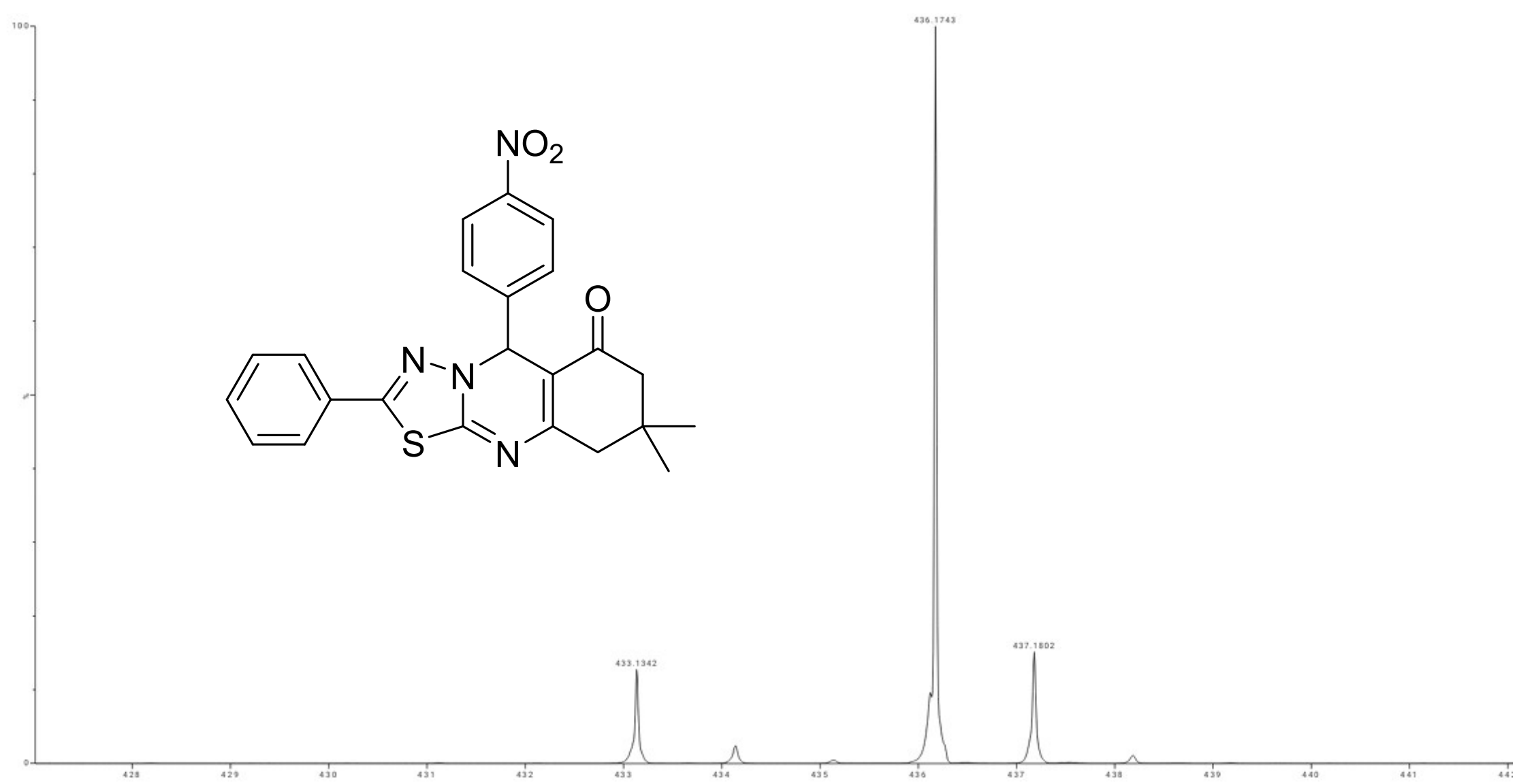

The Mass spectrum of 5-(4-nitrophenyl)-8,9-dihydro-8,8-dimethyl-2-phenyl-5H-[1,3,4] thiadiazolo[2,3-b]quinazolin-6(7H)-one.

IR Spectrum of Compound (V2) S19

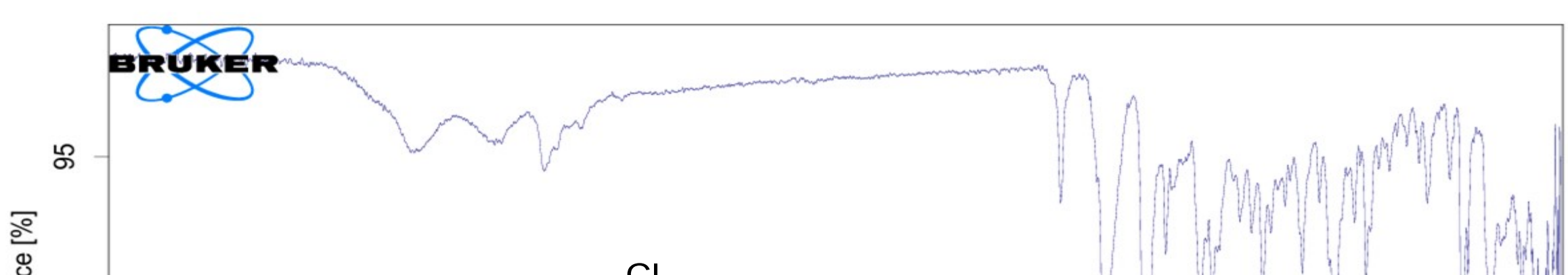

^{1}H NMR Spectrum of Compound (V2) S20

The IR Spectrum of 5-(4-chlorophenyl)-8,9-dihydro-8,8-dimethyl-2-phenyl-5H-[1,3,4]thiadiazolo[2,3-b]quinazolin-6(7H)-one.

^{1}H NMR Spectrum of Compound (V-2) S20

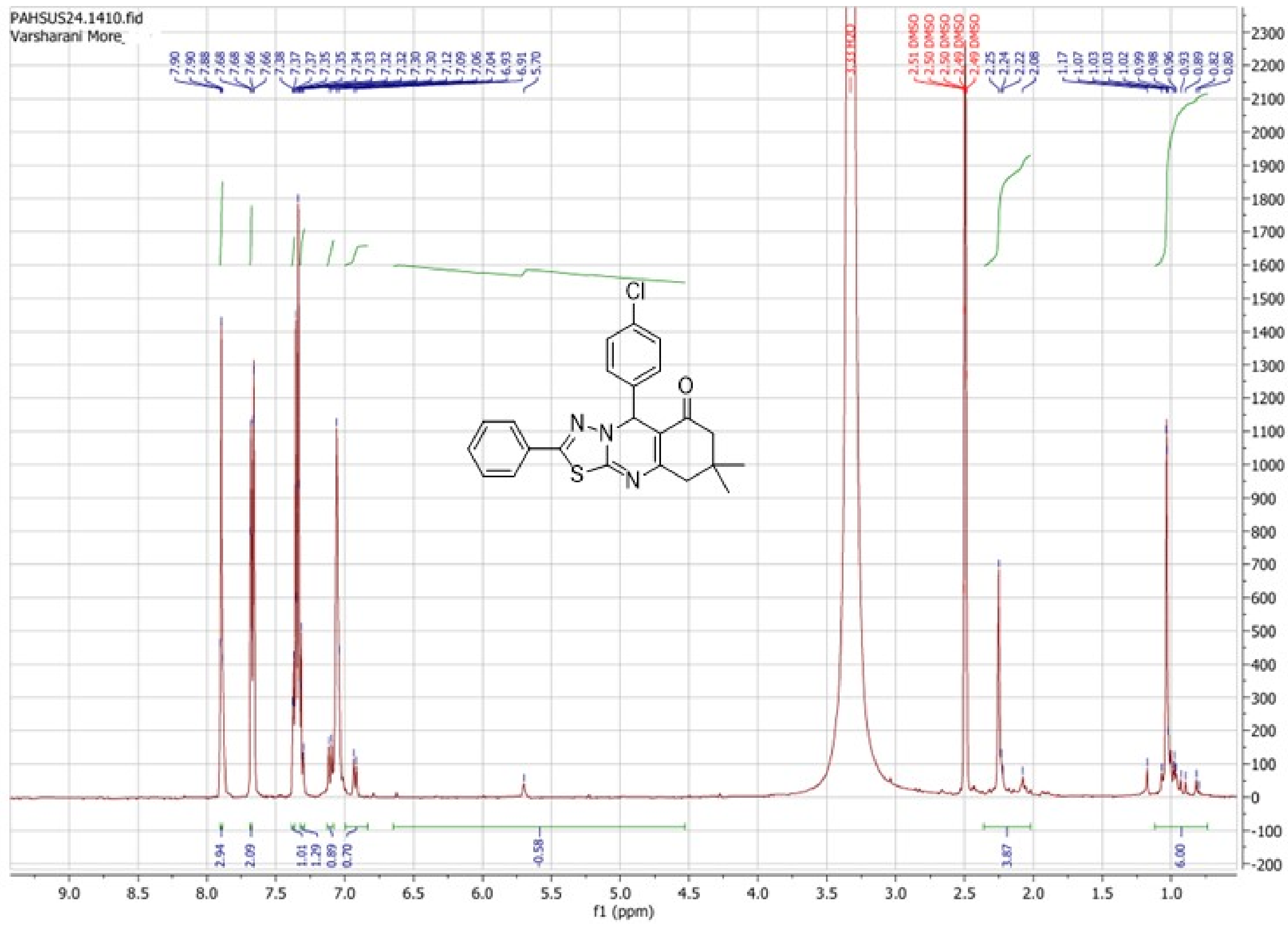

The ^{1}H NMR Spectrum of 5-(4-chlorophenyl)-8,9-dihydro-8,8-dimethyl-2-phenyl-5H-[1,3,4]thiadiazolo[2,3-b]quinazolin-6(7H)-one.

^{13}C NMR Spectrum of Compound (V2) S21

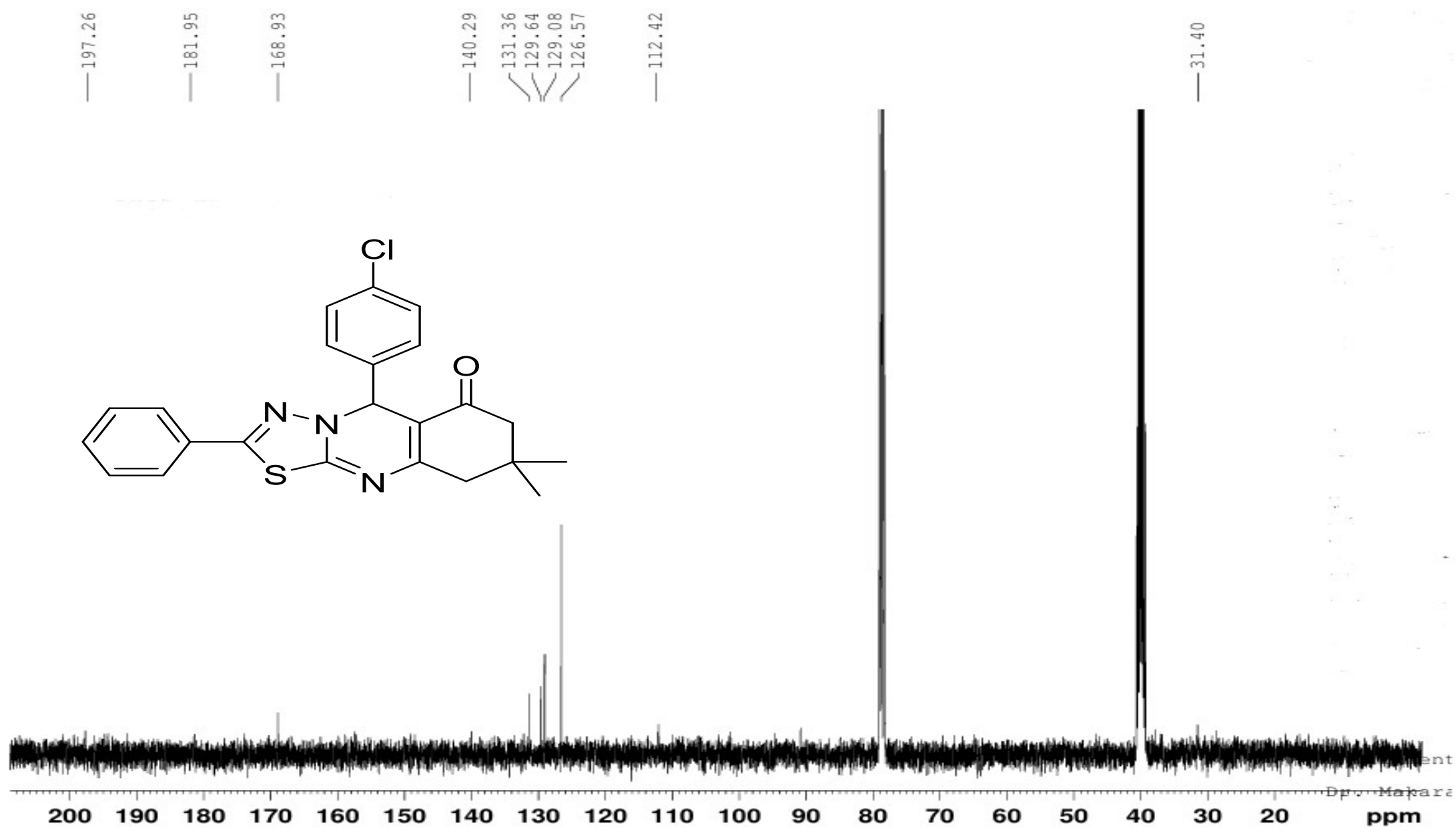

The ^{13}C NMR Spectrum of 5-(4-chlorophenyl)-8,9-dihydro-8,8-dimethyl-2-phenyl-5H-[1,3,4]thiadiazolo[2,3-b]quinazolin-6(7H)-one.

IR Spectrum of Compound (V-3) S22

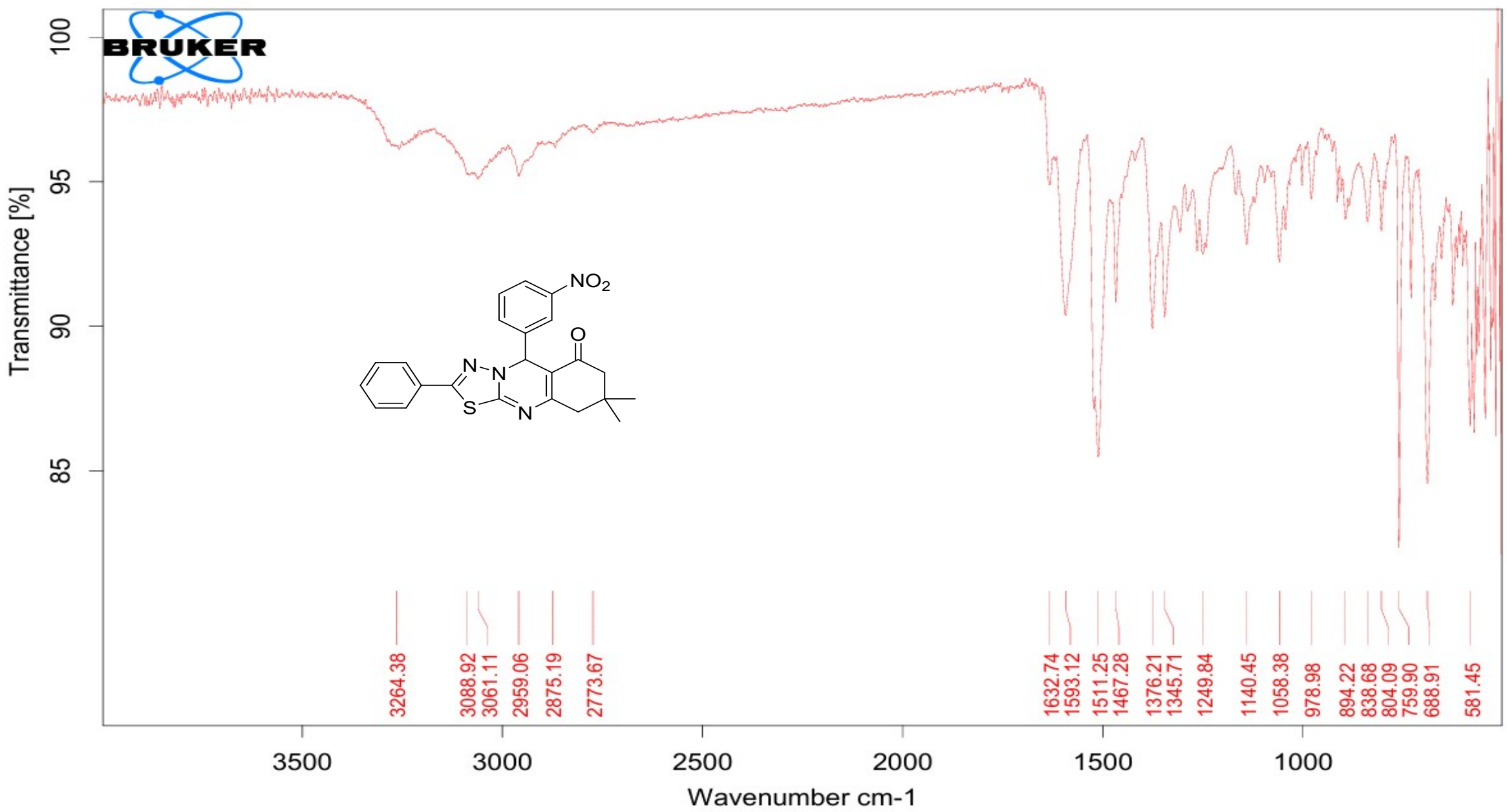

The IR Spectrum of 5-(3-nitrophenyl)-8,9-dihydro-8,8-dimethyl-2-phenyl-5H-[1,3,4] thiadiazolo[2,3-b]quinazolin-6(7H)-one.

^{1}H NMR Spectrum of Compound (V-3) S23

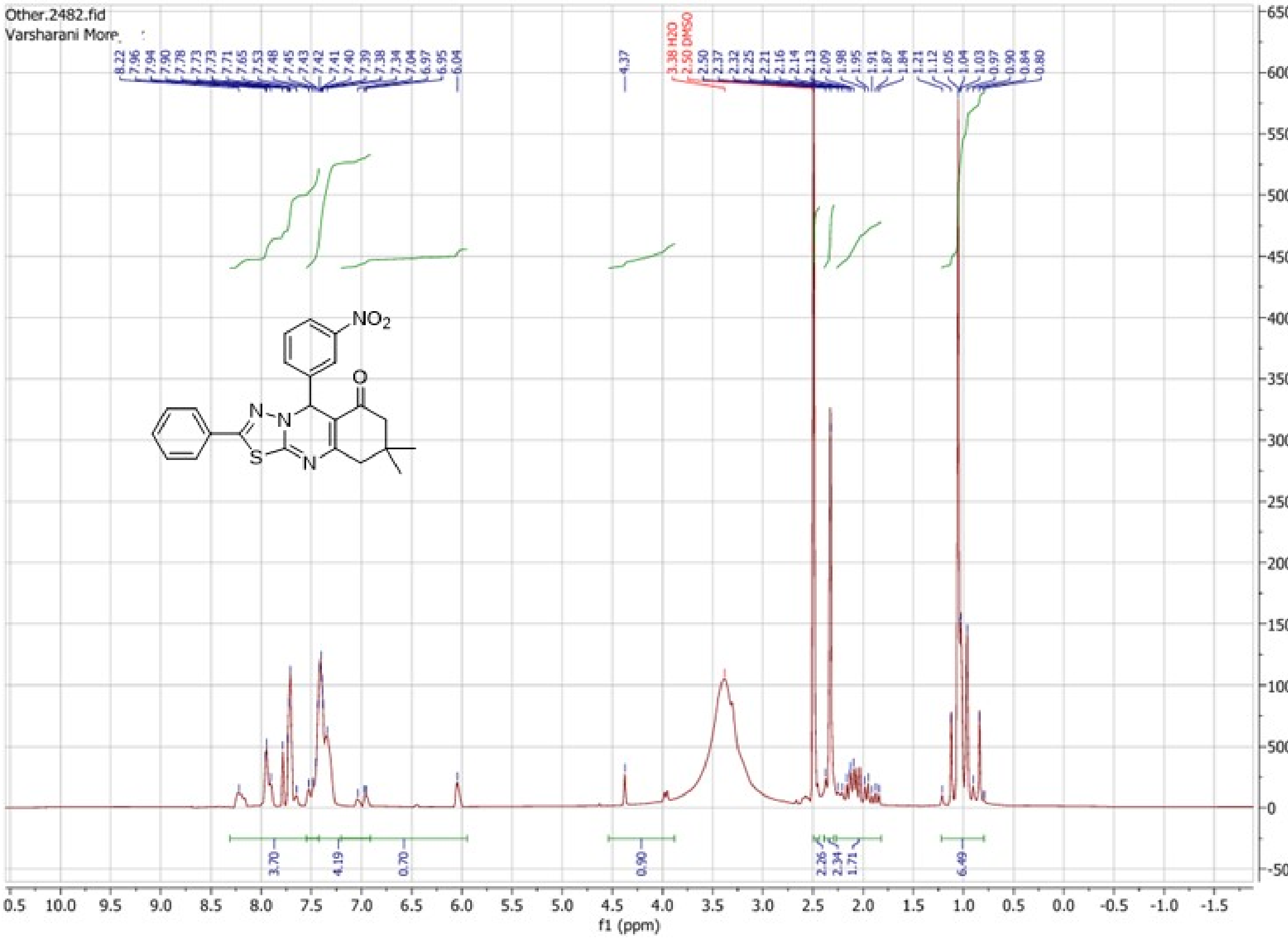

The ^{1}H NMR Spectrum of 5-(3-nitrophenyl)-8,9-dihydro-8,8-dimethyl-2-phenyl-5H-[1,3,4] thiadiazolo[2,3-b]quinazolin-6(7H)-one.

^{13}C NMR Spectrum of Compound (V-3) S24

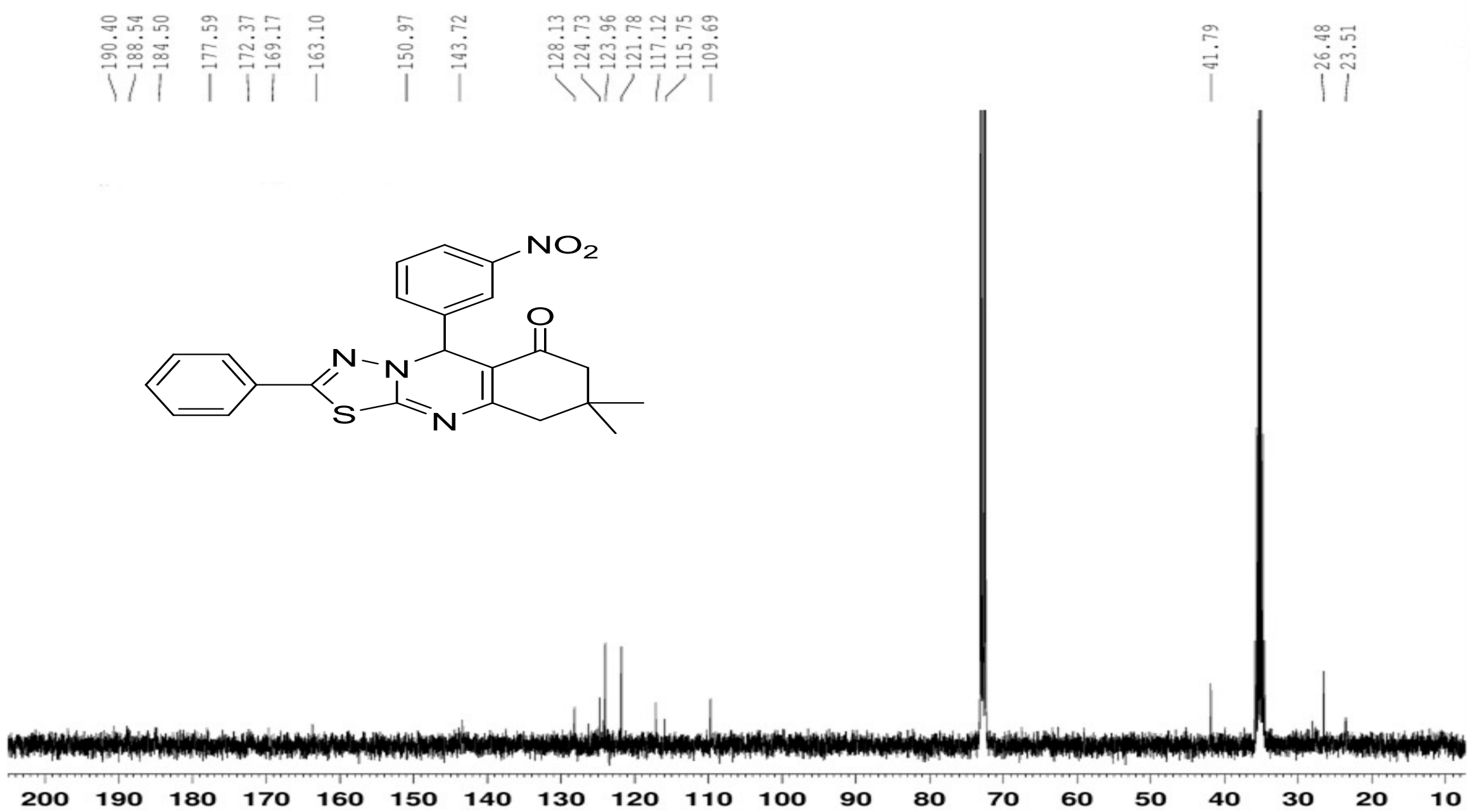

The ^{13}C NMR Spectrum of 5-(3-nitrophenyl)-8,9-dihydro-8,8-dimethyl-2-phenyl-5H-[1,3,4] thiadiazolo[2,3-b]quinazolin-6(7H)-one.

Mass Spectrum of Compound (V-3) S25

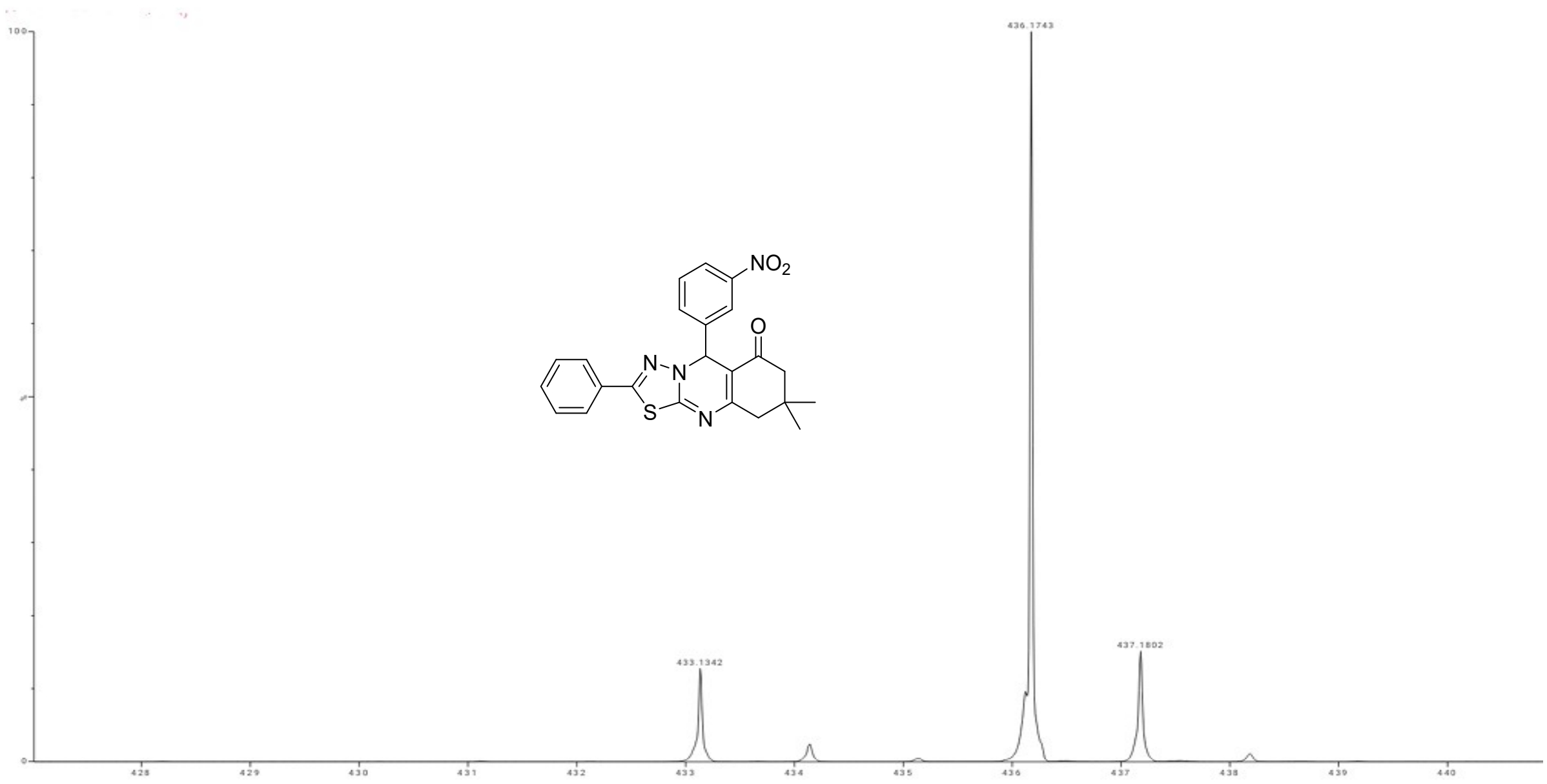

The Mass Spectrum of 5-(3-nitrophenyl)-8,9-dihydro-8,8-dimethyl-2-phenyl-5H-[1,3,4] thiadiazolo[2,3-b]quinazolin-6(7H)-one.

IR Spectrum of Compound (V-4) S26

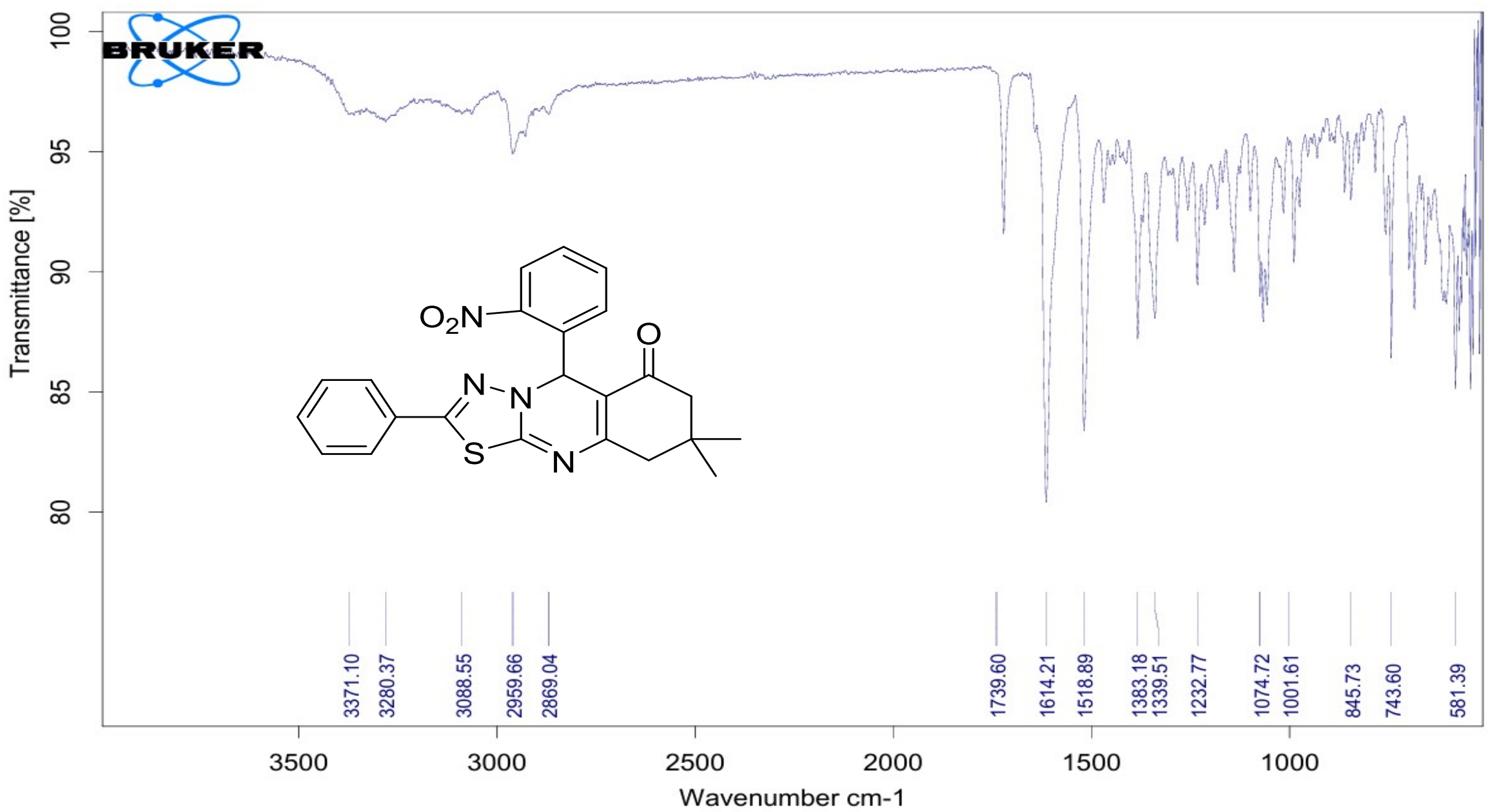

The IR Spectrum of 5-(2-nitrophenyl)-8,9-dihydro-8,8-dimethyl-2-phenyl-5H-[1,3,4]thiadiazolo[2,3-b] quinazolin -6(7H)-one.

^{1}H NMR Spectrum of Compound (V-4) S27

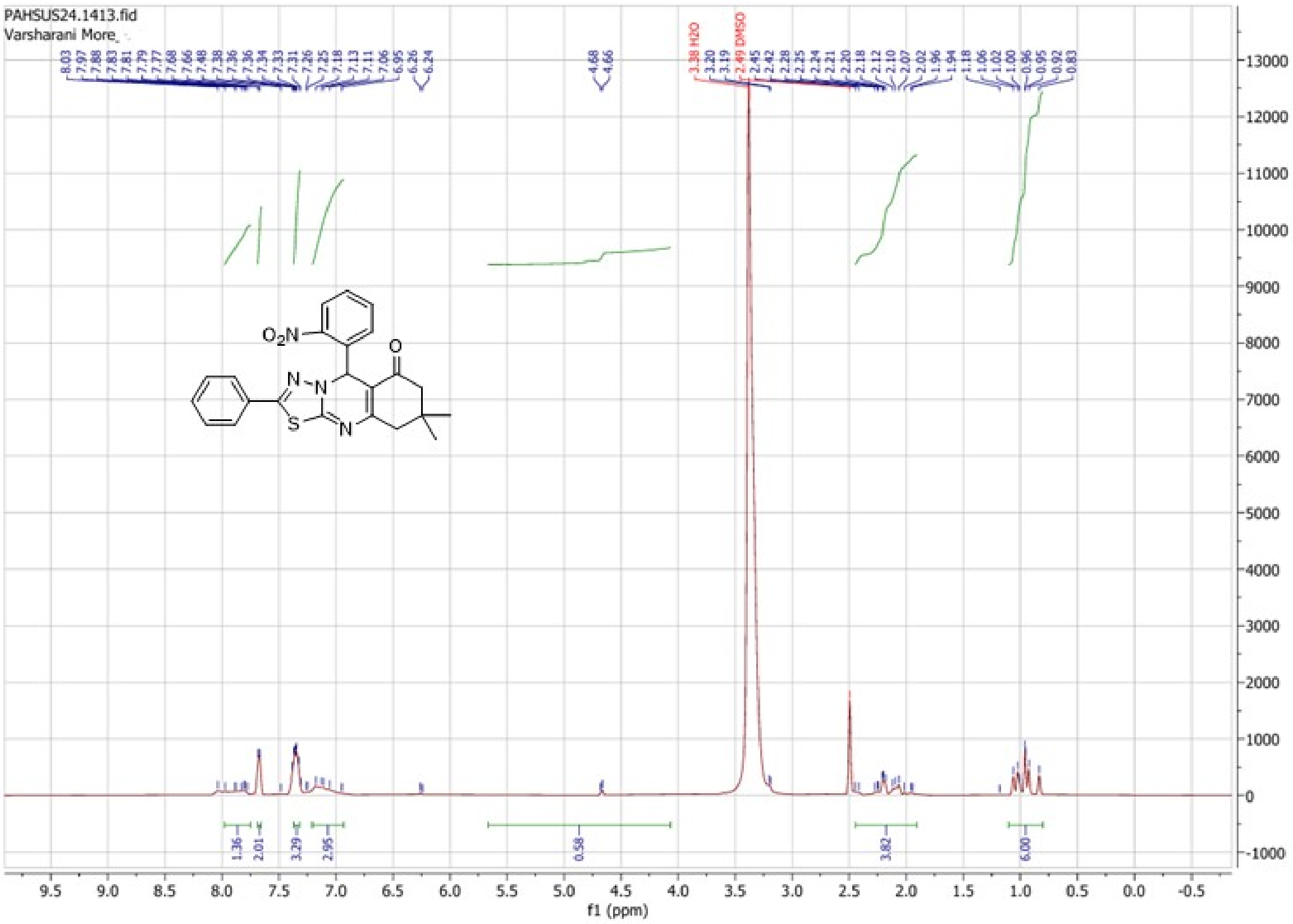

The ^{1}H NMR Spectrum of 5-(2-nitrophenyl)-8,9-dihydro-8,8-dimethyl-2-phenyl-5H-[1,3,4]thiadiazolo[2,3-b] quinazolin -6(7H)-one.

^{13}C NMR Spectrum of Compound (V-4) S28

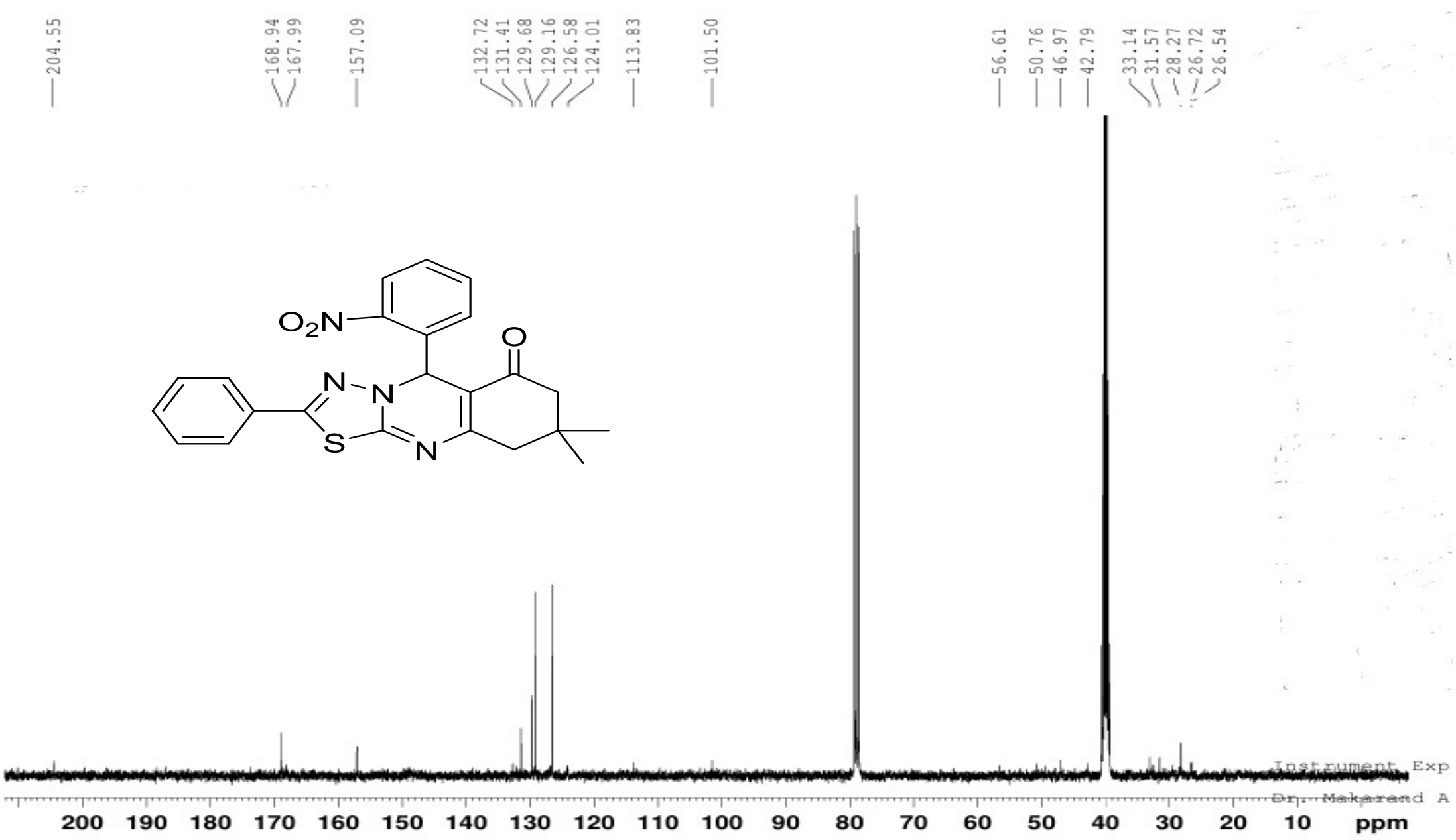

The ^{13}C NMR Spectrum of 5-(2-nitrophenyl)-8,9-dihydro-8,8-dimethyl-2-phenyl-5H-[1,3,4]thiadiazolo[2,3-b] quinazolin -6(7H)-one.

Mass Spectrum of Compound (V-4) S29

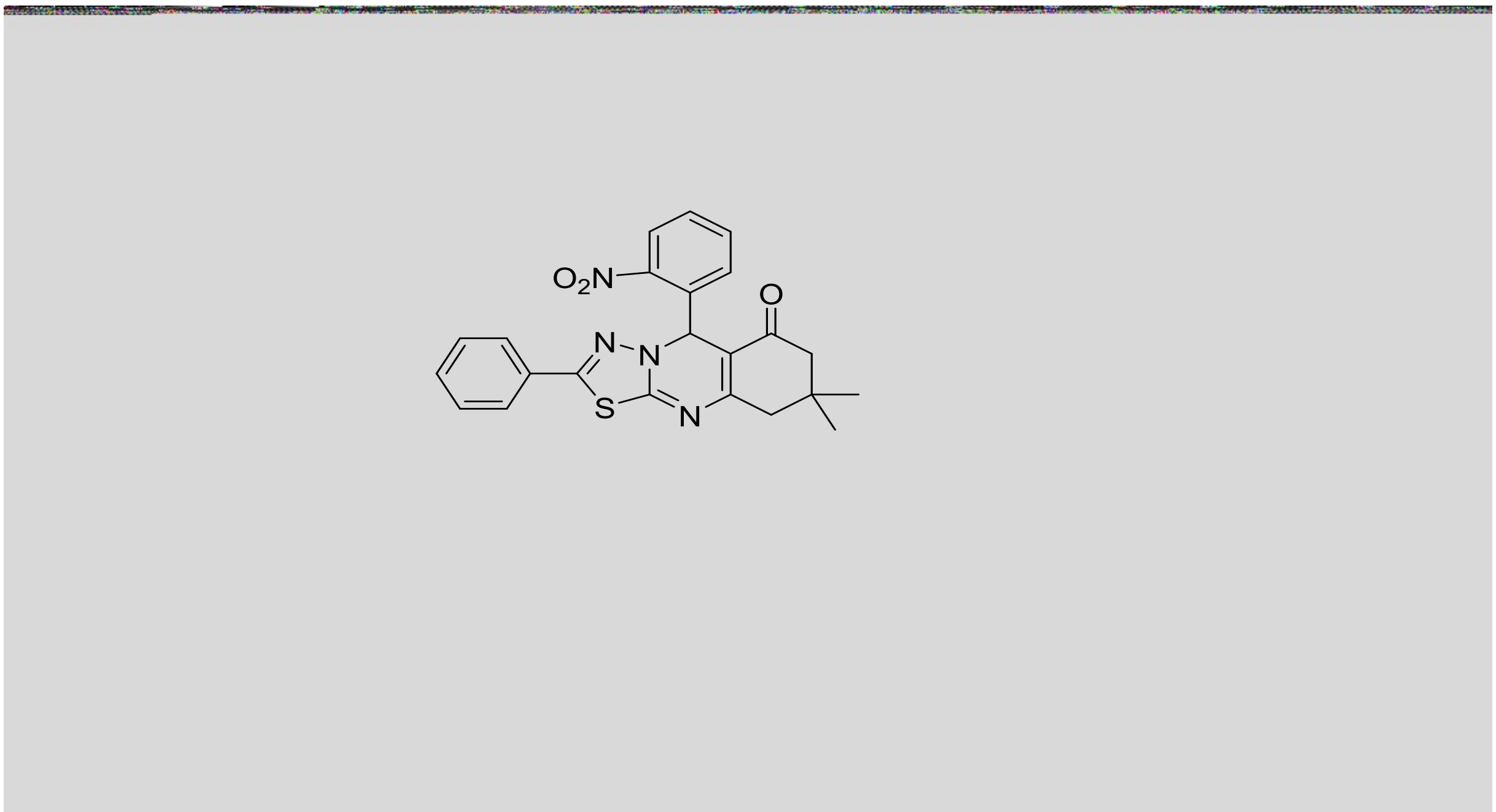

The Mass Spectrum of 5-(2-nitrophenyl)-8,9-dihydro-8,8-dimethyl-2-phenyl-5H-[1,3,4]thiadiazolo[2,3-b] quinazolin -6(7H)-one.

IR Spectrum of Compound (V-5) S30

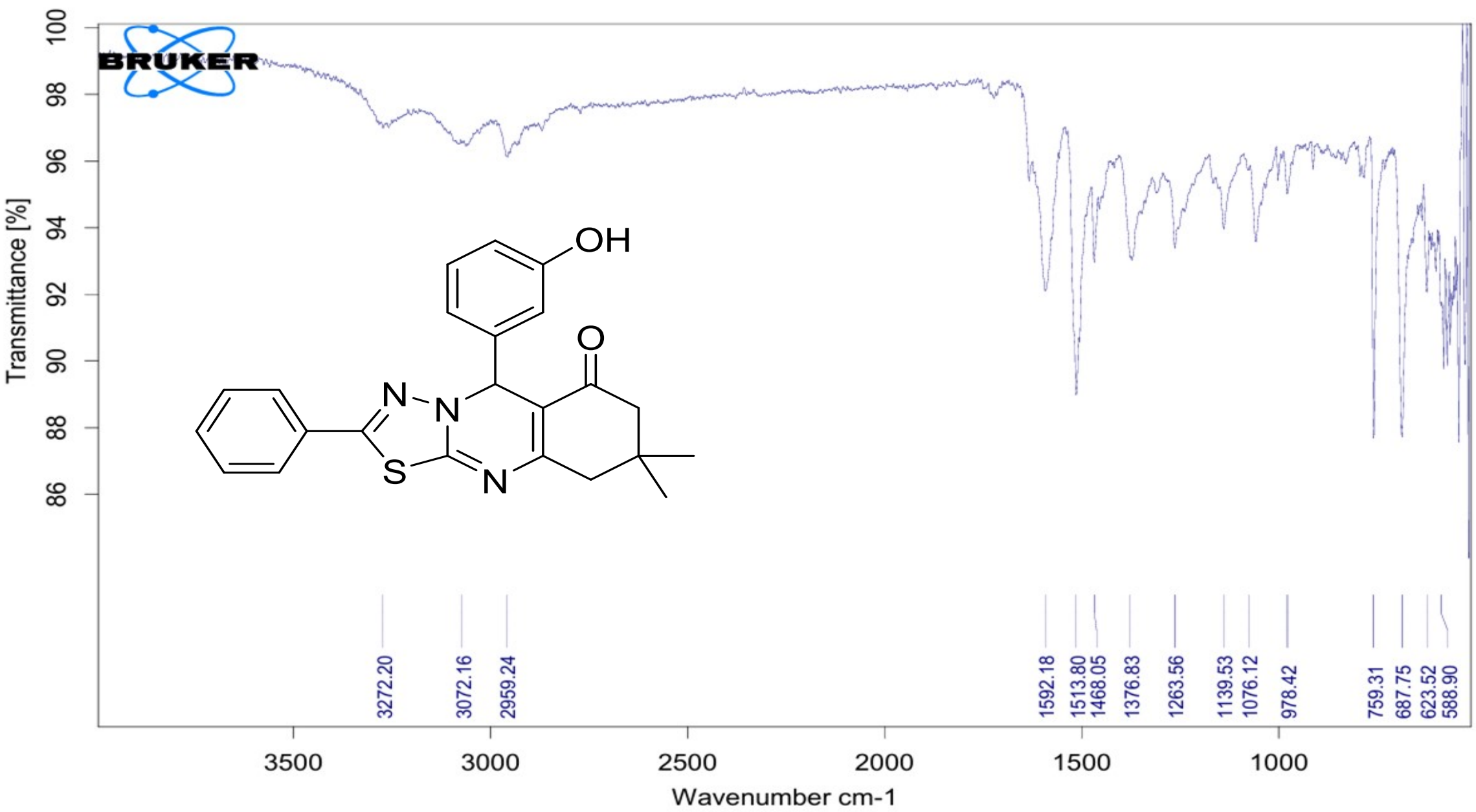

The IR Spectrum of 5-(3-hydroxylphenyl)-8,9-dihydro-8,8-dimethyl-2-phenyl-5H-[1,3,4] thiadiazolo[2,3-b] quinazolin-6(7H)-one.

NMR Spectrum of Compound (V-5) S31

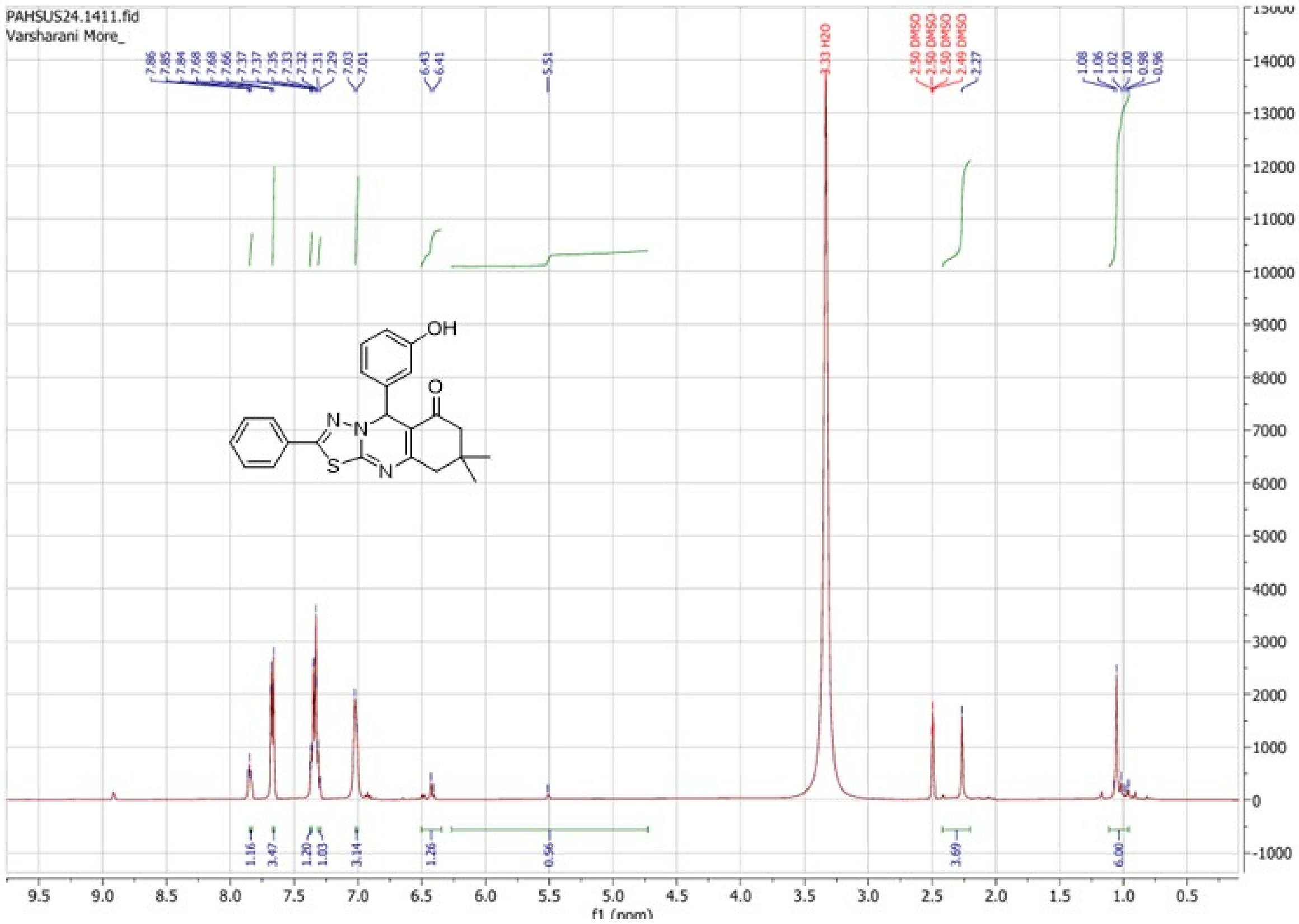

The ^{1}H NMR Spectrum of 5-(3-hydroxylphenyl)-8,9-dihydro-8,8-dimethyl-2-phenyl-5H-[1,3,4] thiadiazolo[2,3-b] quinazolin-6(7H)-one.

^{13}C NMR Spectrum of Compound (V-5) S32

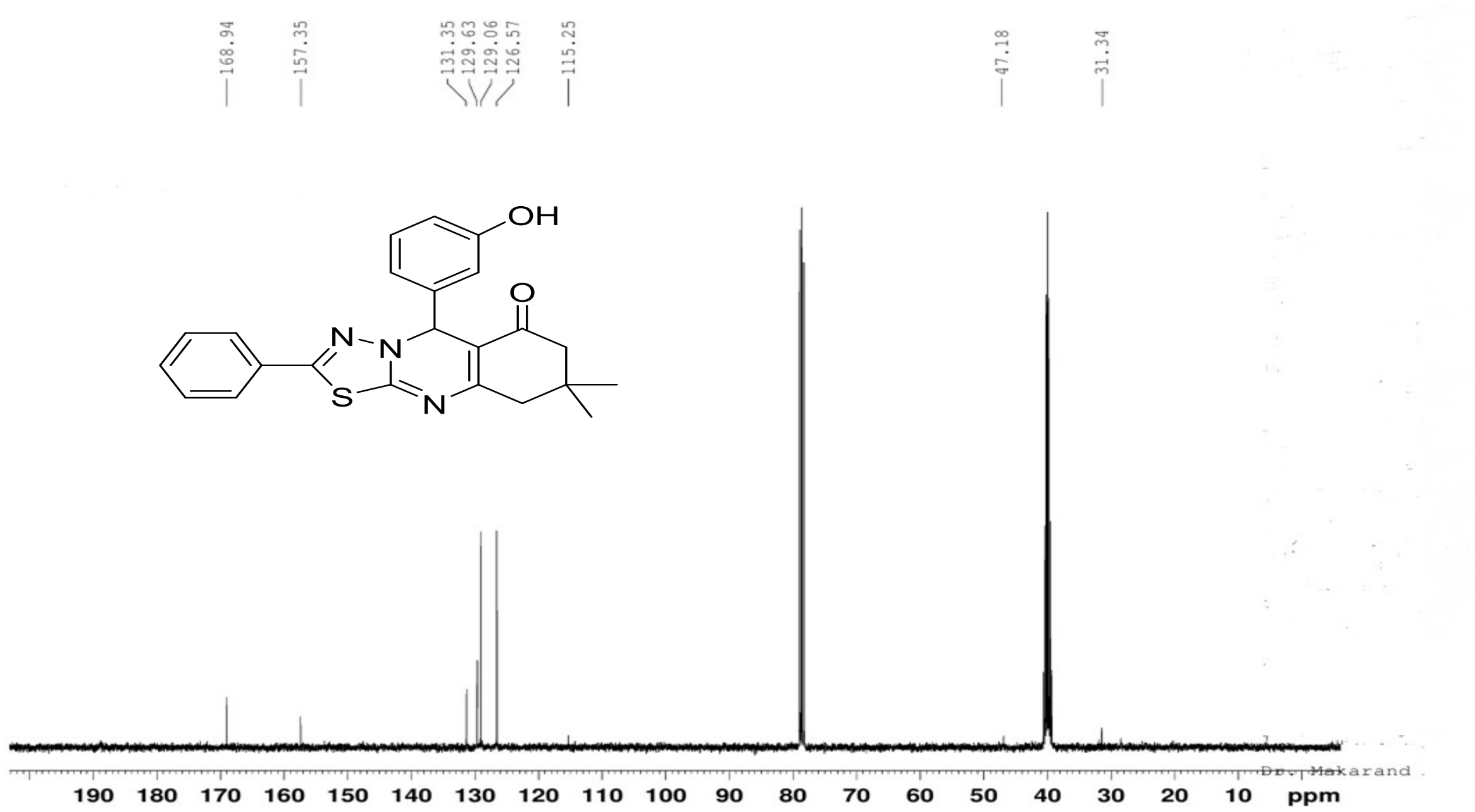

The ^{13}C NMR Spectrum of 5-(3-hydroxylphenyl)-8,9-dihydro-8,8-dimethyl-2-phenyl-5H-[1,3,4] thiadiazolo[2,3-b] quinazolin-6(7H)-one.

IR Spectrum of Compound (V-6) S33

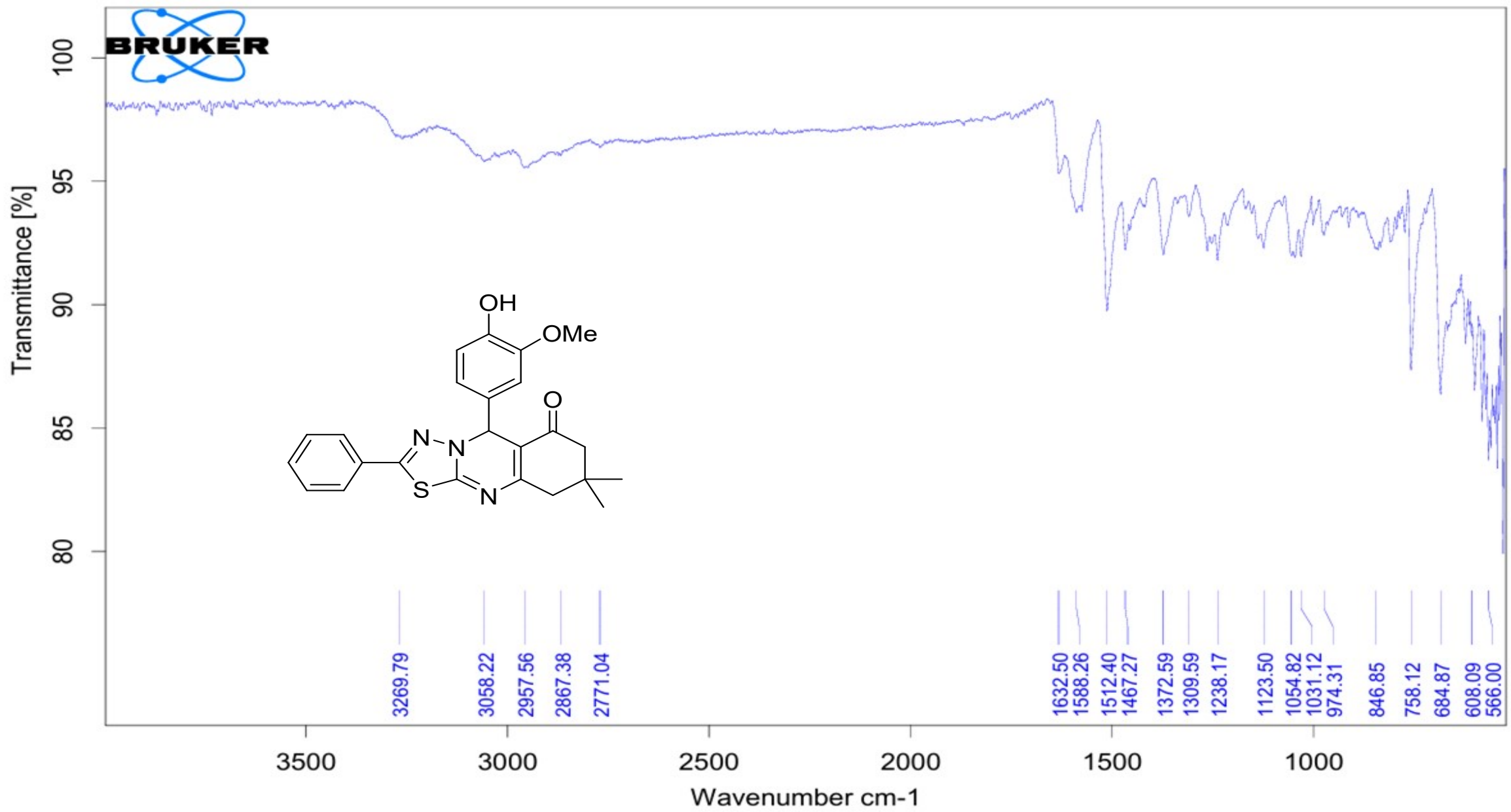

The IR Spectrum of 5-(4-hydroxy-3-methoxyphenyl)-8,9-dihydro-8,8-dimethyl-2-phenyl-5H-[1,3,4] thiadiazolo[2,3-b]quinazolin-6(7H)-one.

^{1}H NMR Spectrum of Compound (V-6) S34

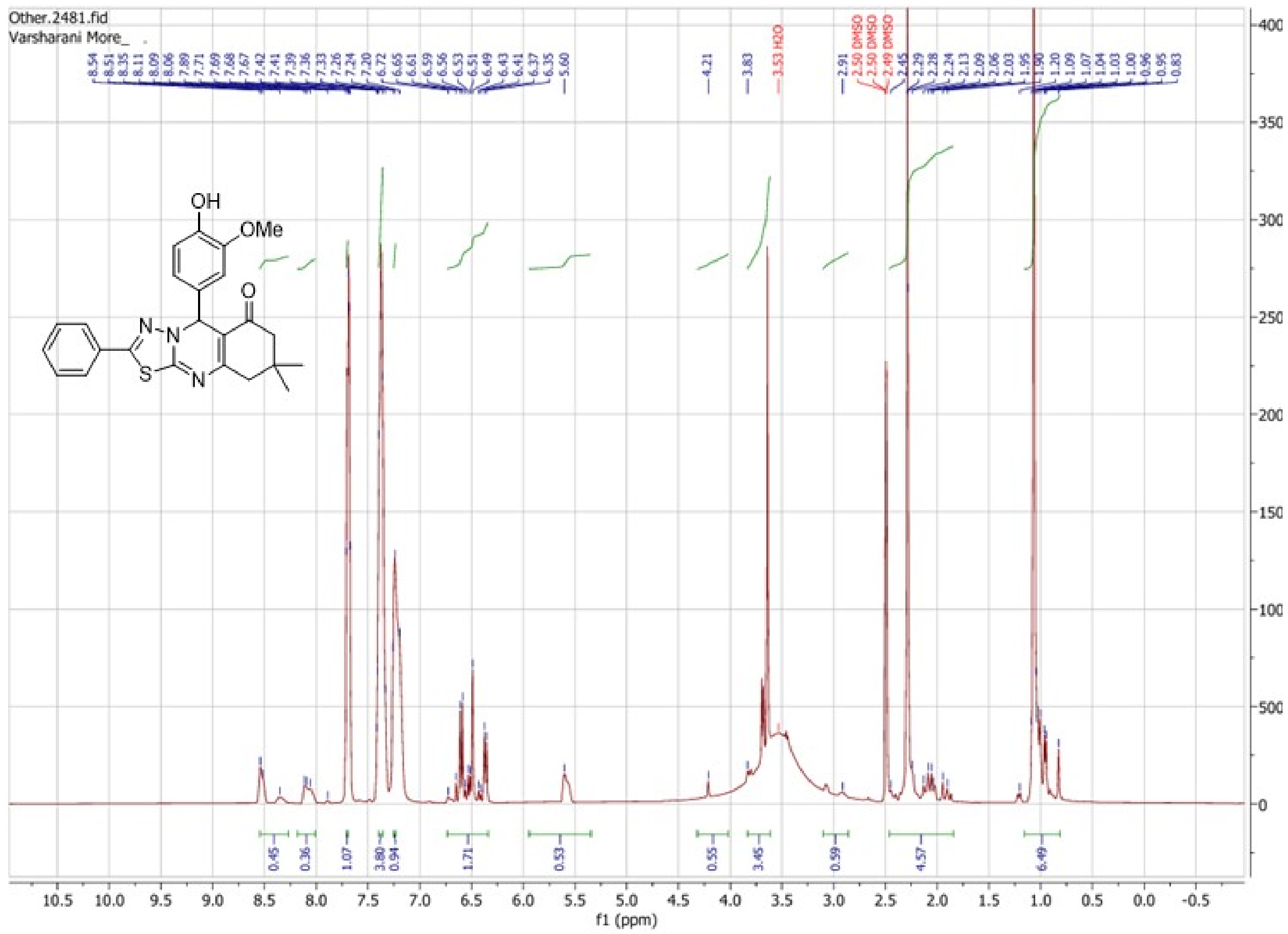

The ^{1}H NMR Spectrum of 5-(4-hydroxy-3-methoxyphenyl)-8,9-dihydro-8,8-dimethyl-2-phenyl-5H-[1,3,4] thiadiazolo[2,3-b]quinazolin-6(7H)-one.

^{13}C NMR Spectrum of Compound (V-6) S35

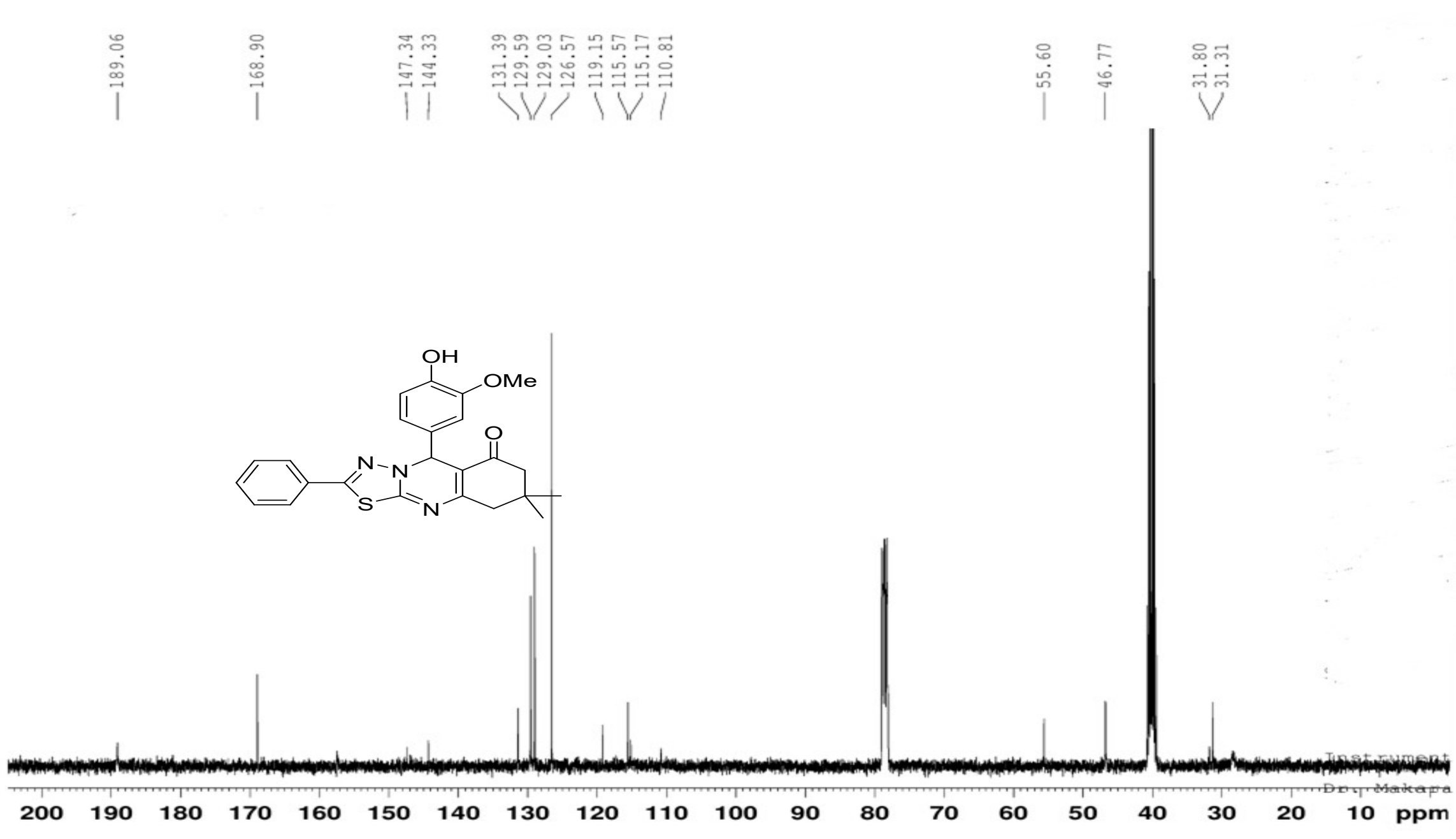

The ^{13}C NMR Spectrum of 5-(4-hydroxy-3-methoxyphenyl)-8,9-dihydro-8,8-dimethyl-2-phenyl-5H-[1,3,4] thiadiazolo[2,3-b]quinazolin-6(7H)-one.

Mass Spectrum of Compound (V-6) S37

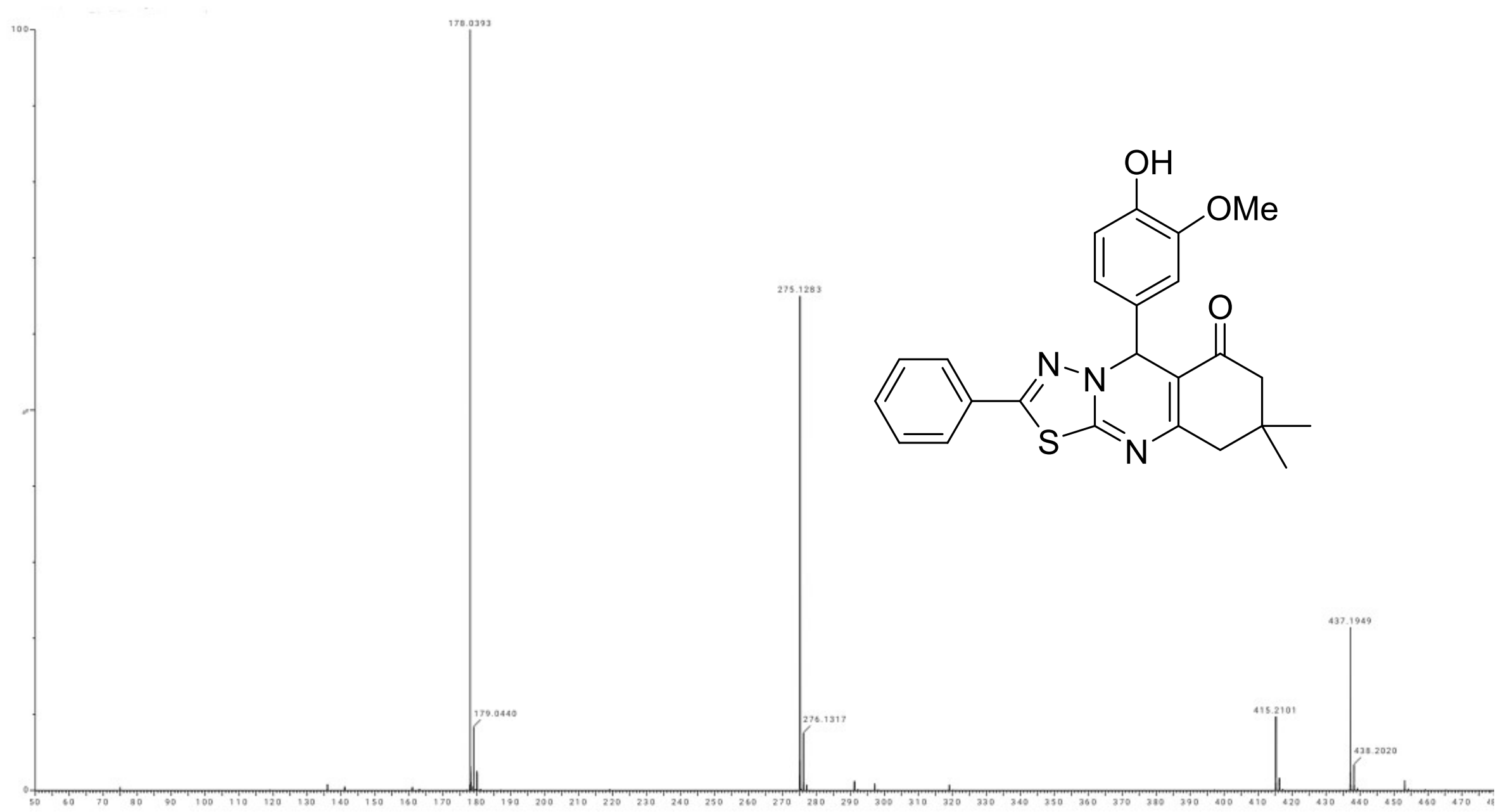

The Mass Spectrum of 5-(4-hydroxy-3-methoxyphenyl)-8,9-dihydro-8,8-dimethyl-2-phenyl-5H-[1,3,4] thiadiazolo[2,3-b]quinazolin-6(7H)-one.
